# Supplementary material for: Identifying actions to foster cross-disciplinary global health research: a mixed-methods qualitative case study of the IMPALA programme on lung health and tuberculosis in Africa
Source: BMJ Open. 2022 Mar 29;12(3):e058126. doi: 10.1136/bmjopen-2021-058126 (PMC8966532; doi:10.1136/bmjopen-2021-058126)
Supplement: Supplementary data [file bmjopen-2021-058126supp001.pdf]

Version 1, 18 March 2018

Multidisciplinary cross-cutting capacity development project (MUDI)

Participant Information Leaflet

-For online survey-

My name is Yan Ding and I work for Capacity Research Unit, Liverpool School of Tropical Medicine. We would like to invite you for an online survey in our research, the MUDI project. Before you decide, we would like you to understand why the research is being done and what it would involve for you Please take time to read the following information carefully.

What is the purpose of the online survey?

The purpose of the survey is to map competencies and experience in conducting multidisciplinary research.

What is the MUDI project?

MUDI is a multidisciplinary capacity development research project under the umbrella of IMPALA which stands for the International Multidisciplinary Programme to Address Lung Health and TB in Africa. IMPALA is a four-year collaborative programme funded by the National Institute for Health Research in the UK.

Why have you been invited?

You have at least one of the following roles in IMPALA:
1) a member of the External Scientific Advisory Panel;
2) a member of the IMPALA leadership team;
3) a member of IMPALA project teams, either from LSTM, or from any collaborating organizations but not for administration and finance;
4) a member of an underpinning collaborations and partnerships of IMPALA

What are the possible benefits of the survey?

This survey will yield valuable information on competencies and experience in conducting multidisciplinary research for MUDI to study the facilitators and barriers of multidisciplinary research at individual level.

Do I have to take part?

It is entirely voluntary. It is up to you to decide whether to take part or not. If you choose to take part, you are free to withdraw from the research study at any time, without giving a reason. If you choose not to participate this will not affect your work or career in any way. You do not have to answer any questions with which you do not feel comfortable.

What will I have to do?

This survey has four components: 1) personal information; 2) education background; 3) previous experience in multidisciplinary research; and 4) self-assessment of capacities in multidisciplinary research. The survey will take about 15 minutes to complete.

Confidentiality

Identifying information such as your name and organization will be asked, as we would like to come back to some of you for semi-structured interviews and ongoing data collection during the implementation of IMPALA. Information on expertise, competencies, gender, age and geographical location provided by this survey will be used to maximize diversity of the interviewees which makes

non-anonymization of the survey necessary. All the answers you provide in this survey will be kept confidential and only shared among the Capacity Research Unit researchers directly involved in the MUDI project. The survey data will be reported in a summary fashion only and will not identify any individual person.

Will I be reimbursed for my time?
You will not be paid for being part of this project.

Will you participate in this study? Yes or No
If yes, please go to the next page and indicate your consent in the tick box provided.

For further details, or if you have any questions or want to file a complaint about the research you may contact:

Organisation responsible for the study:

Dr. Yan Ding

Liverpool School of Tropical Medicine, UK.

E-mail: yan.ding@lstmed.ac.uk.

The LSTM Research Ethics Committee

E-mail: lstmrec@lstmed.ac.uk
