# Supplementary material for: Identifying actions to foster cross-disciplinary global health research: a mixed-methods qualitative case study of the IMPALA programme on lung health and tuberculosis in Africa
Source: BMJ Open. 2022 Mar 29;12(3):e058126. doi: 10.1136/bmjopen-2021-058126 (PMC8966532; doi:10.1136/bmjopen-2021-058126)
Supplement: Supplementary data [file bmjopen-2021-058126supp002.pdf]

Version 1, 18 March 2018

ELECTRONIC CONSENT FORM*
-online survey-

**Study Title: Competencies and experience in multidisciplinary research**
**Principal Investigator: Prof. Imelda Bates, Liverpool School of Tropical Medicine, UK.**

☐ I have read the information sheet concerning this study and I understand what will be required of me if I take part in this study. I understand that at any time, I may withdraw from this study without giving a reason and without affecting my participation in any research activities or consortium I am involved in. I agree to take part in this study.

***To be inserted as the third page of the online survey (following information sheet).**
