# Supplementary material for: Identifying actions to foster cross-disciplinary global health research: a mixed-methods qualitative case study of the IMPALA programme on lung health and tuberculosis in Africa
Source: BMJ Open. 2022 Mar 29;12(3):e058126. doi: 10.1136/bmjopen-2021-058126 (PMC8966532; doi:10.1136/bmjopen-2021-058126)
Supplement: Supplementary data [file bmjopen-2021-058126supp003.pdf]

Confidence and experience in multidisciplinary research

Page 1: Welcome!

IMPALA MUDI beginning-of-project survey:

Experience and Confidence in Multidisciplinary Research

Version 7, 21 May 2018

What is your role in IMPALA? (screening question) ✱ *Required*

- a member of the External Scientific Advisory Panel
- a member of the IMPALA leadership team
- a member of IMPALA project teams or a collaborator but not purely involved in administration and finance
- an administration or finance staff member
- I am not involved in IMPALA
- Other

If you selected Other, please specify:

Ding Y, *et al. BMJ Open* 2022; 12:e058126. doi: 10.1136/bmjopen-2021-058126

Page 2: Participant Information Leaflet

Version 2, 18 May 2018

Multidisciplinary cross-cutting capacity development project (MUDI)

Participant Information Leaflet

-Beginning-of-project survey-

My name is Yan Ding and I work for the Capacity Research Unit, Liverpool School of Tropical Medicine. We would like to invite you to participate in an online survey in our research, the MUDI project. Before you decide, we would like you to understand why the research is being done and what it would involve for you Please take time to read the following information carefully.

What is the purpose of the online survey?

The purpose of the survey is to map experience and confidence in conducting multidisciplinary research.

What is the MUDI project?

MUDI is a multidisciplinary capacity development research project under the umbrella of IMPALA (International Multidisciplinary Programme to Address Lung Health and TB in Africa). IMPALA is a four-year collaborative programme funded by the National Institute for Health Research in the UK.

Why have you been invited?

You have at least one of the following roles in IMPALA:

1) a member of the External Scientific Advisory Panel;

Ding Y, *et al. BMJ Open* 2022; 12:e058126. doi: 10.1136/bmjopen-2021-058126

2) a member of the IMPALA leadership team;

3) a member of IMPALA project teams or a collaborator.

What are the possible benefits of the survey?

This survey will yield valuable information on experience and confidence in conducting multidisciplinary research for MUDI to study the facilitators and barriers of multidisciplinary research at individual level.

Do I have to take part?

It is entirely voluntary. It is up to you to decide whether to take part or not. If you choose to take part, you are free to withdraw from the research study at any time, without giving a reason. If you choose not to participate this will not affect your work or career in any way. You do not have to answer any questions with which you do not feel comfortable.

What will I have to do?

This survey has four components: 1) personal information; 2) education background; 3) previous experience in multidisciplinary research; and 4) confidence in multidisciplinary research. The survey will take about 15 to 20 minutes to complete.

Confidentiality

Identifying information such as your name and organization will be asked, as we would like to come back to some of you for semi-structured interviews and ongoing data collection during the implementation of IMPALA. Information on experience, confidence, gender, age and geographical location provided by this survey will be used to maximize diversity of the interviewees which makes non-anonymization of the survey necessary. All the answers you provide in this survey will be kept confidential and only shared among the Capacity Research Unit researchers directly involved in the MUDI project. The survey data will be reported in a summary fashion only and will not identify any individual person. The anonymised results of the survey will be shared through IMPALA.

Will I be reimbursed for my time?

You will not be paid for being part of this project.

Will you participate in this study? Yes or No

If yes, please go to the next page and indicate your consent in the tick box provided.

For further details, or if you have any questions or want to file a complaint about the research you may contact:

Organisation responsible for the study:

Dr. Yan Ding

Liverpool School of Tropical Medicine, UK.

E-mail: yan.ding@lstmed.ac.uk.

The LSTM Research Ethics Committee:

E-mail: lstmrec@lstmed.ac.uk

Do you agree to take this online survey? ✱ *Required*

- ○ Yes
- ○ No

Page 3: Electronic Consent Form

Study Title: Competencies and experience in multidisciplinary research

Principal Investigator: Prof. Imelda Bates, Liverpool School of Tropical Medicine, UK.

Thank you for agreeing to take this survey. Please tick the statement to continue.

○ I have read the information sheet concerning this study and I understand what will be required of me if I take part in this study. I understand that at any time, I may withdraw from this study without giving a reason and without affecting my participation in any research activities or consortium I am involved in. I agree to take part in this study.

Page 4: Main body of the survey

Section 1: Personal information

First Name, Last Name ✱ *Required*

Your gender: ✱ *Required*

- Female
- Male
- Prefer not to say

Which year were you born? ✱ *Required*

Your current country of residence: ✱ *Required*

Organization where you work? ✱ *Required*

Ding Y, *et al. BMJ Open* 2022; 12:e058126. doi: 10.1136/bmjopen-2021-058126

Job title at your organization ✱ *Required*

Your main responsibility in IMPALA ✱ *Required*

Section 2: Education background

What degrees have you completed? Please choose multiple answers if you have more than one answer ✱ *Required*

- ☐ Bachelor’s degree
- ☐ Master’s degree
- ☐ Doctorate degree
- ☐ Other

If you selected Other, please specify:

Please write down the discipline (s) of **all** degrees you have completed and separate them by comma. **Discipline** refers to a particular branch of learning or body of knowledge such as history, sociology, economics. ✱ *Required*

Which of the following option do you think applies to your professional background? ✱ *Required*

- Unidisciplinary
- Multidisciplinary

Section 3: Experience in multidisciplinary research

Multidisciplinary research in this study is research that uses knowledge, study design and methodology from multiple disciplines regardless of the extent of integration of disciplinary-specific theories, concepts, and approaches to address common problems.

Did you have **previous experience** of the following activities before participated in the IMPALA programme? (check the one that best applies)

Creating a network for further collaborations ✱ *Required*

- Yes
- No
- I don't remember

Ding Y, *et al. BMJ Open* 2022; 12:e058126. doi: 10.1136/bmjopen-2021-058126

If yes, please write down one example:

Extending a network for further collaborations ✱ *Required*

- Yes
- No
- I don't remember

If yes, please write down one example:

Reading academic articles **outside** your discipline? ✱ *Required*

- Yes
- No
- I don't remember

If yes, please specify the disciplines:

Have you drafted funding proposals for multidisciplinary research projects /programs in partnership with scholars from other disciplines? ✱ *Required*

- Yes
- No
- I don't remember

Proactively seeking an exchange of theories, concepts, and approaches with those from other disciplines? ✱ *Required*

- Yes
- No
- I don't remember

If yes, please specify the disciplines:

Have you modified your own perspectives on research questions or research plan as a result of interactions with colleagues from fields other than your own? ✱ *Required*

Ding Y, *et al. BMJ Open* 2022; 12:e058126. doi: 10.1136/bmjopen-2021-058126

- Yes
- No
- I don't remember

If yes, please write down one example:

Have you coordinated fieldwork with team members in a multidisciplinary research to maximize the leverage? ✱ *Required*

- Yes
- No
- I don't remember

If yes, please write down one example:

Have you combined research outcomes of contributing disciplines in a multidisciplinary

Ding Y, *et al. BMJ Open* 2022; 12:e058126. doi: 10.1136/bmjopen-2021-058126

research project into a final technical output/publication? ✱ *Required*

- Yes
- No
- I don't remember

If yes, please write down one example:

Have you disseminated multidisciplinary research results to **policy makers**? ✱ *Required*

- Yes
- No
- I don't remember

If yes, please write down one example:

Ding Y, *et al. BMJ Open* 2022; 12:e058126. doi: 10.1136/bmjopen-2021-058126

Have you advocated for multidisciplinary research? ✱ *Required*

- Yes
- No
- I don't remember

If yes, please write down one example:

Section 4: Confidence in multidisciplinary research

Please choose one response for each question that best describes your confidence.

5 means extremely confident and 1 extremely unconfident

Do you feel confident to do the following activities?

To create a network for further collaborations ✱ *Required*

- 5. Extremely confident
- 4.
- 3.
- 2.
- 1. Extremely unconfident

To extend a network for further collaborations ✱ *Required*

- 5. Extremely confident
- 4.
- 3.
- 2.
- 1. Extremely unconfident

To draft funding proposals for multidisciplinary research projects /programs in partnership with scholars from other disciplines ✱ *Required*

- 5. Extremely confident
- 4.
- 3.
- 2.
- 1. Extremely unconfident

To establish trust among research team members ✱ *Required*

- 5. Extremely confident
- 4.
- 3.
- 2.
- 1. Extremely unconfident

To articulate your research questions, theories, concepts and approaches orally to those from other disciplines? ✱ *Required*

Ding Y, *et al. BMJ Open* 2022; 12:e058126. doi: 10.1136/bmjopen-2021-058126

- 5. Extremely confident
- 4.
- 3.
- 2.
- 1. Extremely unconfident

To engage colleagues from other disciplines to gain their perspectives on **research questions and research plan** ✱ *Required*

- 5. Extremely confident
- 4.
- 3.
- 2.
- 1. Extremely unconfident

To modify your own perspectives on **research questions and plan** as a result of interactions with those from other disciplines ✱ *Required*

- 5. Extremely confident
- 4.
- 3.
- 2.
- 1. Extremely unconfident

To learn new knowledge and extend the width of your knowledge beyond your primary discipline ✱ *Required*

Ding Y, *et al. BMJ Open* 2022; 12:e058126. doi: 10.1136/bmjopen-2021-058126

- 5. Extremely confident
- 4.
- 3.
- 2.
- 1. Extremely unconfident

To actively participate in group discussions/meetings in a multidisciplinary research ✱ *Required*

- 5. Extremely confident
- 4.
- 3.
- 2.
- 1. Extremely unconfident

To coordinate fieldwork with team members to maximize the leverage ✱ *Required*

- 5. Extremely confident
- 4.
- 3.
- 2.
- 1. Extremely unconfident

To interact with non-academic stakeholders in general in a multidisciplinary research ✱ *Required*

- 5. Extremely confident

- 4.
- 3.
- 2.
- 1. Extremely unconfident

To combine research outcomes of contributing disciplines into a final technical output ✱ *Required*

- 5. Extremely confident
- 4.
- 3.
- 2.
- 1. Extremely unconfident

To share research from your discipline in language understandable by those outside your discipline ✱ *Required*

- 5. Extremely confident
- 4.
- 3.
- 2.
- 1. Extremely unconfident

To disseminate multidisciplinary research results to policy makers ✱ *Required*

- 5. Extremely confident
- 4.

- 3.
- 2.
- 1. Extremely unconfident

To advocate for multidisciplinary research ✱ *Required*

- 5. Extremely confident
- 4.
- 3.
- 2.
- 1. Extremely unconfident

Ding Y, *et al. BMJ Open* 2022; 12:e058126. doi: 10.1136/bmjopen-2021-058126

Page 5: Thank you for completing the survey

We really appreciate your input and time.

If you have any question, please feel free to contact Yan at yan.ding@lstmed.ac.uk**.**

Wish you all the best.

Ding Y, *et al. BMJ Open* 2022; 12:e058126. doi: 10.1136/bmjopen-2021-058126
