# Supplementary material for: Identifying actions to foster cross-disciplinary global health research: a mixed-methods qualitative case study of the IMPALA programme on lung health and tuberculosis in Africa
Source: BMJ Open. 2022 Mar 29;12(3):e058126. doi: 10.1136/bmjopen-2021-058126 (PMC8966532; doi:10.1136/bmjopen-2021-058126)
Supplement: Supplementary data [file bmjopen-2021-058126supp004.pdf]

Version 4, 20 March 2018

IMPALA

Multidisciplinary cross-cutting capacity development project (MUDI)

Topic guides for semi-structured interviews, baseline survey

IMPALA team structure

Contents

1 Background information to assist the identification of MUDI interviewees 1

2 A topic guide for interviews with a member from the external scientific advisory board 3

3 A topic guide for interviews with the directors of IMPALA 4

4 A topic guide for interviews with researchers of the two applied projects, including from LSTM (except IMPALA Directors), from project country teams in Sudan, Tanzania and Uganda, and from institutions in other African countries 7

1 Background information to assist the identification of MUDI interviewees

MUDI takes the two phase-1 applied research projects within IMPALA as case studies. We would like to interview a member of the external scientific advisory board, the IMPALA directors, and researchers working on the two projects, including these based in LSTM, from project country teams and from institutions in other countries in Africa. Figure 1 presents the team structure of the two phase-1 applied research projects within IMPALA to assist the identification of interviewees.

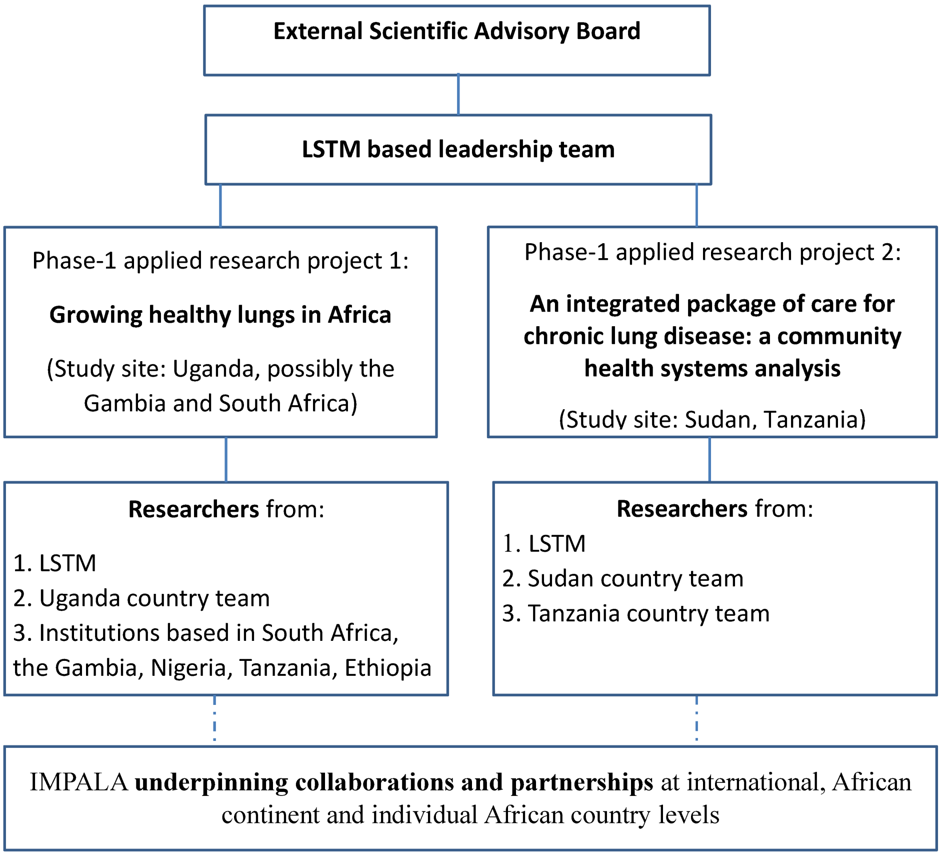

Figure 1: The team structure of the two phase-1 applied research projects within IMPALA

2 A topic guide for interviews with a member from the external scientific advisory board

1) **Introduction**
    - to be explained to interviewees
    - will first go through the information sheet with the interviewees
    - ask for consent to have the interviews & permission for audio-recording

2) **Background**
    - Academic qualification (bachelor degree? masters' degree? doctorate? Other specialist training?)
    - What's your role within your organization?
    - Personal research and work experience in multi-, inter- and trans-disciplinary collaboration
    - Could you please tell me about your role as a member of the external scientific advisory board member? Your main responsibilities and your participation so far.
3) **Examples of research involving multiple disciplines of where it worked well and not well in your experience**.
4) **IMPALA**
    - What's your vision for IMPALA?
    - What are the collaborations among disciplines do you perceive in IMPALA?
    - How do you define multidisciplinary research?
    - In MUDI, we define **multidisciplinary research** in this study is research that uses knowledge, study design and methodology from multiple disciplines, and which is based on a shared conceptual framework drawing together disciplinary-specific theories, concepts, and approaches to address common problems.

      What kind of multidisciplinary research do you think the two phase-1 projects respectively are at their current stages in the abovementioned three kinds of situation? Is there any observed movement of the two projects among the three kinds of situation for multidisciplinary research according to your observation so far?
    - According to your experience and knowledge, what do you think would be the facilitator and barriers of a multi-disciplinary collaboration?
5) **Strategy in influencing practice and policy**
    - Would you put applying knowledge from research to **practice** as a vision, or an aim?
    - Would you put applying knowledge from research to **policy** as a vision, or an aim?
    - Do you think it is possible to demonstrate IMPALA's impact on practice and policy?
    - What would be your suggestions?
6) Any other things you would like to add?

3 A topic guide for interviews with the directors of IMPALA

1) **Introduction**
2) **Personal background**
    - Academic qualification (bachelor degree? masters' degree? doctorate? other specialist training?)
    - Personal research and work experience in multi-, inter- and trans-disciplinary collaboration
3) **IMPALA**
    - What disciplines are involved in IMPALA?
    - How did you identify these disciplines for a collaboration?
    - Why such a multi-disciplinary collaboration?
    - What do you want to achieve through such a collaboration?
4) **Facilitating multidisciplinary research**
    - How do you facilitate multidisciplinary research?
      Probes:
      What strategy?
      What techniques?
5) **Influencing practice and policy**
    - Would you put applying knowledge from research to **practice as a vision, or an aim for IMPALA**?
    - Would you put applying knowledge from research to **policy as a vision, or an aim for IMPALA**?
6) **Your participation in the phase-1 project (s)**
    - Research questions and decide to work together
      -What specific research questions are you addressing to achieve the project aim?
      -Do you need other disciplines to answer the research questions? If so, which ones? Why?

      -Have you modified the research questions for this project as a result of interactions with colleagues from fields other than your own? If yes, could you tell me more?
      -Do you have any pre-experience in modifying your own research questions as a result of interactions with colleagues from fields other than your own? If yes, could you please give me examples?

    - Bring in knowledge, study design and methodology from multiple disciplines

      -Have you contributed to the study design of the project? If so, could you please tell me more about your input?
      -Have you contributed to the methodology of the project? If so, could you please tell me more about your input?
      -Who else also contributed to the study design?
      -Who else also contributed to the methodology?
      -In this project, have you modified your study design as a result of interactions with colleagues from fields other than your own? If so, could you tell me more?

-In this project, have you modified your own research methods as a result of interactions with colleagues from fields other than your own? If so, could you tell me more?
-How do you feel about the knowledge, study design and methodology brought in from other disciplines?

- -How do you define multidisciplinary research?
- Researchers from different disciplines work together in one project to address a common problem. There are different ways of working together.
  For example: researchers with different disciplinary background address different aspects of a problem based on their disciplinary background. They work independently from researchers of other disciplinary background. As each discipline addresses one aspect of the problem, together, the problem has been addressed in a more comprehensive way.
  Another example: researchers are still in charge of studying different aspects of a problem based on their disciplinary background. Besides this, they have interactions and influence each other's study design and project activities.
  What's the way that researchers from this project work together?

7) Involvement and interaction with non-academic stakeholders of the project

- Is there any non-academic stakeholder that involve in the project so far?
- If yes:
  -Who are they?
  -Do you know their disciplinary background?
  -What project activities have they participate in? Since when?
  -Any perspectives from them have been integrated in the project? If so, what?
- Collaboration history
  -Among all researchers or called project team members in this phase-1 applied research project, is there any collaboration between you and any of your project team members before this project? (Probe: from project members from other organizations, project member with different disciplinary background and from the same organization)
  If there are some, could you give me examples, including research topics and how long you worked together.

8) Facilitators and barriers

- From all your previous experience, what things have you found make it easier for you to work with colleagues from other disciplines, and why? (probe: institutional level, program level, project level, individual level)
- Also from all your previous experience, what things have you found make it difficult for you to work with colleagues from other disciplines, and why? (probe: institutional level, program level, project level, individual level)
- Have you encountered any barriers related to collaboration among multiple disciplines when you participated in designing the project in the intervention countries? If yes, what are they? How do you cope with them so far?

9) Expectations

- What's your expectations for IMPALA?
- Expectations for the project (s)?
- What would you expect for yourself to achieve in participating in this multidisciplinary research?
  (probe: experiences? Competencies?)
- Do you plan to disseminate your research findings in this phase-1 project to researchers in other disciplines? Why?
- How do you think collaboration with other disciplines' influence on your research career in terms of academic performance? (at your institution, in your country and at global level)

10) Next steps

- What kind of competencies in multidisciplinary research do you feel like to have further improvement
  Probes: 1) personal knowledge and skills in investigating a research question by integrating theories and methods of other disciplines into your primary discipline; 2) communicating with researchers from other disciplines, for example describing your research perspective to them, disseminating your research among them; and 3) interacting with researchers from other disciplines, such as writing a joint proposal, attending scholarly presentations in other disciplines.
- How will you work together with other researchers in the coming year for the phase-1 project? (divisions of responsibilities and joint efforts? Communication plan? Interaction?)
- with non-academic stakeholders?

11) Any other things you would like to add?

4 A topic guide for interviews with researchers of the two applied projects, including from LSTM (except IMPALA Directors), from project country teams in Sudan, Tanzania and Uganda, and from institutions in other African countries

1) **Introduction**
   - To be explained to interviewees
   - Will first go through the information sheet with the interviewees
   - Ask for consent to have the interviews & permission for audio-recording
2) **Background**
   - Academic qualification (bachelor degree? masters' degree? doctorate? other specialist training?)
   - What's your role within your organization? Within IMPALA? Within the two phase-1 projects
   - Personal research and work experience in multi-, inter- and trans-disciplinary collaboration
3) **IMPALA, phase-1 project (s) (discipline related)**
   - R<u>esearch questions and decide to work together</u>

     -What specific research questions are you addressing to achieve the project aim?
     -Do you need other disciplines to answer the research questions? If so, which ones? Why?
     -Have you modified the research questions for this project as a result of interactions with colleagues from fields other than your own? If yes, could you tell me more?
     -Do you have any pre-experience in modifying your own research questions as a result of interactions with colleagues from fields other than your own? If yes, could you please give me examples?

   - <u>Bring in knowledge, study design and methodology from multiple disciplines</u>

     -Have you contributed to the study design of the project? If so, could you please tell me more about your input?
     -Have you contributed to the methodology of the project? If so, could you please tell me more about your input?
     -Who else also contributed to the study design?
     -Who else also contributed to the methodology?
     -In this project, have you modified your study design as a result of interactions with colleagues from fields other than your own? If so, could you tell me more?
     -In this project, have you modified your own research methods as a result of interactions with colleagues from fields other than your own? If so, could you tell me more?
     -How do you feel about the knowledge, study design and methodology brought in from other disciplines?

   - How do you define multidisciplinary research?

- Researchers from different disciplines work together in one project to address a common problem. There are different ways of working together.
  For example: researchers with different disciplinary background address different aspects of a problem based on their disciplinary background. They work independently from researchers of other disciplinary background. As each discipline addresses one aspect of the problem, together, the problem has been addressed in a more comprehensive way.
  Another example: researchers are still in charge of studying different aspects of a problem based on their disciplinary background. Besides this, they have interactions and influence each other's study design and project activities.
  What's the way that researchers from this project work together?

  c
  -?

4) Involvement and interaction with non-academic stakeholders of the project

- Is there any non-academic stakeholder that involve in the project so far?
- If yes:
  -Who are they?
  -Do you know their disciplinary background?
  -What project activities have they participate in? Since when?
  -Any perspectives from them have been integrated in the project? If so, what?
- Collaboration history
  -Among all researchers or called project team members in this phase-1 applied research project, is there any collaboration between you and any of your project team members before this project? (Probe: project members from other organizations, project member with different disciplinary background and from the same organization)
  If there are some, could you give me examples, including research topics and how long you worked together.

5) Facilitators and barriers

- From all your previous experience, what things have you found make it easier for you to work with colleagues from other disciplines, and why? (probe: institutional level, program level, project level, individual level)
- Also from all your previous experience, what things have you found make it difficult for you to work with colleagues from other disciplines, and why? (probe: institutional level, program level, project level, individual level)
- Have you encountered any barriers related to collaboration among multiple disciplines when you participated in designing the project in the intervention countries? If yes, what are they? How do you cope with them so far?

6) Expectations

- What's your expectations for IMPALA?
- Expectations for the project (s)?
- What would you expect for yourself in participating in the multidisciplinary research project?
  (probe: experience? competencies?)
- Do you plan to disseminate your research findings in this phase-1 project to researchers in other disciplines? Why?

- How do you think collaboration with other disciplines' influence on your research career in terms of academic performance? (at your institution, in your country and at global level)

7) Next steps

- What kind of competencies in multidisciplinary research do you feel like to have further improvement
  Probes:
  1) **personal knowledge and skills** in investigating a research question by integrating theories and methods of other disciplines into your primary discipline;
  2) **communicating** with researchers from other disciplines, for example describing your research perspective to them, disseminating your research among them; and
  3) **interacting** with researchers from other disciplines, such as writing a joint proposal, attending scholarly presentations in other disciplines.
- How will you work together with other researchers in the coming year for the phase-1 project? (divisions of responsibilities and joint efforts? Communication plan? Interaction?)
- with non-academic stakeholders?

8) Any other things you would like to add?
