# Supplementary material for: Identifying actions to foster cross-disciplinary global health research: a mixed-methods qualitative case study of the IMPALA programme on lung health and tuberculosis in Africa
Source: BMJ Open. 2022 Mar 29;12(3):e058126. doi: 10.1136/bmjopen-2021-058126 (PMC8966532; doi:10.1136/bmjopen-2021-058126)
Supplement: Supplementary data [file bmjopen-2021-058126supp005.pdf]

Version 2, 18 May 2018

Multidisciplinary cross-cutting capacity development project (MUDI)

Participant Information Leaflet

-For interviews-

My name is Yan Ding and I work for Capacity Research Unit, Liverpool School of Tropical Medicine. We would like to invite you for an interview in our research, the MUDI project. Before you decide, we would like you to understand why the research is being done and what it would involve for you. ***I will go through the information sheet with you and answer any questions you have.*** *This should take about 5 minutes. Ask me if there is anything that is not clear.*

What's the purpose of the interviews?

The MUDI project aims to generate robust evidence about what works for fostering research that involves multiple disciplines, and in what contexts, taking lung health and TB as a pathfinder. Also, it aims to develop evidence-informed and transferable recommendations for actions (and possibly a benchmark) that can catalyze effective and sustainable collaborative research of multiple disciplines in low and middle-income countries.

We conduct interviews besides other methods such as a survey to generate data to achieve the MUDI project's aims.

What is the MUDI project?

MUDI is a multidisciplinary capacity development research project under the umbrella of IMPALA which stands for the International Multidisciplinary Programme to Address Lung Health and TB in Africa. IMPALA is a four-year collaborative programme funded by the National Institute for Health Research in the UK.

Why have you been invited?

MUDI takes two multidisciplinary applied research projects in IMPALA as case studies, and they are: 1) Clinical and Socioeconomic determinants of lung function among young infants in Uganda: a birth cohort study; and 2) an integrated health systems approach for improving health services for chronic lung disease in Sudan and Tanzania.

You have at least one of the following roles: 1) a member of the External Scientific Advisory Board of IMPALA; 2) a director or co-director of IMPALA; 3) a researcher of at least one of the two abovementioned multidisciplinary applied research projects in IMPALA; and 4) a member of the project administrative team. Your experience and opinion in fostering research that involve multiple disciplines would be helpful for MUDI to identify barriers and facilitators of research with multiple disciplines, and alto to develop evidence informed recommendations in promoting effective and sustainable multidisciplinary research.

Do you have to take part?

It is up to you to decide whether to join the study. We will describe the study and go through this information sheet. If you agree to take part, we will then ask you to sign a consent form. You are free to withdraw at any time, without giving a reason. This would not affect the standard of care you receive.

What will happen to you if you take part?

Each interview will last between half an hour to one hour, and altogether you may be invited for such an interview with a maximum of 4 times in the following 3 years.

Ding Y, *et al. BMJ Open* 2022; 12:e058126. doi: 10.1136/bmjopen-2021-058126

Questions during the interview are related to your previous experience in multi-/inter-disciplinary research, changes and interactions of collaboration among different disciplines in IMPALA and its two-abovementioned multidisciplinary projects or regarding to barriers and facilitators of multidisciplinary research. You may feel discomfort to answer certain questions and you can choose not to answer at any time.

Compensation

You will not be paid for being part of this project.

What are the possible benefits of taking part?

The interview will yield valuable information on the interaction of multiple disciplines in IMPALA, on facilitators and barriers of the two above mentioned multidisciplinary research in IMPALA. It makes it possible to share emerging lessons across IMPALA so that improvements to the programme can be made thus enhancing the effectiveness and value for money of the IMPALA programme, also beyond IMPALA.

What will happen to any data you give and to the results of the research study?

All information you provide through the interview will be kept confidential and only shared among Capacity Research Unit researchers directly involved in MUDI project for research purpose. The findings will be reported without identifiable individual information.

What will happen if you don't want to carry on with the study?

You can withdraw from the study at any time, and you do not need provide a reason. If you withdraw from the study, nothing will happen to you.

Will you participate in the interview? Yes or No

If yes, I will provide you a consent form and please sign on it.

If you decide to participant in the interview, may I do an audio-recording of the interview? Yes or No

We would like to have the interview audio-recorded to check all points are correctly captured in the interview. we will do so only with your permission.

Contact Details

For further details, or if you have any questions or want to file a complaint about the research you may contact:

Organisation responsible for the study:

Dr. Yan Ding

Liverpool School of Tropical Medicine, UK.

E-mail: yan.ding@lstmed.ac.uk.

The LSTM Research Ethics Committee

E-mail: lstmrec@lstmed.ac.uk

Ding Y, *et al. BMJ Open* 2022; 12:e058126. doi: 10.1136/bmjopen-2021-058126
