# Supplementary material for: Identifying actions to foster cross-disciplinary global health research: a mixed-methods qualitative case study of the IMPALA programme on lung health and tuberculosis in Africa
Source: BMJ Open. 2022 Mar 29;12(3):e058126. doi: 10.1136/bmjopen-2021-058126 (PMC8966532; doi:10.1136/bmjopen-2021-058126)
Supplement: Supplementary data [file bmjopen-2021-058126supp006.pdf]

Consent Form

CONFIDENTIAL

**Study Title: Multidisciplinary cross-cutting capacity development project (MUDI)**	
**Principal Investigator: Professor Imelda Bates**	**Study Site: Sudan, Tanzania, Uganda. (for IMPALA's phase 1 non-embedded projects)**

	**Please initial box**
1. I confirm I have read and understood the information sheet dated 18 March 2018 (Version 1) for the above study. I have had the opportunity to consider the information, ask questions and have had these answered satisfactorily.	
2. I understand that participation in this study is voluntary and I am free to withdraw consent at any time, without giving a reason, without any penalties.	
3. I understand that data collected during the study, will be kept confidential and only shared among the Centre for Capacity Research researchers directly involved in the MUDI project for research purposes. The findings will be reported without identifiable individual information.	
4. I hereby declare that I have not been subjected to any form of coercion in giving this consent.	
5. I agree to take part in this study.	

Signing this declaration does not affect your right to decline to take part in any future study.

Name of participant	Date	Signature
Name of person taking Consent	Date	Signature

When complete: 1 copy for participant; 1 copy (original) for research

Ding Y, *et al. BMJ Open* 2022; 12:e058126. doi: 10.1136/bmjopen-2021-058126
