# Supplementary material for: Identifying actions to foster cross-disciplinary global health research: a mixed-methods qualitative case study of the IMPALA programme on lung health and tuberculosis in Africa
Source: BMJ Open. 2022 Mar 29;12(3):e058126. doi: 10.1136/bmjopen-2021-058126 (PMC8966532; doi:10.1136/bmjopen-2021-058126)
Supplement: Supplementary data [file bmjopen-2021-058126supp007.pdf]

IMPALA MUDI Meeting Observation Form

Date	
Location	
Title/topic(s)	
Presenter(s)	
Attendees (PIs and core members)	
Attendees (Dept./School representatives)	
Attendees (from wider collaborative organizations)	
Attendees (Others)	
Total Attendance	
Observer	
Overall Tone	
Overall Energy	
To what extend did synergy occur at this meeting?	A. Not at all (=1) B. 2 C. 3 D. 4 E. Very much (=5) F. Not sure

**Focus**	**Transdisciplinary Components**				**Tone Scale**	**Energy Scale**
1 Administrative<br>2 Indiv./group presentation<br>3 Scientific discussion<br>4 Open discussion<br>5 Post Meeting/Side Bar<br>6 Breaks<br>7 Other	A= Cross-disciplinary synergy<br>Such as:<br>• Brainstorming crossing analytical levels<br>• Integration of disciplinary ideas	B=Proposed or actual cross-disciplinary outcomes<br>Such as:<br>• Progress towards cross-disciplinary model<br>• Discussions regarding new course<br>• Intention stated to meet further	C=Info/Support<br>Such as:<br>• Infor/data presented (not cross-dis)<br>• Material/Technical support (actual or offered)<br>• Socio-emotional support<br>• Humour/Mood enhancement<br>• Movement towards consensus	D=Setbacks<br>Such as:<br>• Critical statements<br>• Interrupting others<br>• Distracting events (equip trouble, unexpected noise)<br>• Movement towards dissensus	+2=Very harmonious<br>+1=Harmonious<br>0=Neutral<br>-1=Conflicted<br>-2=Very conflicted	5=Very high<br>4=High<br>3=Medium<br>2=Low<br>1=Very low

Time (start, end and sum min)	Focus (1-7)	Qualitative Description and Details	Component (A-D)	Tone (-2 to +2)	Energy (1 to 5)

Time (start, end and sum min)	Focus (1-7)	Qualitative Description and Details	Component (A-D)	Tone (-2 to +2)	Energy (1 to 5)

Note: This observation form took reference from: Fuqua, J., et al., Transdisciplinary collaboration as a basis for enhancing the science and prevention of substance use and “abuse”. Substance use & misuse, 2004. 39(10-12): p. 1457-1514
