# Supplementary material for: Identifying actions to foster cross-disciplinary global health research: a mixed-methods qualitative case study of the IMPALA programme on lung health and tuberculosis in Africa
Source: BMJ Open. 2022 Mar 29;12(3):e058126. doi: 10.1136/bmjopen-2021-058126 (PMC8966532; doi:10.1136/bmjopen-2021-058126)
Supplement: Author's manuscript [file bmjopen-2021-058126.draft_revisions.pdf]

BMJ Open is committed to open peer review. As part of this commitment we make the peer review history of every article we publish publicly available.

When an article is published we post the peer reviewers' comments and the authors' responses online. We also post the versions of the paper that were used during peer review. These are the versions that the peer review comments apply to.

The versions of the paper that follow are the versions that were submitted during the peer review process. They are not the versions of record or the final published versions. They should not be cited or distributed as the published version of this manuscript.

BMJ Open is an open access journal and the full, final, typeset and author-corrected version of record of the manuscript is available on our site with no access controls, subscription charges or pay-per-view fees (http://bmjopen.bmj.com).

If you have any questions on BMJ Open's open peer review process please email info.bmjopen@bmj.com

BMJ Open

Actions for fostering cross-disciplinary global health research: A qualitative research

Journal:	*BMJ Open*
Manuscript ID	bmjopen-2021-058126
Article Type:	Original research
Date Submitted by the Author:	10-Oct-2021
Complete List of Authors:	Ding, Yan; Liverpool School of Tropical Medicine, Centre for Capacity Research<br>Tomeny, Ewan; Liverpool School of Tropical Medicine, Clinical Sciences<br>Bates, Imelda; Liverpool School of Tropical Medicine, Centre for Capacity Research<br>on behalf of, The IMPALA Consortium; IMPALA
Keywords:	PUBLIC HEALTH, QUALITATIVE RESEARCH, Anthropology < TROPICAL MEDICINE

SCHOLARONE™
Manuscripts

I, the Submitting Author has the right to grant and does grant on behalf of all authors of the Work (as defined in the below author licence), an exclusive licence and/or a non-exclusive licence for contributions from authors who are: i) UK Crown employees; ii) where BMJ has agreed a CC-BY licence shall apply, and/or iii) in accordance with the terms applicable for US Federal Government officers or employees acting as part of their official duties; on a worldwide, perpetual, irrevocable, royalty-free basis to BMJ Publishing Group Ltd ("BMJ") its licensees and where the relevant Journal is co-owned by BMJ to the co-owners of the Journal, to publish the Work in this journal and any other BMJ products and to exploit all rights, as set out in our licence.

The Submitting Author accepts and understands that any supply made under these terms is made by BMJ to the Submitting Author unless you are acting as an employee on behalf of your employer or a postgraduate student of an affiliated institution which is paying any applicable article publishing charge ("APC") for Open Access articles. Where the Submitting Author wishes to make the Work available on an Open Access basis (and intends to pay the relevant APC), the terms of reuse of such Open Access shall be governed by a Creative Commons licence – details of these licences and which Creative Commons licence will apply to this Work are set out in our licence referred to above.

Other than as permitted in any relevant BMJ Author's Self Archiving Policies, I confirm this Work has not been accepted for publication elsewhere, is not being considered for publication elsewhere and does not duplicate material already published. I confirm all authors consent to publication of this Work and authorise the granting of this licence.

For peer review only

Actions for fostering cross-disciplinary global health research

A qualitative research

Yan Ding, Ewan M Tomeny, Imelda Bates, and on behalf of the IMPALA Consortium

Yan Ding[1]*Centre for Capacity Research, Liverpool School of Tropical Medicine, Liverpool, L3 5QA, UK; Yan.Ding@lstmed.ac.uk

Ewan M Tomeny[2], Department of Clinical Sciences, Liverpool School of Tropical Medicine, Liverpool, L3 5QA, UK; Ewan.Tomeny@lstmed.ac.uk

Imelda Bates[1], Centre for Capacity Research, Liverpool School of Tropical Medicine, Liverpool, L3 5QA, UK; Imelda.Bates@lstmed.ac.uk

[1] Centre for Capacity Research, Liverpool School of Tropical Medicine, Pembroke Place, Liverpool, L3 5QA, UK

[2] Department of Clinical Sciences, Liverpool School of Tropical Medicine, Liverpool, L3 5QA, UK

*Corresponding author. Centre for Capacity Research, Liverpool School of Tropical Medicine, Pembroke Place, Liverpool, L3 5QA, UK
E-mail address: Yan.Ding@lstmed.ac.uk (Yan Ding)
Tel: (+44) (0)1517029316

Word count: 4776

For peer review only - http://bmjopen.bmj.com/site/about/guidelines.xhtml

Actions for fostering cross-disciplinary global health research: A qualitative research

Abstract

Objectives: To identify actions for fostering cross-disciplinary research skills and collaborations in global health, and to produce recommendations for improving the design, implementation, and management of cross-disciplinary global health research programmes.

Design: Using a North-South global health research programme as a case study — and following an adapted framework — we conducted qualitative research using document reviews, semi-structured interviews (purposive sampling), and participatory observation. We used baseline-survey findings to identify potential interviewees and tailor interview guides.

Setting: Our case study was a 4.5-year (2017-2021) programme, namely *the International Multidisciplinary Programme to Address Lung Health and Tuberculosis in Africa* (IMPALA). Led by a UK research institute, IMPALA spanned 22 partner organisations from 13 countries (10 in sub-Saharan Africa), and involved five research discipline groups*: clinical science*, *social science, health systems*, *health economics*, and *policy/research capacity*.

Participants: Thirty-one IMPALA members were interviewed (Jul.2018-Nov.2019), with interviewees evenly split by sex (16F; 15M) and by Global North/South institution (15 UK; 16 Africa). Twenty-five (81%) were researchers, comprising 18 senior researchers (professors, readers, associate professors, senior lecturers) and 7 early career researchers (assistant professors, lecturers, research fellows, post-docs, research assistants, PhD students). Twenty-four programme events were observed (Sept.2018–Apr.2020) and 49 documents were reviewed (Dec.2017–Apr.2020). All 66 IMPALA staff were sent the baseline survey, receiving 51 responses (43/56 researchers; 8/10 non-researchers).

Results: Ten themes emerged, which suggested that cross-disciplinary research — while valued by many — is not universally understood, and the time it requires often underestimated. We found that fostering cross-disciplinary research and managing tensions needs planning and continuous discussions and interactions. A shared vision with explicitly agreed goals and roles, and active management of cross-disciplinary activities is essential.

Conclusions: Active planning, implementation, and management of cross-disciplinary activities are essential for the success of cross-disciplinary global health research, and should be separate from the primary research activities.

For peer review only - http://bmjopen.bmj.com/site/about/guidelines.xhtml

Strengths and limitations of this study

- We used an adapted published framework and a recent literature review to frame our data collection tools and analysis, and have placed our findings in the context of current global knowledge concerning cross-disciplinary research.
- The credibility of our findings is strengthened from having used interview and observational data from diverse interviewees and events, corroborated by document analysis.
- Our study focused on a single cross-disciplinary global health research programme and its projects.
- We have enhanced the transferability of our findings by describing the complexity of the programme and the context within which the cross-disciplinary research took place.
- Our role as IMPALA members in conducting research on cross-disciplinary working in IMPALA may affect interviewees' responses which we mitigated by ensuring confidentiality.

Main text

INTRODUCTION

Bringing together researchers from multiple disciplines can lead to innovation and rapid production and dissemination of cross-disciplinary knowledge to solve complex global health problems.[1, 2] Cross-disciplinary research has been growing globally in popularity among researchers and funders because of its importance in addressing global health challenges.[1, 2] 'Cross-disciplinary research' covers three typologies: multi-, inter-, and trans-disciplinary research. In this article we will use the term cross-disciplinary research (CDR) to mean research that combines concepts, methods, and theories drawn from two or more disciplines.[3]

Existing evidence on fostering CDR is fragmented across disciplines,[4, 5] making it difficult to find. There is increasing interest in understanding how to implement effective CDR and in the importance

For peer review only - http://bmjopen.bmj.com/site/about/guidelines.xhtml

of team dynamics between researchers from disparate disciplines. CDR tends to be more complex than traditional types of research,[6] and presents unique challenges,[1, 3] such as problem definition, positioning in different disciplines[7] and coordination of effort.[8, 9] Our previous literature review found that evidence about how to conduct effective CDR is primarily from high-income countries, and may not apply to CDR in global health, where research is typically conducted through North-South collaborations.[3]

We used the *International Multidisciplinary Programme to Address Lung Health and Tuberculosis in Africa* (IMPALA 2017-21))[10] as a case study to explore and reflect on practical actions for fostering CDR in North-South collaborations. IMPALA aimed to generate knowledge and implementable solutions concerning lung health and tuberculosis. Led by a Global North research institute, IMPALA had 22 international partner organisations from 13 countries, 10 in sub-Saharan Africa.

IMPALA explicitly used multidisciplinary approaches and spanned biology to policy[11]. It involved five research disciplines: *clinical science*, *social sciences*, *health systems*, *health economics*, and *policy/research capacity*. Unusually, to promote fairness and overcome disciplinary hierarchies, the programme was framed around these discipline groups: each group initially received the same amount of funding and was represented on the management team alongside the three consortium directors. Each group had one PhD student and one postdoctoral researcher (Figure 1), with equal training opportunities offered to all early career researchers (ECRs).

[Insert Figure 1: The IMPALA Organogram]

Our study has drawn on IMPALA as a whole, and its two embedded projects (hereafter '*the two projects*'): one combined *clinical sciences* and *health economics*; the other *health systems* and *social sciences*. Our study explores the 'real life' actions taken to foster CDR across IMPALA and based upon our findings we recommend actions to improve the effectiveness of future global health CDR programmes.

For peer review only - http://bmjopen.bmj.com/site/about/guidelines.xhtml

METHODS

We adapted a four-phase model of CDR collaborations (Figure 2)[9] which describes objectives within each project phase (i.e., *Development*, *Conceptualisation*, *Implementation*, *Translation*). We combined the *Development* and *Conceptualisation* phases into one 'Planning' phase, since global health research activities in these phases are generally integrated.[12] The *Translation* phase was not included because it requires long-term follow-up. Our literature review indicated that leadership and management strongly influence CDR effectiveness,[3] so these were added as a cross-cutting framework component.

[Insert Figure 2: The three-component framework for the cross disciplinary collaborative research process used in this study (adapted from Hall et al., 2012)]

Data collection

Our primary source of data was semi-structured interviews, supplemented by a baseline survey, a document review, and observations of events.

Baseline survey

All IMPALA members were invited to complete a baseline survey (May-September 2018). This included individuals from the External Scientific Advisory Panel, Leadership and Management Teams, Administrators, and researchers/policy makers involved in the two projects. The survey collected participants' personal information, and their experience of, and confidence in, conducting CDR. The survey findings were used to identify potential interviewees and tailor interview guides.

Semi-structured interviews

Sample selection

For peer review only - http://bmjopen.bmj.com/site/about/guidelines.xhtml

The interviews collected data on challenges and practical actions/solutions related to the fostering and conducting CDR. Guided by our baseline survey data 31 primary interviewees were selected using the IMPALA team directory as a sampling frame. Purposive sampling was used to maximise variation in roles, disciplinary backgrounds, career stages, gender, affiliated organisations, and geographical locations[13].

Procedure of interviews

Interview questions were based on the adapted framework (Figure 2) with probes informed by our literature review.[3] Interviews were audio recorded and conducted in English, either in-person or virtually. Participants received an information sheet prior to their interview and provided informed consent. For anonymity each interviewee was assigned an ID.

<u>Document review</u>

Data were extracted from documents concerning the programme's: *vision*, *goals*, *research questions*, *design*, *teams*, *interactions*, and *outputs,* to understand the context of the programme and its projects, inform interview questions, and cross-check findings from other data-collection methods. Documents included the IMPALA website, concept notes, proposals, minutes/agendas from annual meetings, and quarterly research updates.

<u>Observation of events</u>

YD was a participant observer at events involving cross-disciplinary issues including two annual meetings, monthly knowledge exchange meetings, training workshops, and a 4-day field visit to Tanzania. Observation notes were entered in real time into a pre-designed form informed by the literature,[14] comprising sections on: brainstorming the crossing of analytical levels; integration of disciplinary ideas; proposed/actual cross-disciplinary outcomes; information sharing; technical or emotional support; and challenges and setbacks.[14] Observation findings were used for refining interview questions and triangulating interview data.

For peer review only - http://bmjopen.bmj.com/site/about/guidelines.xhtml

Data analysis

Interview data were coded, mapped and analysed using the framework (Figure 2). Narrative summaries were created using thematic synthesis.[15] Findings were triangulated using the document review and observations.

Patient and public involvement

While the IMPALA programme involved both patients and the public, due to this study's specific focus on research practice, its design, conduct, and reporting did not involve patients or the public.

RESULTS

Interviewee characteristics

Thirty-six interviews with 31 interviewees were conducted each lasting 68-192 minutes. Five individuals were interviewed again after one year to identify changes in CDR in the two projects. 52% of interviewees (16/31) were female, and 16 were based at African organisations from 7/10 partner African countries. Twenty-five (81%) were researchers, comprising 18 senior researchers (i.e., professors, readers, associate professors, senior lecturers) and 7 ECRs (i.e., assistant professors, lecturers, research fellows, post-docs, research assistants, PhD students) (Table 1).

Table 1: Interviewees' characteristics

	**Items**	**Option**	**N**
1	Role in IMPALA (Options not mutually exclusive)	A member of the External Scientific Advisory Panel	2
		A member of the Leadership Team	4
		A member of the Management Team	8

For peer review only - http://bmjopen.bmj.com/site/about/guidelines.xhtml

	Items	Option	N
		A member of the Management & Administration Support Team	3
		Other member working across IMPALA projects	2
		A researcher or policy maker on the two projects;	15
		(of these those based in Africa)	(8)
		A member who was based in Africa, working on IMPALA, but not on the two projects	5
2	Sex	Female	16
		Male	15
3	Location	African Countries	16
		United Kingdom	15
4	Primary Disciplinary Background	Medicine & Clinical Sciences	18
		Humanities & Social Sciences	10
		Others	3
5	Profession	Researchers/research leaders	25
		Non-research members	6
6	Academic Rank (of the 25 researchers)	Senior researcher	18
		Early career researcher	7

Survey, document reviews and observations

The baseline survey was sent to 66 IMPALA staff, with responses received from 43/56 researchers (77%), and 8/10 non-researchers (80%). Twenty-four events were observed over 20 months (Sept. 18 – Apr. 20) and 49 documents were reviewed (Box 1).

For peer review only - http://bmjopen.bmj.com/site/about/guidelines.xhtml

Box 1: Internal IMPALA documents used to provide background information for this study

1. The IMPALA website: https://www.lstmed.ac.uk/impala
2. IMPALA Technical Proposal
3. IMPALA Team Directory
4. The concept notes of all the eight research projects sitting under IMPALA
5. IMPALA Publication Guidelines
6. IMPALA Data sharing, Access and Release Policy
7. IMPALA Data Management Guidelines
8. IMPALA Communications Plan
9. IMPALA kick-off meeting in 2017, annual meetings in 2018 and 2019, including
    1) Meeting schedule
    2) Attendees list and biographies
    3) Meeting slides
10. IMPALA technical reports, including
    1) IMPALA 2017 report (covering the first six months of IMPALA)
    2) IMPALA 2018 annual technical report
    3) IMPALA 2019 annual technical report
11. Research ethics application documents of the two case study projects, including research proposals and data collection tools
12. Quarterly updates by the four post-doctoral researchers each on the two post-doctoral researchers led projects (August 2018 - December 2019, 20 documented updates in total)
13. IMPALA Year 1-3 Joint Outputs list

For peer review only - http://bmjopen.bmj.com/site/about/guidelines.xhtml

Research results

Ten themes (7 themes concerning the *Planning and Implementation* Phases; 3 concerning *Leadership and Management*) emerged from the findings. Interviewee's anonymised quotes are presented with their main role (researcher/non-researcher) and location (Africa/non-Africa).

Actions that fostered CDR in the Planning Phase

Shared vision and goals

Interviewees identified that co-development of the IMPALA proposal between members from the Global South and North helped them reach a common vision. While this was time-consuming due to the large number of cross-disciplinary, inter-organisational, and geographically distanced members, several factors helped the process including the existence of previous/ongoing collaborations and involvement in professional associations.

During the face-to-face start-up meeting, IMPALA members and the 22 participating institutions introduced themselves and IMPALA's vision and strategic objectives were discussed. Specific goals for projects —and for IMPALA as a whole— had purposefully been left undefined by the management team so they could be co-developed during this meeting. Interviewees reported finding this meeting useful for grasping the 'bigger picture' of IMPALA, and for learning about one other.

Expectations of programme-level goals and success

Interviewees had different expectations of IMPALA depending on their seniority and disciplinary background. Senior, clinical researchers tended to focus on the need to expand collaborations with partners. Two interviewees suggested that since many senior researchers had clinical sciences backgrounds, IMPALA provided more opportunities for clinical researchers to expand collaborations, compared to other programmes. Interviewees from non-clinical disciplines (e.g., social sciences and health systems) were more focused on their existing projects and research quality. Senior researchers

For peer review only - http://bmjopen.bmj.com/site/about/guidelines.xhtml

sought to enhance ECR's research skills, and ECRs were focussed on generating outputs and building working relationships. Non-researchers focussed on programme delivery, and capacity strengthening in areas such as financial management, leadership, and policy engagement. All interviewees reported expecting IMPALA to lead to new research questions and new funding. Observation data confirmed all these findings.

Interviewees recognised the complexity of aligning project and programme goals. Two interviewees acknowledged the difficulty of collective prioritisation, and proposed mapping the connections between programme and project objectives, possibly annually. One participant stated that although seeking clarity around programme goals can facilitate members' engagement, balancing partnership development against addressing a large-scale broad research question with multiple disciplines is difficult.

Shared understanding of research questions and activities at the project level

IMPALA's proposal outlined broad topics for research projects —with named leads and partners for each— while leaving specific research questions and activities to be developed during the start-up meeting. Project leads recognised that this allowed research questions to be based on the interests and experience of partners, and some expressed appreciation that programme leaders had not imposed personal priorities.

Researchers from both Global North and South were comfortable with this process, with Global South partners feeling they had driven the research agenda:

> *"I was looking at ways how I can also contribute rather than just passively engage in national meetings … we were there to conceptualise...what we want to do, … we got the research budget." (ID-21, researcher, Africa)*

Others noted a risk of mismatch between programme and project goals, and had difficulty narrowing research questions down from programme to project level:

For peer review only - http://bmjopen.bmj.com/site/about/guidelines.xhtml

"When you have .... multiple perspectives that lead to such a broad potential for research questions that narrowing down and getting in some consensus can be quite difficult." (ID-1, researcher, Africa)

The two project teams addressed this differently:

One developed research questions based on a baseline assessment conducted during joint field trips with local research and implementation teams, enabling them to develop locally important, high-priority research questions. To address these questions, they drew on methods from their two core disciplines, indicating some complementary in their disciplinary paradigms such as theories, research methods, and standards. The benefits of having one project integrating two disciplinary components appeared clear to this team from the outset.

The other project team initially generated their research questions independently within each of their two disciplines and then merged the projects through discussions and negotiation which was "*initially uncomfortable" (ID-13, researcher, non-Africa).* One researcher believed "*practical efficiency in terms of time and data collection*" *(ID-9, researcher, Africa)* of this approach to have been the main advantage of merging the two disciplinary research projects into one.

Reasons for using CDR in IMPALA

IMPALA took a CDR approach as it was felt its broad research question – i.e. *to address lung health and tuberculosis in Africa* – required inputs from multiple disciplines and programme leaders recognised that everyone had a role in ensuring research findings informed policy. Interviewees considered CDR as one of the "*most effective ways to generate the best possible outputs and outcomes" (ID-13, researcher, non-Africa)* since it "*enables appropriate generalisation of research outcomes*" *(ID-15, researcher, non-Africa)*. Several interviewees mentioned that multidisciplinary research was a funder's requirement; however one cautioned *"don't just do* [CDR] *for the sake of it" (ID-14, researcher, non-Africa)*.

For peer review only - http://bmjopen.bmj.com/site/about/guidelines.xhtml

While most senior researchers recognised the importance of CDR, most interviewees (researchers and non-researchers) had not participated in explicit discussions on what actions would be needed to conduct CDR.

> *"A lot of the challenges is people are so busy doing their own things that they forget that that is what needs to happen." (ID-12, researcher, non-Africa)*

The IMPALA programme included a post-doctoral researcher (YD) dedicated to investigating cross-disciplinary working. The definitions of multi-, inter- and cross-disciplinary research were presented to IMPALA members during the second IMPALA annual meeting prompting discussions and clarifications. However, interview findings suggest such clarifications would have been useful earlier, alongside discussions on pre-specified goals/methodologies concerning cross-disciplinary working.

Cross-disciplinary orientation

Observations clearly indicated that IMPALA members valued understanding more about each other and their disciplines especially within a group environment of psychological safety, while highlighting the value of clarifying disciplinary boundaries to prevent conflicts.

Having inputs from colleagues with various disciplinary backgrounds at the planning phase and arranging formal time for candid conversations on research questions and design were viewed by interviewees as critical. A programme leader and a researcher highlighted potential tensions in cross-disciplinary working, and the need for maintaining *'discipline uniqueness'*. The process of defining and clarifying research goals among disciplines was considered to have helped clarify disciplinary boundaries:

> *"After the goals are fixed and then each goal somehow belongs to certain disciplines...relate data to that goal and then deal with the data, publication, all those things followed." (ID-15, researcher, non-Africa)*

For peer review only - http://bmjopen.bmj.com/site/about/guidelines.xhtml

Actions that fostered CDR in the Implementation Phase

Shared understanding of roles and responsibilities

Collaborative working was facilitated by a shared understanding of the roles and contributions of different disciplines and partners, along with an appreciation that successful cross-disciplinary collaborations require complementarity rather than competition. This helped team members to overcome *'fighting for space'* and *'struggling for context leadership'* (ID-22, researcher, Africa). Several interviewees noted the importance of research administrators in helping to understand responsibilities.

> *"Because we [administrators] are that sort of hub in the middle, and we do oversee everything. We can sort of speak on behalf of the project and say that this isn't working and have a bit of input in that way." (ID-3, non-researcher, non-Africa)*

Several interviewees had not had open discussions about roles and responsibilities, with one suggesting that roles were defined by one's job description and another explaining that *"as a member of the team you naturally know your strengths and therefore role" (ID-5, researcher, Africa).* Another interviewee highlighted that assumptions regarding roles and responsibilities had the potential to cause confusion and needed open discussions:

> *"I increasingly think the best way to have good, harmonious, collaborative relationships is to be really upfront about roles and responsibilities. To do that first so that there is no confusion after." (ID-9, researcher, Africa)*

One interviewee suggested that jointly developing a work plan, containing explanations of responsibilities alongside a clear timeline could help to clarify roles.

Reconciling individual expectations while navigating different contexts

For peer review only - http://bmjopen.bmj.com/site/about/guidelines.xhtml

Several interviewees advocated for open discussions on roles, suggesting such discussions were important because people were at different career stages with different experiences, cultures and academic systems, which could cause mismatched expectations of one another's roles. This led to, for example, disagreements on the time spent in the research sites and responsibilities for research coordination. Clarifying roles and having a host country/institution coordinator was thought to be essential in avoiding these issues.

Regular cross-disciplinary project update meetings along with individual conversations to provide performance feedback to ECRs (including those with different disciplinary backgrounds) were said to be useful by both ECRs and senior researchers. Role modelling was also identified as important in encouraging ECRs to continuously explore other disciplines:

> *"Seniors and line managers say, 'You should go to this. Think about this...' So, it does need people, at a senior level, to think broadly and encourage that." (ID-23, researcher, non-Africa)*

Support across disciplines was valued during project implementation, for example when developing questionnaires, collecting and analysing data and several senior researchers called for more thought on how to provide supportive supervision:

> *"Perhaps we didn't think hard enough about how to support the projects and who should be supporting the projects and in what way." (ID-9, researcher, Africa)*

Team learning

The importance of individuals' ability to blend disciplinary edges was raised by an interviewee and many others shared their approaches to understanding other disciplines. Senior researchers also encouraged colleagues to consider broadening the scope of their work and skillset through formal cross-disciplinary training, mutual learning, and joint supervision in other subject areas. One month after the interviews, monthly knowledge exchange meetings were initiated to improve cross-disciplinary learning and communication.

For peer review only - http://bmjopen.bmj.com/site/about/guidelines.xhtml

For peer review only

Leadership and Management

Communication planning and implementation

New IMPALA members appreciated their one-to-one induction meetings with key researchers and administrators. Joint site visits by members from the Global North and South were helpful in forming relationships and in promoting cross-fertilisation. Face-to-face meetings were valued for facilitating the design, prioritisation, and development of both research projects and teams, especially concerning developing methods and budgeting. Interviewees said that virtual meetings and email communications worked well and were useful, though several raised issues with internet connections. Effective planning to maximise the availability of team members was highlighted:

> *"What I usually do is to inform them early enough because they have lots of responsibilities…After they have considered then you block the time... With multi-disciplinary, it needs proper planning, especially on timing." (ID-26, researcher, Africa)*

Many senior researchers often had long working relationships with country partners. To help ECRs to build mutual understanding and develop research networks, regional meetings for ECRs across disciplines were suggested.

Several interviewees suggested that having access to other teams' materials and outputs could have improved cross-disciplinary understanding. A common platform for document and information sharing was subsequently established. Interviewees further proposed that cross-disciplinary communications should be expanded. Accordingly, the monthly knowledge exchange meetings were expanded beyond ECRs to include administrative staff, in-country partners, and researchers beyond IMPALA's core team.

Interviewees wanted more time to develop mutual understanding in CDR and to create a sense of ownership. One interviewee reflected *"we need to have some more recognition of the need for time*

For peer review only - http://bmjopen.bmj.com/site/about/guidelines.xhtml

for some of the processes and the collaborations to work for the future" (ID-11, researcher, non-Africa). Another recommended taking time to learn about each other's experiences and expectations, ways to successfully collaborate, and for joint preparation of project tools (e.g. databases).

According to several interviewees *"there are inevitable delays in starting" (ID-9, researcher, Africa) for example* in funding release (6 months), international staff recruitment (5-8 months) and ethics approval (7-8 months). Interviewees described how they felt the need to focus on outputs although *"would have loved to have used those six months to think about how we prepare these disciplines to work together" (ID-11, researcher, non-Africa).* One interviewee highlighted the importance of prioritising internal communication even within tight timescales, arguing *"sometimes prioritising a two-hour meeting to make sure everyone's on the same page and understanding things in the same way is equally important as papers and research outputs" (ID-11, researcher, non-Africa).*

Nurturing trust and a group environment of psychological safety

Two senior researchers, three ECRs and three non-researchers noted that IMPALA management had helpfully promoted involvement and empowerment of ECRs and non-researchers and two ECRs appreciated the space and freedom their line managers had given them to lead projects.

There were three other suggestions offered by interviewees for nurturing trust:

i) Treating everyone equally through *"flat management"*:

> *"I very strongly believe in flat management, a structure everybody is equal. If I have a research meeting in my team, they all know we are equal. If they have something to say, they are all happy to say, and confident to say it." (ID-2, researcher, non-Africa)*

ii) Building trust by delivering on commitments (mentioned by two researchers and one non-researcher):

For peer review only - http://bmjopen.bmj.com/site/about/guidelines.xhtml

"To build trust you need to deliver... I think that's important, showing that you want to do your best. Then by reflection they don't want to let me down, so they deliver, and that's how you build trust, I think." (ID-4, non-researcher, non-Africa)

iii) Being transparent and learning from mistakes.

"Transparent, I think building trust… Also within trust and team, you have to allow mistakes… Accepting and also sitting together and see how we can handle it next time." (ID-21, researcher, Africa)

Addressing disciplinary hierarchies through the management structure

According to three interviewees, disciplinary hierarchies emerged when one discipline's work depended on another. For example, when one discipline's research questions and analysis relied on another's data generation, the latter may perceive their research activities should be prioritised over the former. Despite both projects having been allocated equivalent resources at the start of IMPALA, perceived imbalances arose. Five interviewees suggested that since clinical aspects were the primary interest of several IMPALA leaders, this may have inadvertently contributed to disciplinary hierarchies. Furthermore, several interviewees found the equal allocation of resources limiting, potentially hindering the effective answering of some research questions. Two interviewees further noted that since studies were highly interconnected at the operational level, strict drawing of financial boundaries between projects could at times *"lead to tensions" (ID-1, researcher, Africa).*

Following the initial equal allocation of resources, a degree of re-negotiation continued throughout IMPALA's lifetime though some members questioned the success of this process. One remarked that *"an alternative approach may be to develop the budget based on justified activities" (ID-15, researcher, non-Africa).*

Handling disciplinary differences and managing emotions

For peer review only - http://bmjopen.bmj.com/site/about/guidelines.xhtml

At times, the different approaches and priorities of disciplines led to some disagreements. Overall, the group which combined *Clinical Sciences* and *Health Economics* was perceived as predominantly outputs-driven whereas the *Humanities and Social Sciences group* appeared primarily focussed on processes, consultation, and discussions. One interviewee from the Management Team reflected *"we probably hadn't paid enough attention to the need for the process [of discussions between the management team members]"* because it "*requires sustained effort to balance the natural priority of an individual's discipline against that of multiple disciplines*" *(ID-11, researcher, non-Africa)*. Two interviewees suggested that time spent discussing managerial and logistics issues could have been more productively spent on research activities and constructive management of disciplinary disagreements.

Several interviewees described encountering emotional challenges, most frequently caused by disciplinary differences and some identified having needed for dedicated meetings to manage emotions in a professional environment. One interviewee commented that their previous working relationships and sense of responsibility had helped to make these conversations possible.

Such conversations resulted in real-time adaptations to the programme to enhance cross-disciplinary relationships. For example, monthly Directorate and Management Team meetings were merged and a rotational system for the management meeting chair was instigated whereby each discipline lead and the consortium directors took turns in chairing. Handovers between meetings were supported by the Programme Management and Administration support staff. Actions to promote more effective cross-disciplinary collaborations were also identified through a group exercise during the second annual meeting, and reviewing uptake of these actions became a standing item at management meetings. These changes were viewed as positive by several interviewees.

Developing research networks

For peer review only - http://bmjopen.bmj.com/site/about/guidelines.xhtml

Interviewees emphasised the programme's many good working relationships between different partners across Global North and South and noted the considerable benefit from strong previous relationships of key leaders. The importance of enabling the development of such research networks was a repeated theme from interviews.

> *"I think it [IMPALA] has really done a great job bringing great collaborators in terms of Africa with Liverpool, countries that are involved. I think it's really an interesting network and it has brought us together, many collaborators. People have never even met." (ID-21, researcher, Africa)*

Two interviewees reflected that project activities had helped build up trust and develop research networks.

> *"I hope my work […] will let them [current IMPALA members] say that 'he would actually put the neck on the line and physically help you. Get him on board.'" (ID-2, researcher, non-Africa)*

Strengthening capacity

Several approaches to capacity strengthening were identified through interviews and corroborated by internal documents. These included:

- Training workshops for those with different disciplinary backgrounds from the training subjects (e.g., training on social science research methodologies, policy engagement, statistics, and spirometry)
- Coaching through team meetings and one-to-one discussions (e.g., two interviewees emphasised that discussions with a statistician catalysed research)
- Mentoring ECRs and providing them with platforms at high-level international meetings (e.g., the UN General Assembly)
- Learning through peer support and reflection was mentioned by ECRs, senior researchers, and non-researchers:

For peer review only - http://bmjopen.bmj.com/site/about/guidelines.xhtml

For peer review only

> *"I feel like I'm definitely learning a lot… It's nice working so closely with […] and she's able to delegate things to me as and when they come up." (ID-3, non-researcher, non-Africa)*

Capacity strengthening also involved administration and field teams:

> *"My ideal world would be a world where everyone can do it because that's capacity building in-country. And it is not just the research, it's the admins." (ID-4, non-researcher, non-Africa)*

DISCUSSION

We adapted and expanded a published framework to underpin our research. Our findings emphasise that CDR programmes require careful planning, implementing, and managing and we have identified actions to promote CDR including some that have not previously been published.

Actions in programme planning to foster CDR

Clarity in defining 'Cross-disciplinary Research'

Similar to other studies we found a lack of agreement on defining multi-, inter- and trans-disciplinary research.[16] Our findings demonstrate that explicit discussions concerning both these definitions and what CDR means in practice are critical in the Planning Phase.

Managing expectations and harmonising goals

Participants had different expectations about being involved in CDR and highlighted the importance of negotiating a clear shared vision, taking into consideration individuals' expectations.[17] To harmonise goals, frequent discussions and interactions such as information sharing can be helpful, [3, 18] and need to be more frequent and intensive than in mono-discipline research.[9] Our findings shed light on tensions that can arise early in CDR, including balancing flexibility and acceptance that

For peer review only - http://bmjopen.bmj.com/site/about/guidelines.xhtml

For peer review only

not all aspects of the research could be initially ‘nailed down’, with developing a common understanding of the goals.

As with previous studies, IMPALA’s participants recognised the importance of a common conceptual framework for outlining the vision, objectives and organisational structure for showing the contributions of each discipline,[19] and to guide collaborations.[17, 20] Furthermore, evidence suggests that to have explicit knowledge integration goals for CDR is helpful.[20, 21] IMPALA’s conceptual framework was strengthened during the programme, for example by taking account of local contexts (achieved through joint field trips and discussions), by co-developing research questions and by drawing methods from relevant disciplines.

Actions in programme implementation to foster CDR

Our findings reflect previous studies which suggest that cross-disciplinary relationships flourish if they are prospectively planned and actively monitored.[3, 9] This is best managed separately from activities that focus on research outputs since fostering cross-disciplinary relationships requires its own planning and activities,[22] specific monitoring indicators and mechanisms for collecting data against the indicators.[23]

Management actions to foster CDR

Development of research collaborations and networks

Our study revealed important findings concerning management strategies for encouraging equitable partnerships, fostering CDR and reconciling individual expectations. These included involving northern and southern partners in co-developing a shared vision and goals, designing project-level research questions and activities, and strengthening capacity in line with a baseline capacity assessment.

For peer review only - http://bmjopen.bmj.com/site/about/guidelines.xhtml

Allowing time to promote cross-disciplinary activities

Our research also identified that researchers lacked sufficient time to successfully engage in discussions and processes to promote cross-disciplinary activities. Building in adequate time and funds for this throughout the programme is critical and may necessitate a shift in research planning as well as an understanding among research funders that such allocations are essential.

Lack of time for active consideration and management of activities to promote cross-disciplinary working is closely linked to lack of effective communication among programme members to bridge across disciplines.[24, 25] Interviewees proposed that cross-disciplinary communications should include all team members. This requires an agreed internal communication plan, administrative support and an electronic communication platform. Other studies have also highlighted the importance of an accessible space to document programme work and decision making.[26]

Programme adaptations to address hierarchies and tensions

Our framework specifically recognised 'nurturing trust and a group environment of psychological safety', 'communication planning and implementation', and 'team learning' in CDR as important because of possible emotional issues associated with ownership, territoriality, academic and discipline hierarchy, and disciplinary differences. Similar to previous studies, our findings identified CDR-related emotional issues (particularly around power and hierarchy) and disagreements in disciplinary approaches.[17, 27, 28] IMPALA took measure to mitigate such frictions, including providing equal funding and training opportunities, and by adjusting the programme's management structure. There included merging monthly Directorate and Management Team meetings, having a rotating Chair for Management Team meetings, initiating monthly knowledge exchange meetings for mutual learning and cross-disciplinary communications, and creating a common platform for document and information sharing. In addition, emerging findings from our study on CDR were presented at

For peer review only - http://bmjopen.bmj.com/site/about/guidelines.xhtml

Management Team meetings and summarised in quarterly bulletins for all IMPALA members so that they could inform subsequent programmes.

The three-component framework on CDR

Using our 'real-life' findings we adapted and expanded a published model of cross-disciplinary collaborative research processes[9] to create a framework useful for collecting and analysing multi-source and multi-perspective data on CDR in real-time. A new component of the framework emphasised the importance of leadership and management in CDR processes. We would recommend further adaptations to the framework to include a rationale for the components and to expand the 'shared understanding of who knows what, who does what, and how things get done'[9]. In addition to being useful for future research on CDR, our framework could be used to guide the design of cross-disciplinary programmes since it has practical applications across the three programme components of planning, implementation and management (Figure 2).

Recommendations

Based on the findings from our study, our adapted framework and our knowledge of the current literature, we have developed recommendations for planning and implementing future CDR in global health to improve the effectiveness of CDR processes from the outset (Table 2).

Table 2: Recommendations for the planning, implementation and management of cross-disciplinary global health research

**Research Phase**	**Recommendations**

For peer review only - http://bmjopen.bmj.com/site/about/guidelines.xhtml

Planning Phase	1) Allocate adequate time to develop a shared vision and goals, including<br>a. Co-designing of programme goals;<br>b. Aligning individuals' expectations and projects' aims with the programme-level goals;<br>c. Involving all partners in proposal development, maintaining flexibility, considering individual interests and disciplines;<br>d. Justifying and communicating the cross-disciplinary approaches to be adopted and reflecting cross-disciplinary processes in an action plan;<br>e. Developing and communicating a shared understanding of the roles, responsibilities and potential contribution of disciplines and partners.<br>2) Negotiate disciplinary boundaries when necessary;<br>3) Assess and strengthen in-country teams' capacity in cross-disciplinary research, and maintain clear plans for the involvement of in-country teams in decision making processes.
Implementation Phase	1) Jointly develop and pre-agree on internal approaches of working across disciplines, including communication, data access and management, publication policy and credit allocation;<br>2) Track the implementation of cross-disciplinary processes with pre-agreed indicators and review and respond accordingly.
Leadership and management	1) Rotate chairs for programme management meetings to ensure prominence of all relevant disciplines and with a process for handover and preparation between meetings.<br>2) Define and agree transparent programme-level mechanisms for strategic decision making;

For peer review only - http://bmjopen.bmj.com/site/about/guidelines.xhtml

	3) Develop a programme-level leadership and management plan to deliver the cross-disciplinary outputs and outcomes, including regular review of tracking indicators;<br>4) Agree roles and responsibilities, and accountabilities, and communicate these clearly to all programme members, making it explicit that every role is important in cross-disciplinary research (i.e. not just researchers).<br>5) Support an open culture of raising concerns and putting mechanisms in place for requesting support and responding to requests<br>6) Establish mechanisms for early identification of tensions, and for reflecting on and flexibly resolving differences and conflicts<br>7) Provide opportunities for joint learning and knowledge exchange across disciplinary boundaries especially methods and approaches (e.g., monthly knowledge exchange meetings)<br>8) Identify a platform for joint sharing and updating of documents.

Acknowledgements

We are grateful to all participants who agreed to take part in this case study and provided their valuable information and insight. We are also thankful to the IMPALA central administration team and the Centre for Capacity Research administration team, Annmarie Hand, Elly Wallis, Lorelei Silvester, Zena Parker who made the necessary logistical or administrative arrangements to allow a smooth data collection process. We would like to thank our Research Impact & Knowledge Translation Officer, Susie Crossman for her support in reviewing and editing the manuscript. We are grateful to Martina Savio, Dr Angela Obasi and Professor Stephen Bertel Squire from the IMPALA Management Team for their review of an earlier draft.

For peer review only - http://bmjopen.bmj.com/site/about/guidelines.xhtml

The original protocol for the study (supplementary file 1)

A funding statement

This research was funded by the National Institute for Health Research (NIHR) (project reference 16/136/35) using UK aid from the UK Government to support global health research. The views expressed in this publication are those of the author(s) and not necessarily those of the NIHR or the UK Department of Health and Social Care.

A competing interests statement

None declared.

Author Contributors

YD: Conceptualization, Methodology, Data Curation, Writing- Original draft preparation

EMT: Methodology, Writing- Reviewing and Editing.

IB: Conceptualization, Methodology, Writing- Reviewing and Editing, Supervision.

All authors read and approved the final manuscript.

A patient consent form

Not applicable.

Research ethics

Ethical approval was provided by Liverpool School of Tropical Medicine's Research Ethics Committee (Reference: 18-031).

A data sharing statement

For peer review only

For peer review only - http://bmjopen.bmj.com/site/about/guidelines.xhtml

The present study includes selected quotes to represent the content and themes across interviews. While requests for additional de-identified transcripts can be made to the authors, full interview transcripts cannot be made available as to do so would compromise anonymity and violate the terms of our ethical approval.

Separate documents from this main document

Figure 1: The IMPALA Organogram

Figure 2: The three-component framework for the cross disciplinary collaborative research process used in this study (adapted from Hall et al., 2012)

Reference

1. Koplan, J.P., et al., *Towards a common definition of global health.* The Lancet, 2009. **373**(9679): p. 1993-1995.
2. Stipelman, B.A., et al., *Mapping the Impact of Transdisciplinary Research: A Visual Comparison of Investigator-Initiated and Team-Based Tobacco Use Research Publications.* Journal of Translational Medicine & Epidemiology, 2014. **2**(2).
3. Ding, Y., J. Pulford, and I. Bates, *Practical actions for fostering cross-disciplinary global health research: lessons from a narrative literature review.* BMJ Global Health, 2020. **5**(4): p. e002293.
4. Zscheischler, J. and S. Rogga, *Transdisciplinarity in land use science – A review of concepts, empirical findings and current practices.* Futures, 2015. **65**: p. 28-44.
5. Michael O'Rourke, Stephen Crowley, and C. Gonnerman, *On the nature of cross-disciplinary integration: A philosophical framework.* Studies in History and Philosophy of Science Part C: Studies in History and Philosophy of Biological and Biomedical Sciences, 2016. **56**: p. 62 - 70.
6. Sonnenwald, D.H., *Scientific collaboration.* Annual review of information science and technology, 2007. **41**(1): p. 643-681.
7. Shiffman, J., *Four Challenges That Global Health Networks Face.* International journal of health policy and management, 2017. **6**(4): p. 183-189.
8. Al Khaldi, M., et al., *Challenges and prospects in the public health research system in the occupied Palestinian territory: a qualitative study.* The Lancet, 2018. **391**: p. S25.
9. Hall, K.L., et al., *A four-phase model of transdisciplinary team-based research: goals, team processes, and strategies.* 2012.
10. LSTM. *NIHR Global Health Research Unit on Lung Health and Tuberculosis in Africa at LSTM.* 2020 27/09/2021 [cited 2021 23/03/2020]; Available from: https://www.lstmed.ac.uk/impala.
11. Stokols, D., *Toward a science of transdisciplinary action research.* Am J Community Psychol, 2006. **38**(1-2): p. 63-77.

For peer review only - http://bmjopen.bmj.com/site/about/guidelines.xhtml

12. Verschuren, P., H. Doorewaard, and M. Mellion, *Designing a research project*. Vol. 2. 2010: Eleven International Publishing The Hague.
13. Ritchie, J., et al., *Designing and selecting samples*, in *Qualitative research practice: A guide for social science students and researchers*, J. Ritchie, et al., Editors. 2013, sage.
14. Fuqua, J., et al., *Transdisciplinary collaboration as a basis for enhancing the science and prevention of substance use and "abuse".* Substance use & misuse, 2004. **39**(10-12): p. 1457-1514.
15. Riessman, C.K., *Narrative methods for the human sciences*. 2008: Sage.
16. Davé, A., et al., *Case Study Review of Interdisciplinary Research in Higher Education Institutions in England*. 2016, Technopolis.
17. Kneipp, S.M., et al., *Nurse scientists overcoming challenges to lead transdisciplinary research teams.* Nurs Outlook, 2014. **62**(5): p. 352-61.
18. Mesmer-Magnus, J.R. and L.A. DeChurch, *Information sharing and team performance: A meta-analysis.* Journal of applied psychology, 2009. **94**(2): p. 535.
19. Stephenson, J., et al., *The Practice of Interdisciplinarity.* International Journal of Interdisciplinary Social Sciences, 2010. **5**(7).
20. Zscheischler, J., S. Rogga, and M. Busse, *The Adoption and Implementation of Transdisciplinary Research in the Field of Land-Use Science—A Comparative Case Study.* Sustainability, 2017. **9**(11): p. 1926.
21. Stokols, D., et al., *Evaluating transdisciplinary science.* Nicotine & Tobacco Research, 2003. **5**(Suppl_1): p. S21-S39.
22. Gavens, L., et al., *Interdisciplinary working in public health research: a proposed good practice checklist.* Journal of Public Health, 2017. **40**(1): p. 175-182.
23. Jeffrey, P., *Smoothing the Waters: Observations on the Process of Cross-Disciplinary Research Collaboration.* Social Studies of Science, 2003. **33**(4): p. 539-562.
24. Karp, G.G., et al., *Facilitating collaborative interdisciplinary research: exploring process and implications for leisure scholars.* Leisure/Loisir, 2016. **40**(2): p. 201-223.
25. Kirk-Lawlor, N. and S. Allred, *Group Development and Integration in a Cross-Disciplinary and Intercultural Research Team.* Environmental Management, 2017. **59**(4): p. 665-683.
26. Rolland, B., et al., *Operationalization, Implementation, and Evaluation of Collaboration Planning: A Pilot Interventional Study of Nascent Translational Teams.* Journal of Clinical and Translational Science, 2020: p. 1-20.
27. Spiller, K., et al., *Carnivalesque collaborations: reflections on 'doing' multi-disciplinary research.* Qualitative Research, 2015. **15**(5): p. 551-567.
28. Trussell, D.E., et al., *Negotiating the Complexities and Risks of Interdisciplinary Qualitative Research.* International Journal of Qualitative Methods, 2017. **16**(1): p. 1609406917711351.

For peer review only - http://bmjopen.bmj.com/site/about/guidelines.xhtml

For peer review only

For peer review only - http://bmjopen.bmj.com/site/about/guidelines.xhtml

Figure 1: The IMPALA Organogram

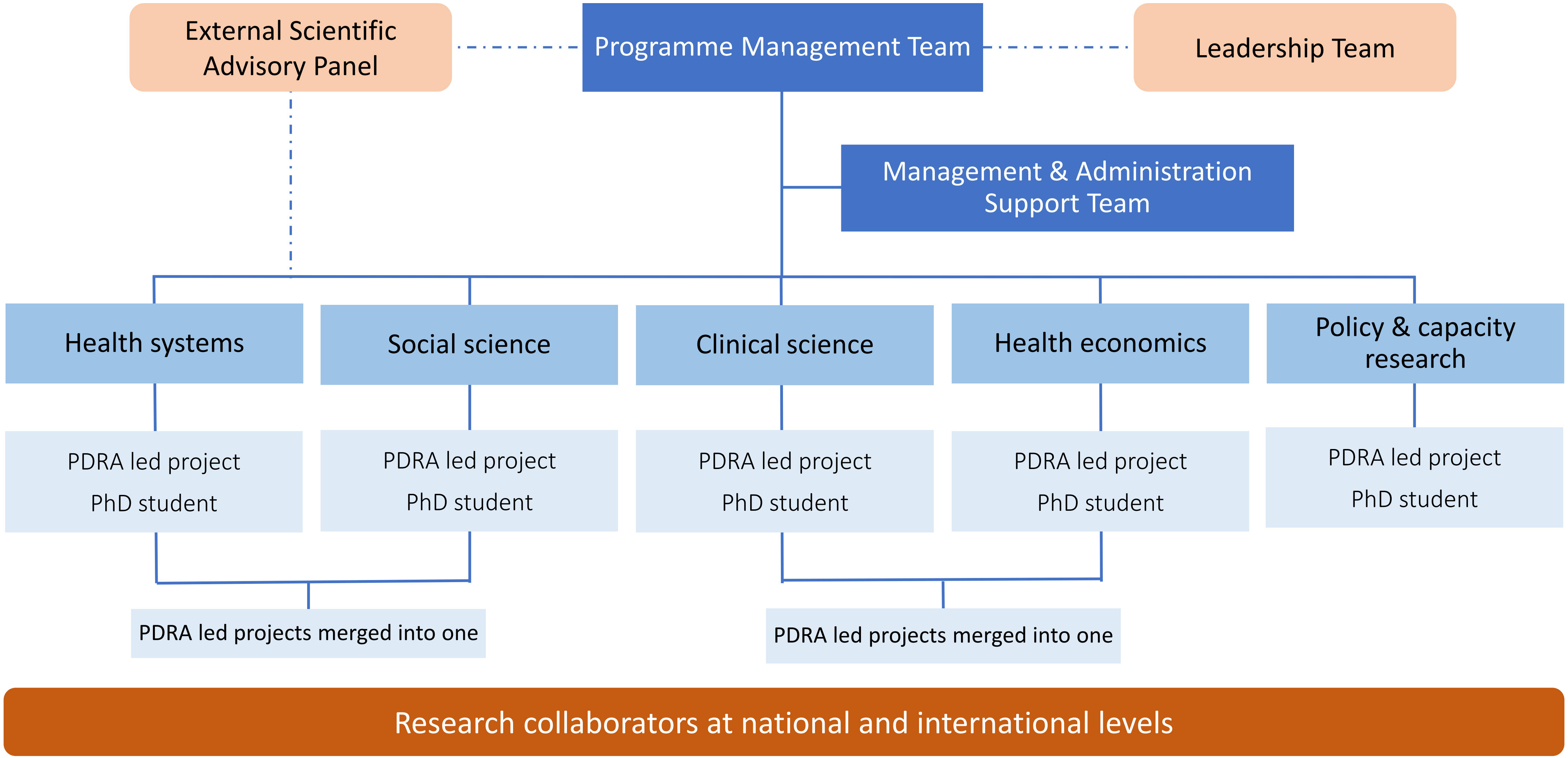

Figure 2: The three-component framework for the cross-disciplinary collaborative research process used in this study (adapted from Hall et al., 2012)

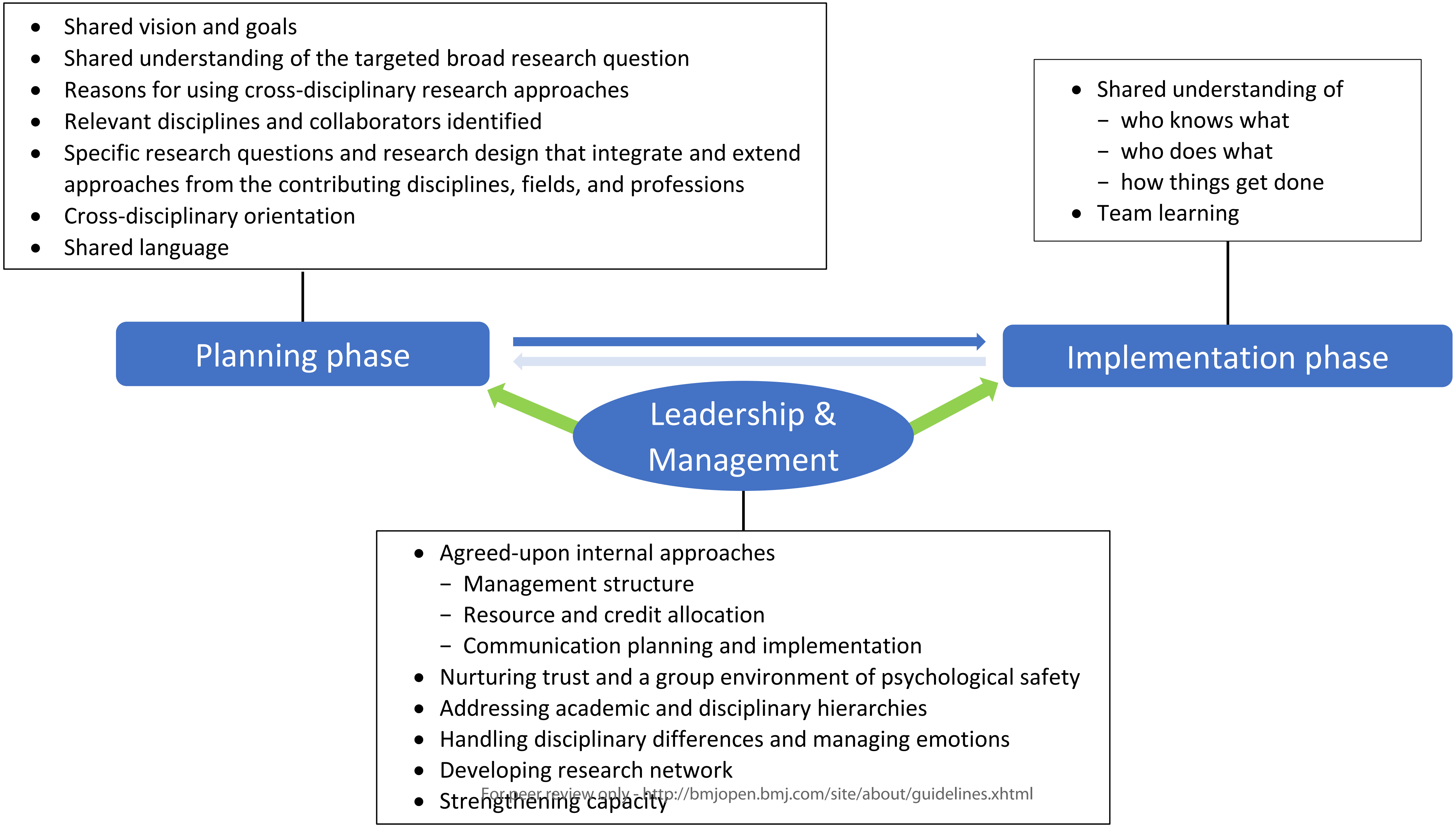

Version 7, 24 May 2018

IMPALA

Technical Proposal for

Multidisciplinary cross-cutting capacity development project (MUDI)

Capacity Research Unit, LSTM

Contents

Acronyms 3
1 Background 4
1.1 IMPALA 4
1.2 The two phase-1 applied research projects within IMPALA 5
1.3 Why a research on multiple disciplinary research? 6
1.4 Definitions 7
2 Aim of MUDI research project 7
3 Objectives for MUDI project 8
4 Key outputs 9
5 Activities 9
6 Integrated model of multidisciplinary implementation research process 13
7 Methodology 15
7.1 A scoping review 16
7.2 A baseline investigation 16
7.3 Tracking, understanding and learning from changes in multidisciplinary research 19
7.4 Synthesis of changes and learning in multi-disciplinary research in IMPALA. 19
7.4 Data management 20
7.5Data quality assurance 20
8 Ethical aspects 21
9 Beneficiaries of MUDI research findings 21
10 Dissemination plan 21
11 Timeline 22
12 References 22

Acronyms

COPD	Chronic Obstructive Pulmonary Disease
CRU	Capacity Research Unit
GCRF	Global Challenges Research Fund
LMICs	Low and Middle-Income Countries
LSTM	Liverpool School of Tropical Medicine
IMPALA	International Multiple Programme in Addressing Lung Health and TB in Africa
MUDI	Multiple Disciplinary Research Capacity Development Project
NIHR	National Institute for Health Research
SDGs	Sustainable Development Goals
TB	Tuberculosis

For peer review only - http://bmjopen.bmj.com/site/about/guidelines.xhtml

Section A – General details

Title: Multidisciplinary cross-cutting capacity development project (MUDI)
Discipline/s: Capacity Development
Lead investigator: Imelda Bates
Co-Investigators: Justin Pulford
Countries / study sites: Sudan, Tanzania, Uganda (for phase 1 non-embedded projects)

Section B – Project details

1 Background

1.1 IMPALA

The International Multidisciplinary Programme to Address Lung Health and TB in Africa "IMPALA" is a four-year collaborative programme funded by the National Institute of Health Research (NIHR) to establish an Africa-focused NIHR Global Health Research Unit for lung health and tuberculosis (TB) that will generate new scientific knowledge and implementable solutions for these high burden, under-funded and under-researched health problems. IMPALA comprises four phase-1 projects and five phase-2 projects, and they are: 1) two phase-1 (non-enbedded) applied research projects (Growing healthy lungs in Africa; and An integrated package of care for chronic lung disease: a community health systems analysis in Sudan and Tanzania); 2) two embedded, cross-cutting phase-1 projects (Multiple Disciplines: Research Capacity Development – this MUDI project, and Research Uptake for Policy); and 3) five phase-2 Ph.D students' applied research projects (TB prevalence and communities' coping strategies; Cost of illness and disease burden of chronic obstructive pulmonary disease (COPD) in LMICs; Improving referral uptake and tracking for lung diseases and TB in Kenya; Assessment of the impact of standardized ultrasound screening in TB diagnosis; and Operational modelling of the best pathway for chest radiography in the TB diagnostic pathway in Kenya). MUDI will take the two phase-1 applied research in IMPALA as case studies to generate robust evidence about what works for fostering multidisciplinary implementation research in what contexts, using Lung Health and TB as a pathfinder.

IMPALA is hosted by LSTM. IMPALA has an external scientific advisory board, a LSTM-based program leadership team, a LSTM-based project team including project leads for all the phase-1 and phase-2 project projects, an Africa-based team from 13 organizations in 11 countries in Africa, a cross-cutting training partnership for Ph.D. students with Lancaster University, and underpinning collaboration and partnership at international, African continent and individual African country level.

1.2 The two phase-1 applied research projects within IMPALA

One phase-1 applied research projects, growing healthy lungs in Africa, aims to answer a question which we quoted here from its concept note: “to what extent do socio-economic and clinical determinants, especially nutrition during pregnancy, associate with the societal burden of respiratory diseases as assessed by spirometry in children aged 6 to 10 and their families?”. It will be implemented in Uganda, and possibly also in the Gambia and South Africa. It has an Uganda country team, a LSTM project team, and other researchers based in Nigeria, the Gambia, South Africa, Ethiopia, and Tanzania.

The other, an integrated package of care for chronic lung disease: a community health systems analysis in Sudan and Tanzania, aims to generate evidence on the effectiveness and quality of an integrated approach to lung health in LMICs from health systems and community perspectives. It has a Sudan country team, a Tanzania country team, a LSTM project team, and other researchers based in Malawi.

These two projects will have scientific advises from the IMPALA external scientific advisory board, under the leadership of IMPALA leadership team, be jointly designed and implemented by researchers from country teams, LSTM and other above-mentioned countries in Africa. IMPALA’s underpinning collaborators and partners at international, African continent and individual African country level will join IMPALA and the two phase-1 applied research projects in translating research into impact. Diagram 1 summarizes the team structure of the two phase-1 applied research with IMPALA.

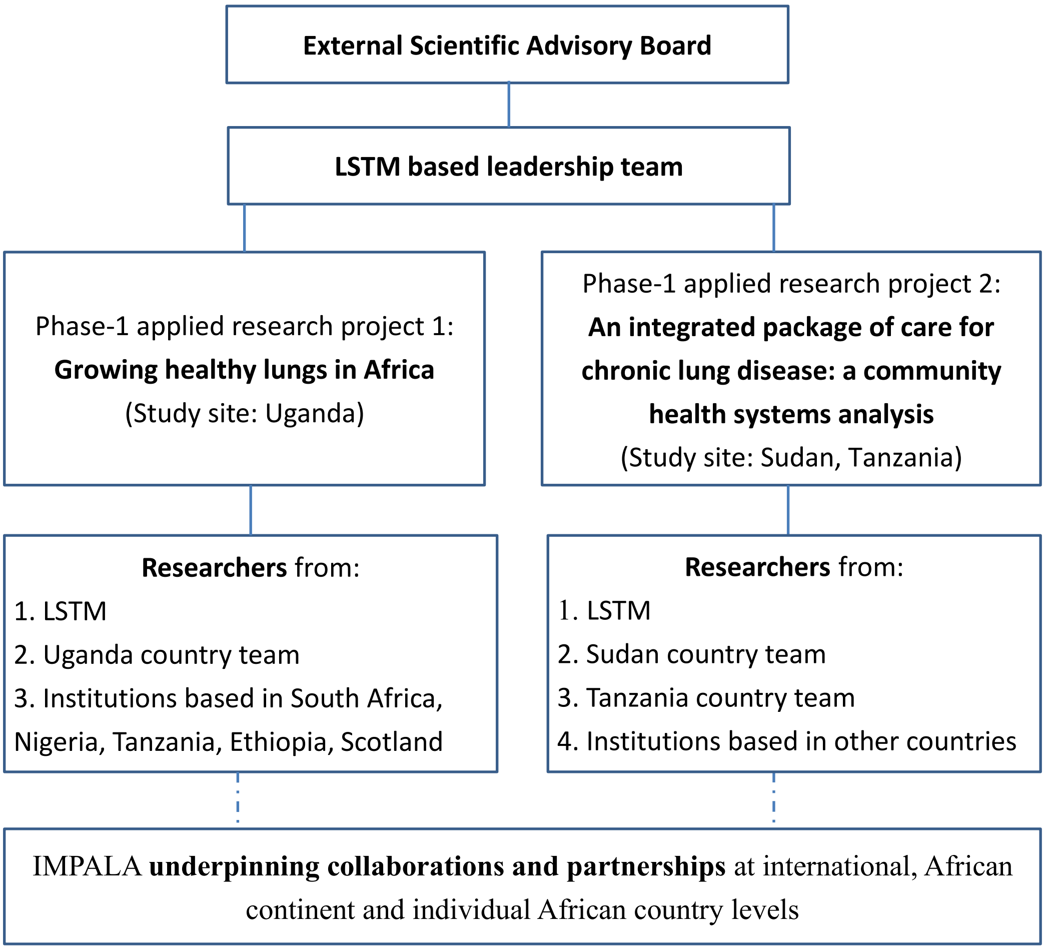

Diagram 1: The team structure of the two phase-1 applied research projects within IMPALA

1.3 Why a research on multidisciplinary research?

Research that involves multiple disciplines is inclusive of multi-, inter-and trans-disciplinary research [1]. This kind of research is essential for achieving the deep interconnected and cross-cutting Sustainable Development Goals (SDGs) [2], and much literature on multi-, inter-, and trans-disciplinary research argues that it has a key role to play in addressing the grand challenges society faces [3,4,5,6,7]. Also, discoveries are said to be more likely on the boundaries between fields where insights can reorient or increase knowledge [8,9]. Many international development funding Calls therefore demand this type of research [8,10,11,12,13], and interdisciplinarity was the focus of a 2015–16 report from the Global Research Council [3]. Not surprisingly, interdisciplinary research has been on the rise since middle 1980s especially in 21st century [14]. There are some informatic books on interdisciplinary research processes and theory with a focus on individual and institutional level interdisciplinary research [9, 15, 16]. However, there is very little robust evidence to guide the design of multi-, inter-, and trans-disciplinary projects. The establishment of fair and effective assessments of interdisciplinary research proposals is a challenge for many of the

For peer review only - http://bmjopen.bmj.com/site/about/guidelines.xhtml

funding agencies [3]. In addition, quality metrics for assessing the effectiveness of research with multiple disciplines are scarce. Without quality metrics it is hard to assess the assumed role of multi-, inter-, and trans-disciplinary research in generating new knowledge and addressing grand challenges society faces.

Both the IMPALA phase-1 projects incorporate multiple disciplines. Using rigorous research methods, MUDI project will use the two projects as case studies to prospectively explore and compare the effectiveness of the different approaches that the two projects use to generate outputs through involving multiple disciplines. An important part of the MUDI project, which is represented within the objectives, is real-time sharing of emerging lessons across IMPALA so that improvements to the programme can be made in real time thus enhancing the effectiveness and value for money of the programme.

1.4 Definitions

There are many articles on multi-, inter- and trans-disciplinary research, and it seems there is still no consensus on how to define these types of research. Multi-, inter- and trans-disciplinary research refer to the involvement of multiple disciplines to varying degrees on the same continuum of integration of disciplinary perspectives [1,17-20] which are disciplines' unique views of reality in a general sense [8]. There is not yet a general term in the literature that is inclusive of multi-, inter-and trans-disciplinary research, although one article proposed "multiple disciplinary research" [1]. The involvement of multiple disciplines in the IMPALA two phase-1 applied research is evolving on the continuum of integration of disciplinary perspectives and unspecified. Therefore, we proposed a definition for multidisciplinary research as used in this study.

Multidisciplinary research is research that uses knowledge, study design and methodology from multiple disciplines.

Research capacity strengthening

In the context of this MUDI project, our definition of research capacity strengthening is "the process by which individuals, organizations, and societies develop abilities (individually and collectively) to perform functions effectively, efficiently and in a sustainable manner to define problems, set objectives and priorities, build sustainable institutions and bring solutions to key national problems" [21].

2 Aim of MUDI research project

To generate robust evidence about what works for fostering multidisciplinary implementation research, and in what contexts, using Lung Health and Tuberculosis (TB) as a pathfinder. Also, to develop evidence-based and transferable recommendations for actions (and possibly a benchmark) that can catalyze effective and sustainable multidisciplinary implementation research in low and middle-income

For peer review only - http://bmjopen.bmj.com/site/about/guidelines.xhtml

countries (LMICs).

The two IMPALA phase-1 projects involve multiple disciplines and MUDI will use these two projects as case studies, to investigate promotors and inhibitors of multidisciplinary implementation research that occur at three levels – individual, IMPALA programme (i.e. two phase-1 projects), and beyond the IMPALA programme by comparing with lessons from other projects and published studies. Figure 1 presents a visualized approach to strengthening capacity for (MUDI) research [22].

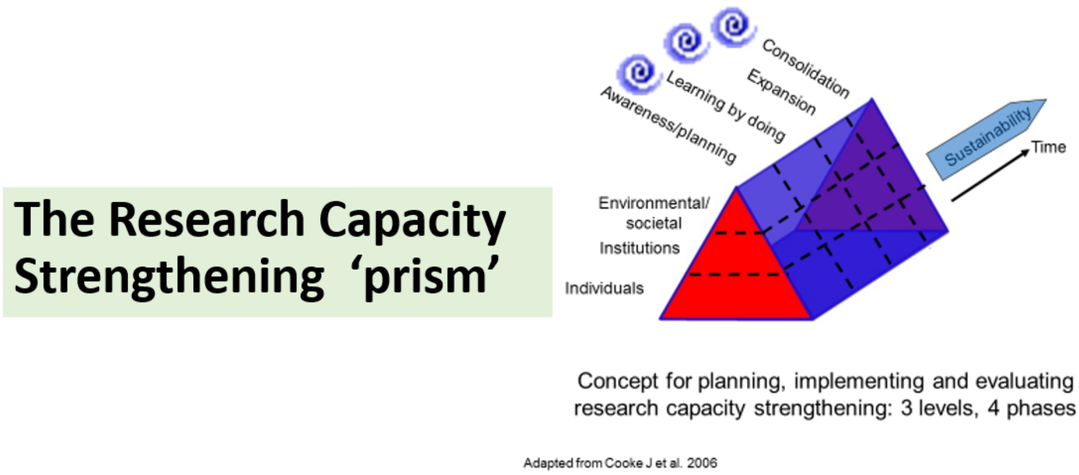

Figure 1: Visualizing the approach to strengthening capacity for (MUDI) research

3 Objectives for MUDI project

1) Map researchers' expertise and competencies in multidisciplinary research, and collaborations among multiple disciplines within IMPALA.
2) Identify enablers and barriers to performing multidisciplinary implementation research within the context of PDRA led projects.
3) Identify commonalities and differences in successful and less successful approaches for multidisciplinary implementation research among the projects, and the limits of transferability of these approaches across the projects.
4) Develop evidence-based and transferable recommendations for actions (and possibly a benchmark) that can catalyze effective and sustainable multidisciplinary implementation research in LMICs.
5) Share the recommendations within and beyond IMPALA and strengthen IMPALA by sharing relevant learning and implementable actions from related research (e.g. on research uptake, consortia management)

4 Key outputs

1) A scoping review of multidisciplinary research enablers and barriers, processes and theory.
2) A technical research proposal with evidence informed tools for data collection.
3) Baseline report on mapping out expertise and collaboration among multiple disciplines across IMPALA, first steps of the program and the two projects, enablers and barriers at three levels (individual, within IMPALA and beyond the program)
4) Annual learning reports
5) Final report (and peer-reviewed publication) to include a summary of comparative, prospective data collection on multidisciplinary research enablers and barriers in the two projects, and evidence-based recommendations for actions and metrics for effective and sustainable multidisciplinary research LMICs

5 Activities

1) From December 2017 to May 2018: conduct a scoping review of the literature to identify lessons and examples of good practice in multidisciplinary research.
2) From February to May 2018: using the scoping review and consultations within and beyond IMPALA to develop evidence-informed tools for collecting baseline and ongoing data on multidisciplinary research enablers and barriers at three levels outlined above. Each of the two projects will be treated as an individual case study for generating baseline and prospective, ongoing data. The tools for collecting data include surveys, interviews and focus group discussions. Submit protocol with ethics forms to LSTM for ethical approval.
3) From June to August 2018: Map out multidisciplinary research occurring in the two projects (high level data). Select specific areas for in-depth exploration (in-depth data) across the projects to maximize diversity. Collect and analyze data at baseline and prospectively during programme life-time.
4) From January 2019-March 2021: Share findings and lessons as they emerge in real-time to improve the effectiveness of multidisciplinary research across the Phase 1, Phase 2 and future projects. Sharing mechanisms will include reports of baseline data by month 18 and final reports in the last 6 months of the project.
5) Around March 2021: The use of a common framework for data capture means that we can collate all findings across the two projects to generate transferable, evidence-based recommendations for actions and metrics to catalyze and measure effective and sustainable multidisciplinary research in LMICs (by programme end). This may form the basis of an initial benchmark against which future multidisciplinary research efforts can be measured.

We use table 1 to summarize project activities, expected outputs, why, when and how to carry out these activities. Section 7 Methodology part will give more details on how to conduct these activities. As it is an iterative approach to explore the stated objectives, relevant data will be collected at the beginning, mid-and also end-of-project and lessons will be fed back into to project as they emerge to guide improvements in real time.

For peer review only - http://bmjopen.bmj.com/site/about/guidelines.xhtml

Table 1: MUDI project's main activities, expected outputs, why, when and how to conduct these activities

	Main activities	Outputs	What objectives to serve	When	How	Tools and materials
1)	Conduct **a scoping review** of the literature to identify lessons and examples of good practice in multidisciplinary research	A scoping review	Objectives 2-5: Barriers, facilitators, commonalities, differences, recommendations	Dec.2017 - May2018	Follow a scoping review methodology	A computer/laptop, Database, books,
2)	Develop **a technical proposal** for MUDI including evidence-informed **tools for collecting** baseline, ongoing data and also final evaluation **data**	A technical proposal with tools for baseline data collection	All objectives	Feb.-May. 2018	Informed by the scoping review with consultations and team discussions	Located literature and a computer/laptop
3)	Apply for **ethics** approval at LSTM	Ethics approval from LSTM	All objectives	Mar.-Apr. 2018	Follow LSTM REC requirements	The technical proposal and tools and other ethics application materials
4)	**Beginning-of-project** data collection, analyzing data, and writing a report	A beginning-of-project report	Objective 1: Mapping expertise and collaboration; Objective 2: enablers and barriers.	Jun.-Aug. 2018	1) Survey<br>2) semi-structured interview	A questionnaire and three topic guides (for IMPALA Directors, two projects' researchers, and external scientific advisory board members)
5)	Select specific areas for in-depth exploration across the projects to maximize diversity.	Selected areas for in-depth exploration	Objectives 2-5	June 2018	to be determined (TBD) depending on the areas identified from the beginning-of-project study	TBD (but likely to predominantly interviews)

6)	Prospective and ongoing data collection, analyzing and writing annual learning reports	Annual learning reports (e.g. to understand and solve barriers to implementing multidisciplinary interactions) to improve ongoing programme activities	Objectives 2-5	Jun.-July 2019, Apr.-May 2020, also every three months to collect monitoring data	1) Survey<br>2) semi-structured interviews<br>3) focus group discussions	1) A monitoring and learning form for IMPALA program management team<br>2) a monitoring and learning form for interaction with non-academic stakeholders and implementation barriers<br>3) Three topic guides as listed above
7)	Share findings and lessons within IMPALA as they emerge in real-time	Recommendations for the implementation of IMPALA	Objective 4 & 5	Jan. 2019-Oct. 2020	Through internal meetings and annual learning reports	Preliminary findings from the prospective and ongoing data
8)	End-of-project investigation (data collection, analyzing data, and writing a report)	An end-of-project report and possibly a benchmark to measure multidisciplinary research efforts	Objectives 2-5	Nov.2020-Mar.2021	1) Survey<br>2) semi-structured interviews<br>3) focus group discussions	A survey and three topic guides as listed above

6 Integrated model of multidisciplinary implementation research process

We will use an integrated model of multidisciplinary implementation research process to guide us in exploring how to foster multidisciplinary implementation research. The model adopts the most simplified form of interdisciplinary research process [9], which is from problem to insights of different disciplines, to integration of insights, and to understanding. We extend the "understanding" to "influencing practice and policy", as an assumed consensus in literature is that multi-, inter-and trans-disciplinary research cannot only generate new knowledge but also solve complex problems. We add influential factors to the model which include societal, institutional-/project- factors and researchers informed by our scoping review which found barriers and facilitators of multidisciplinary research could in general be divided into the three categories. More information is provided in Box 1. In addition, to influence practice and policy, multidisciplinary research is expected to interact with non-academic stakeholders and address implementation barriers if there is any. Therefore, we take principles of implementation research into account in the model with emphasis on the involvement of non-academic stakeholders and on addressing implementation barriers informed by literature [23]. Figure 3 demonstrates the integrated process.

Box 1: Influential factors informed by our scoping review and guided by the research capacity strengthening "prism"

1) At individual level
e.g.: education, research and other work background, competencies related to multiple disciplinary research, opinions for such collaboration, expectations, etc.;

2) At institutional/project level
e.g.: leadership, why work together, expectations, how to work together, whether promote "T-shaped" researchers* and nurture individuals' skills in constructive dialogue, so far what have been planned or done, multiple-discipline collaborations, how are the first steps, good practice examples and challenges, what's next; and

3) At environmental/societal level
e.g.: context information, including countries, cultures, disease burdens, collaboration history among individuals and among organizations, links with other organizations/persons which may facilitate the pathways to impact, performance evaluation systems for individuals, institutions and projects, funders and publishers etc.

*"T-shaped" researchers are interpreted as "those capable of breadth and depth (T-shaped) and able to engage in constructive dialogue and co-creation" [24], and we present "the journey to T" in Figure 2[24].

For peer review only - http://bmjopen.bmj.com/site/about/guidelines.xhtml

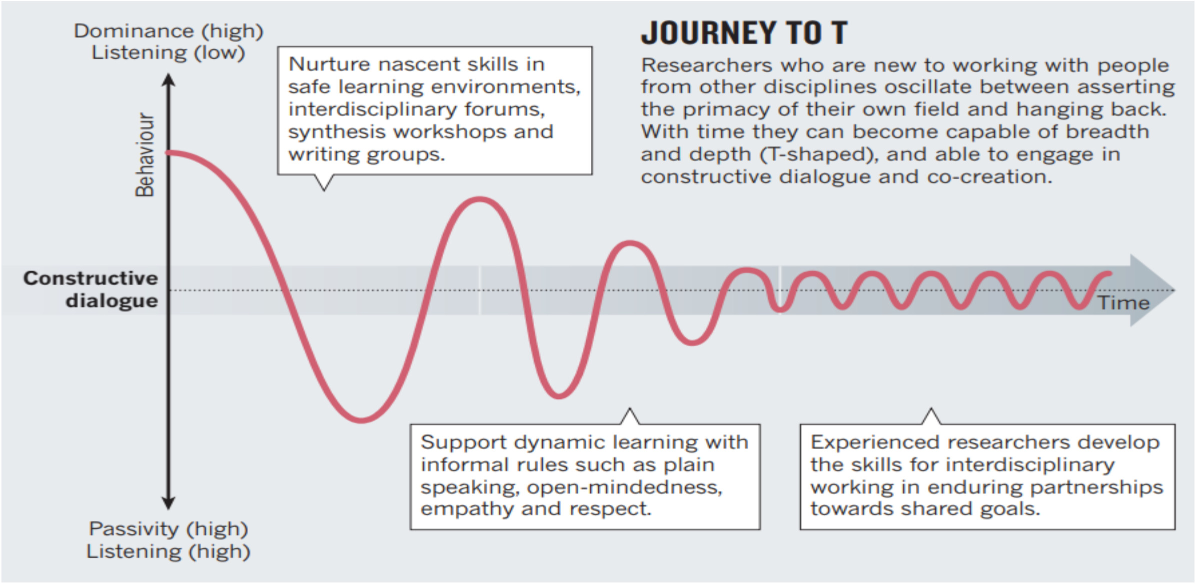

Figure 2: Researchers' journey to T (from Brown R.R. et al., 2015)

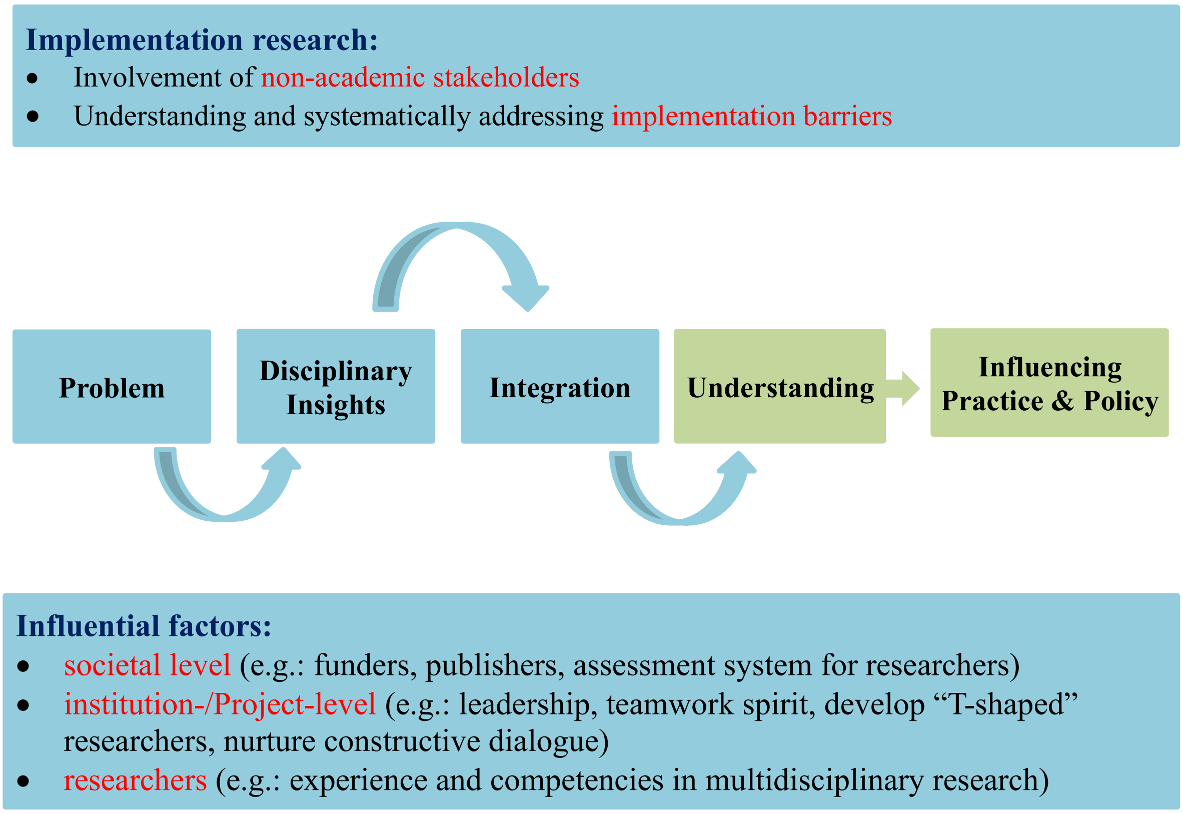

Figure 3: Integrated model of multidisciplinary implementation research process

To elaborate details on the process, we develop an integrated model of the multidisciplinary implementation research process with 15 steps as shown in Table 2. These steps are based on the model of the integrated interdisciplinary research process [8]. We added Section C "Communicating the understanding to influence practice and policy" based on a training workshop on policy uptake provided by the IMPALA project in January 2018.

Table 2: An Integrated Model of the Multiple Disciplinary Implementation Research Process

A. Drawing on disciplinary insights
1) Define the problem or state the research question<br>2) Justify using a multidisciplinary implementation approach<br>3) Identify relevant disciplines<br>4) Conduct the literature search<br>5) Develop adequacy in each relevant discipline<br>6) Analyze the problem and evaluate each insight or theory
B. Integrating disciplinary insights
7) Identity conflicts between insights and their sources<br>8) Create common ground between insights<br>9) Construct a more comprehensive understanding<br>10) Reflect on and test the understanding
C. Communicating the understanding to influence practice and policy
11) Planning for evidence uptake<br>12) Policy analysis including stakeholder analysis<br>13) Developing engagement strategies<br>14) Packaging evidence<br>15) Monitoring and evaluation

7 Methodology

The methods for this study comprise:

- a scoping review, to inform the study concept, design and data collection tools
- baseline mapping of multidisciplinary expertise and interactions across IMPALA
- tracking and understanding changes in multidisciplinary research collaborations across IMPALA compared to baseline (using a monitoring and learning form which will be developed to track the findings from the baseline survey and interviews)
- synthesis of lessons learnt and implications beyond IMPALA

In a nutshell for data collection, we will do two rounds of online survey and four rounds of interviews/focus group discussions.

- For each round of online survey, we will have around 50 respondents according to our sampling framework, the IMPALA team directory.
- For each round of interviews, we will aim to do enough interviews to reach saturation of the data.
    - For beginning-of-project data generation, we plan to interview 26 people selected to maximize diversity in disciplinary expertise, gender and geographical location.
    - These individuals will be interviewed again for ongoing data collection and we will conduct a focus group discussion with the four PDRAs in IMPALA, in both 2019 and 2020;
    - We also plan to do another round of interviews at the end of PDRA led projects, also for 26 people.
    - Depending on the results of the survey and initial interviews, we may select additional participants from the IMPALA directory to explore specific topics through interviews.

For peer review only - http://bmjopen.bmj.com/site/about/guidelines.xhtml

7.1 A scoping review

Aims to:

1) provide a descriptive synthesis of barriers and enablers to multidisciplinary research;
2) inform the development of an integrated model of multidisciplinary research process as an analytical framework for the project; and
3) guide development of data collection tools and identify indicators that may be used to assess project effectiveness.

We will adopt the stages 1–6 of the advanced 'scoping' methodology [25] based on the original framework [26].

7.2 A beginning-of-project investigation

Aims to:

1) map researchers' experience and competencies in conducting multidisciplinary research; (objective 1)
2) map multidisciplinary research collaborations within PDRA led projects to select specific areas for in-depth exploration to maximize diversity; (objective 1)
3) collect environmental/ societal context information for IMPALA and PDRA led projects; (objective 3)
4) explore expectations of IMPALA leaders and researchers of the two phase-1 applied research for, respectively, IMPALA, PDRA led projects, and themselves in participating in IMPALA; (objective 3)
5) learn how IMPALA leaders and researchers of the two phase-1 applied research identified research questions, what disciplines have been involved; what leaders have done and planned to do to facilitate multidisciplinary research; what perspectives from other disciplines and non-academic stakeholders have researchers identified for PDRA led projects and how do they identify the perspectives; what are the barriers and facilitators for multidisciplinary research so far during the startup phase. (objectives 2-5)

Data collection methods:

1) an online survey
2) semi-structured interviews

The online survey

The survey will take place mainly in May and June 2018 with more details provided in Table 3.

Table 3: details on the online survey

	**Items**	**Details**
1)	Respondents	a) Around 50, include all these listed in IMPALA Team Directory (excluding administration and finance staff members).
2)	Content	a) The respondent's basic information, i.e.: affiliation, job title, age, gender, education background;<br>b) Role in IMPALA, including title and general responsibility<br>c) Experience and confidence in conducting multidisciplinary research based on recommended competencies [27] and we present them in Table 4.

3)	Mode of administration of the questionnaire	a) Bristol Online Survey (BOS), computer assisted self-administration.
4)	Privacy	a) Not anonymous but personal information will not be shared out of CRU.<br>b) Reason: the questionnaire asks respondents for their names and organizations to allow us to locate candidates for semi-structured interviews for prospective ongoing data collection.
5)	Timeline	a) Feb.-Mar.2018, questionnaire development informed by the scoping review.<br>b) 15-16 Mar.2018, pilot with researchers at CRU who are not involved in the design of the questionnaire.<br>c) 19 Mar. 2018, revision & finalization<br>d) 19 April 2018, the first day of IMPALA annual meeting.<br>e) 19 April 2018, introduce the questionnaire survey to all attendees at the annual meeting in person by an oral presentation and answer questions if any.<br>f) End of May or early June, send out an email to all respondents to introduce the questionnaire survey and the link to the online questionnaire.<br>g) June, main survey period<br>h) 30 June 2018, end of survey.
6)	Approaches to improve responsiveness and data quality	a) Delivering the survey during the annual meeting period when all respondents are supposed to attend and internet access is guaranteed.<br>b) Introduce the questionnaire survey through an email and also by an oral presentation to all attendees.<br>c) Reminder sent to those who did not complete or did not attend the meeting, 1 week after the invitation email. A total of 2 invitations will be sent to all eligible participants, at least 7 days apart. Eligible participants will have a minimum of 4 weeks in which to complete the survey.

Table 4: Competencies in multidisciplinary Research (adapted from Gebbie KM et.al., 2007)

The scholar who has completed doctoral work with an emphasis on multidisciplinary research is able to:
**Conduct research**<br>• Use theories and methods of multiple disciplines in developing integrated theoretical and research frameworks by yourself;<br>• Investigate research questions using theories and methods of more than one discipline by yourself;
**Communicate**<br>• Advocate multidisciplinary research in developing initiatives within your discipline (s);<br>• Proactively seek an exchange of perspectives with those from other disciplines;<br>• Respectfully disagree perspectives of other disciplines;<br>• Read academic articles outside your discipline;<br>• Communicate at least on monthly basis with scholars from a discipline other than your own;<br>• Share research from your discipline in language understandable by those outside your discipline;<br>• Modify your own perspectives on research questions or methods as a result of interactions with colleagues from fields other than your own;<br>• Disseminate multidisciplinary research results within your discipline;<br>• Disseminate multidisciplinary research results outside your discipline;

For peer review only - http://bmjopen.bmj.com/site/about/guidelines.xhtml

Interact with others
• Engage colleagues from other disciplines to gain their perspectives on research problems;
• Interact in training exercises with scholars from other disciplines;
• Attend scholarly presentations by members of other disciplines;
• Collaborate respectfully and equitably with scholars from other disciplines to develop integrated research methods to achieve research aims;
• Draft funding proposals for multidisciplinary research projects /programs in partnership with scholars from other disciplines.

Semi-structured Interviews

1) We will start to do semi-structured interviews after we received ethics approval from LSTM, aiming to finish the interviews remotely by 31 August 2018; we will mainly use Skype calls to interview non-LSTM colleagues. LSTM colleagues will have face-to-face interviews after each annual meeting.
2) We developed our four topic guides mainly following the Integrated Model of the Multidisciplinary Implementation Research Process and attached it as Attachment 2.
3) Purposive sampling. We will conduct interviews with the IMPALA External Scientific Advisory Panel members, IMPALA program leaders (directors), and researchers who participate in the two IMPALA phase-1 applied research projects including these from LSTM, country teams, and these based in countries in Africa but not the intervention countries. To maximize variation, we will consider seniority, gender, geography and discipline in locating interviewees. The sample of the interviews is presented in Table 5.
4) The interviews will be audio-recorded with permission to check all points are correctly captured.

Table 5: Sampling strategy and subjects for the semi-structured interviews

**Semi-structured interviews**		
**Group**	**Role**	**Number**
External Scientific Advisory Panel	Independ chair;<br>One senior applied health research academics for each one of the 5 disciplines;<br>A non-voting observer	2
Directorate	Director	3
	Co-Deputy Director	
	Co-Deputy Director	
Researchers	4 Discipline leads and 4 PDRA from LSTM, 2 other core members	9 (one is a Co-Deputy Director)
	Sudan country team (1lead)<br>Tanzania country team (3 researchers from 3 organizations)<br>Uganda country team (1 core members)	5
	Researchers from institutions in South Africa, Nigeria, Ghana, Malawi, Scotland, Kenya, Cameroon or Ethiopia	7
**Total**		26

For peer review only - http://bmjopen.bmj.com/site/about/guidelines.xhtml

7.3 Tracking, understanding and learning from changes in multidisciplinary research

Aims to:

1) map changes in, and development of, multidisciplinary research collaborations and select specific areas for in-depth exploration based on the progress of the two projects and the results of the baseline investigation; (objective 1)
   For example:
   a) within a discipline among researchers with varied experience and competencies in multidisciplinary research;
   b) within a multidisciplinary research project among multiple disciplines in terms of integrating perspectives from each discipline (be it in terms of concepts, theories and methods); and
   c) between program level leadership and project level technical research group especially in developing techniques in multidisciplinary research.
2) collect prospective, ongoing data for in-depth exploration on multiple-discipline research across the projects, keep analyzing barriers and facilitators, and provide feedbacks to IMPALA leadership team and the project teams for learning purpose; (objectives 2 and 5)

Tools:

1) a monitoring and learning form for IMPALA (Annex 3)
   a) It includes program level activities in nurturing constructive dialogue and developing T-shaped researchers;
   b) On quarterly basis and will be filled by the program manager.
2) a monitoring and learning form for PDRA led projects (Annex 4)
   a) It covers activities in involving non-academic stakeholders, implementation barriers and activities in solving the barriers;
   b) On quarterly basis and will be filled by PDRAs from the four disciplines.
3) semi-structured interviews;
   a) We will use similar sampling strategies as in the baseline investigation;
   b) The topic guides will continually follow the integrated model of the multiple-discipline implementation research process similar to these in the baseline investigation and may be revised after the baseline.
   c) Once per year and twice in total and will mainly take place around IMPALA's annual meetings;
   d) We will carry out interviews with non-LSTM people mainly during annual meeting and with LSTM colleagues mainly in Liverpool in person.

7.4 Synthesis of changes and learning in multi-disciplinary research in IMPALA

Aims to:

1) measure researchers' changes in self-reported multidisciplinary competencies; (objectives 3-5)
2) learn what techniques to promote and implement multidisciplinary research have developed at the program, project and individual levels at the end of the program compared to baseline; (objectives 3-5)
3) synthesize the data captured during the baseline investigation, the ongoing data

collected during the implementation period, and additional data through a final evaluation to generate transferable, evidence-based recommendations for actions to faster research involving multiple disciplines; (objectives 3-5)

4) synthesize the data to develop metrics to catalyze and measure effective and sustainable multidisciplinary research in LMICs, and form the basis of an initial benchmark against which future multidisciplinary research efforts can be measured. (objectives 3-5)

Tools:

1) an online survey
    a) the same sample and survey as in the baseline;
    b) similar approach in delivering the survey (Bristol Online Survey, self-administrated with an email introduction to all respondents. A total of 3 invitations will be sent to all eligible participants, at least 7 days apart. Eligible participants will have a minimum of 4 weeks in which to complete the survey.
2) semi-structured interviews
    a) will be similar to former interviews, purposive sampling, covering leaders, researchers, and external program advisory panel members, with topic guides followed the integrated model;
    b) as the data are collected and analyzed, when an interpretative framework has been constructed, the sampling strategy will change from largely purposive to largely theoretical to build on the developing theory as suggested in literature [28].

7.4 Data management

Data Entry

Quantitative data entry and qualitative data notes will be completed by staff members at the CRU, LSTM. The data will be saved on a laptop with password protection and with a back-up on CRU's shared drive with password protection.

Data Analysis

The Survey data will be entered into Excel, summarised and presented graphically, and where appropriate, semi-quantitatively (e.g. % agreement with statements). Qualitative analysis software such as NVivo, or manual analysis, will be used to code qualitative data from the interviews. Content analysis will be mainly used to analyze the qualitative data. We will analyse the qualitative data using the framework approach, which is essentially thematic analysis approach applicable to applied research. We will work through the fifteen steps of the integrated model of multidisciplinary research process, our analytical framework that is stated in this proposal in section 6 and displayed in Table 2, with the PDRA taking the lead on managing and coding data, developing the initial thematic framework, and on discussing emerging themes with other members of the CRU research team.

We will describe and compare the online survey results between the baseline and the end of programme to see whether there are any change in competencies at individual level in multidisciplinary research. We will triangulate data on education background and competencies in multidisciplinary research between qualitative and quantitative data.

7.5Data quality assurance

1) All data will be collected with tools informed by the scoping review;
2) A well trained experienced interviewer will conduct the interviews;

3) Some questions will be asked of more than one individuals to triangulate;
4) Interviews will be recorded and checked against notes/transcripts;
5) We will come back to related individuals and organizations to clear queries raised from individual questionnaire survey and ongoing data collection, and will also check for completeness and consistency after each interview;
6) Data will be double coded after removing individual identification information;
7) Data interpretation will be done by discussions among researchers.

8 Ethical aspects

The ethical integrity in this study is a high priority. We take every measure to protect the rights of the human subjects who participate in this research and adhere to the ethical principles of respect, beneficence, and justice as defined by the Research on Human Subject.
Researchers from CRU LSTM will apply for ethics approval from LSTM Research Ethics Committee. We will make clear to all participants of all ages that they are under no obligation to participate in the study. All participants will be assured that there will be no negative consequences if they choose not to participate. The researchers shall obtain informed consent from all participants. Semi-structured interviews will be conducted in a private setting to dissuade others from hearing the conversations. The researchers will assure the participants anonymity, confidentiality of information they give and will protect the data and its use for the purpose of this study only.

9 Beneficiaries of MUDI research findings

In addition to partners directly involved in IMPALA, external beneficiaries of our MUDI research include research and development funders (e.g. National Institute for Health Research, Wellcome Trust, Department for International Development, Royal Society, Global Challenges Research Fund), the action- and policy-oriented research community, and agencies with strategy, planning and think tank functions (e.g. Alliance for Accelerating Excellence in Science in Africa, African Academy of Sciences, World Health Organization, etc.).

10 Dissemination plan

The study findings will be disseminated in various methods and forums, that includes
1) Submit the final report through IMPALA to the NIHR.
2) Share the report within IMPALA
3) Brief note/brochures with points for wider public and client use
4) International Conferences.
5) Publication in scientific paper
6) Project annual meetings
7) Other means, such as in-country meetings organized by governmental and non-governmental as well as international organization, that may be attended by representatives from in-country academics and relevant stakeholder organizations and institutions.

For peer review only - http://bmjopen.bmj.com/site/about/guidelines.xhtml

11 Timeline

This project will be implemented July 2017 to March 2021 and Table 4 presents its timeline.

Table 4: Timeline of the MUDI project

	Jul.-Nov. 2017	Dec.2017-May 2018	June-Nov.2018	Dec.2018-May 2019	June-Nov.2019	Dec.2019-May 2020	June-Oct.2020	Nov.2020-Mar.2021
Recruit/start up	x							
Scoping review + consultation		x						
Ethics, tools, consult, finalise		x						
Case studies x2 beginning, ongoing and end-or-project data collection		x	x	x	x	x	x	x
Beginning-of-project data analysis + report			x					
Dissemination and share emerging learning			x	x	x	x	x	x
Compare and contrast case studies; extract lessons and recommendations (develop benchmark)					x	x	x	x
Consult, develop and agree good practice recommendations (and benchmark?) for multidisciplinary research in LMICs							x	x
Publication (peer reviewed)		x	x		x			x

12 References

1) United Nations. Transforming our world. The 2030 Agenda for Sustainable Development. A/RES/70/1. Available from: https://sustainabledevelopment.un.org/content/documents/21252030%20Agenda%20for%20Sustainable%20Development%20web.pdf [accessed 15 February 2018]
2) DJS Research. (2016). Interdisciplinarity, Survey Report for the Global Research Council 2016 Annual Meeting
3) Ledford H. (2015) How to solve the world's biggest problems. *Nature* 525,308–311 doi:10.1038/525308a
4) Nature. (2015) Why interdisciplinary research matters. *Nature* 525, 305 doi:10.1038/525305a.
5) Hallegatte. S & Mach. K (2016) Make climate-change assessments more relevant. *Nature* 534, 613–615 doi:10.1038/534613a
6) Marc Fleurbaey, M., Bouin, O., Djelic M-L., Kanbur R., Laborde C, Nowotny H, Reis, E., Weber,

E., Wieviorka, M. & Zhang, X. (2016) Policy: Social-progress panel seeks public comment. *Nature* 534, 616–617 doi:10.1038/534616a

7) Rylance, R. (2015) Grant giving: Global funders to focus on interdisciplinarity. *Nature*525,313–315 doi:10.1038/525313a
8) Repko A.F., Szostak R. (2017) *Interdisciplinary Research, process and theory*. Third Edition. SAE Publications.Inc. Los Angeles
9) Arts & Humanities Research Council. Available from: http://www.ahrc.ac.uk/funding/research/ [accessed 15 February 2018]
10) Economic and Social Research Council. Strategic Plan 2015. Available from: http://www.esrc.ac.uk/files/news-events-and-publications/publications/corporate-publications/strategic-plan/esrc-strategic-plan-2015/ [accessed 15 February 2018]
11) National Institute for Health Research. Funding Programmes. Available from: https://www.nihr.ac.uk/funding-and-support/funding-for-research-studies/funding-programmes/ [accessed 15 February 2018]
12) Global Challenges Research Fund, Research Councils UK. Available from: http://www.rcuk.ac.uk/funding/gcrf/ [accessed 15 February 2018]
13) Noorden R.V. (2015) Interdisciplinary research by the numbers. *Nature* 525, 306–307 doi:10.1038/525306a
14) Frickel, S., Albert, M., Prainsack, B. & Nowotny, H. (2016) *Investigating Interdisciplinary Collaboration: Theory and Practice across Disciplines* Rutgers University Press. New Brunswick, New Jersey and London.
15) The National Academies of Sciences Engineering Medicine. (2005) *Facilitating Interdisciplinary Research* The National Academies Press. Washington, D.C.
16) OECD (1998) Interdisciplinarity in Science and Technology, page 4.
17) Bruce, A., Lyall, C., Tait, J. & Williams, R. (2004) Interdisciplinary integration in Europe: the case of the Fifth Framework programme. *Futures* 36, 457–470.
18) Aboelela, S.W., Larson, E., Bakken, S., Carrasquillo, O., Formicola, A., Glied, S.A., Haas, J. & Gebbie, K.M. (2007) Defining Interdisciplinary Research: Conclusions from a Critical Review of the Literature. *HSR: Health Services Research* 42:1, Part I
19) Rosenfield, P.L. (1992) The Potential of Transdisciplinary Research for Sustaining and Extending Linkages Between the Health and Social Sciences. Social Science & Medicine. 35(II)
20) Chor, B.C.K., Pak, A.W.P. (2006) Multidisciplinarity, interdisciplinarity and transdisciplinarity in health research, services, education and policy: 1. Definitions, objectives, and evidence of effectiveness. *Clinical & Investigative Medicine*; 29 (6):351-364.
21) Global Forum for Health Research. (2004) The 10/90 Report on Health Research 2003-2004. Geneva: Global Forum for Health Research
22) Bates I, Boyd A, Smith H, Cole DC. (2014) A practical and systematic approach to organisational capacity strengthening for research in the health sector in Africa. *Health Research Policy and Systems* 12:11
23) MEASURE Evaluation. 2012.Fundamentals of Implementation Research.
24) Brown, R.R., Deletic, A. & Wong, T.H.F. (2015) Interdisciplinary: How to catalyse collaboration. *Nature*
25) Levac, D., Colquhoun, H. & O'Brien, K.K. (2010) Scoping studies: advancing the methodology. *Implementation Science*; 5:69.
26) McKee, M., Stuckler & D., Basu, S. (2012) Where there is no health research: what can be done to fill the global gaps in health research? *PLOS Medicine*;9: e1001209.

For peer review only - http://bmjopen.bmj.com/site/about/guidelines.xhtml

27) Gebby, K.M., Meier, B.M., Bakken, S., Carrasquillo, O., Formicola, A., Aboelela, S.W., Glied, S. & Larson, E. (2008) Training for Interdisciplinary Health Research Defining the Required Competencies. *Journal of Allied Health*, 37(2)
28) Marshall, MN. (1996) Sampling for qualitative research. *Family Practice*; 13: 522-525.

For peer review only - http://bmjopen.bmj.com/site/about/guidelines.xhtml

Manuscript title:

Actions for fostering cross-disciplinary global health research: A qualitative research

Research checklist: Standards for Reporting Qualitative Research (SRQR)[a]

No.	Topic	Item	Page no.	Notes
	**Title and abstract**			
S1	Title	Concise description of the nature and topic of the study Identifying the study as qualitative or indicating the approach (e.g., ethnography, grounded theory) or data collection methods (e.g., interview, focus group) is recommended	page (p) 1	
S2	Abstract	Summary of key elements of the study using the abstract format of the intended publication; typically includes background, purpose, methods, results, and conclusions	P2	The abstract follows a structure suggested by BMJ Open
	**Introduction**			
S3	Problem formulation	Description and significance of the problem/phenomenon studied; review of relevant theory and empirical work; problem statement	P3,4	
S4	Purpose or research question	Purpose of the study and specific objectives or questions	P4	
	**Methods**			
S5	Qualitative approach and research paradigm	Qualitative approach (e.g., ethnography, grounded theory, case study, phenomenology, narrative research) and guiding theory if appropriate; identifying the research paradigm (e.g., postpositivist, constructivist/ interpretivist) is also recommended; rationale[b]	P5-7	We adapted a framework to frame the study including data collection and analysis
S6	Researcher characteristics and reflexivity	Researchers' characteristics that may influence the research, including personal attributes, qualifications/experience, relationship with participants, assumptions, and/or presuppositions; potential or actual interaction between researchers' characteristics and the research questions, approach, methods, results, and/or transferability	P3	In "Strengths and limitations of this study" bulletin point 5, we reflected on our influence on interviewees.
S7	Context	Setting/site and salient contextual factors; rationale[b]	P4	We introduced the context and our case

For peer review only

				study programme in INTRODUCTION instead of METHODS as the context and case study helps understand our research question.
S8	Sampling strategy	How and why research participants, documents, or events were selected; criteria for deciding when no further sampling was necessary (e.g., sampling saturation); rationale[b]	P5,6	
S9	Ethical issues pertaining to human subjects	Documentation of approval by an appropriate ethics review board and participant consent, or explanation for lack thereof; other confidentiality and data security issues	P27	Information on research ethics has been provided separately from the manuscript
S10	Data collection methods	Types of data collected; details of data collection procedures including (as appropriate) start and stop dates of data collection and analysis, iterative process, triangulation of sources/methods, and modification of procedures in response to evolving study findings; rationale[b]	P5,6	
S11	Data collection instruments and technologies	Description of instruments (e.g., interview guides, questionnaires) and devices (e.g., audio recorders) used for data collection; if/how the instrument(s) changed over the course of the study	P5,6	
S12	Units of study	Number and relevant characteristics of participants, documents, or events included in the study; level of participation (could be reported in results)	P7-9	These are reported in results
S13	Data processing	Methods for processing data prior to and during analysis, including transcription, data entry, data management and security, verification of data integrity, data coding, and anonymization/deidentification of excerpts	P5,6	In "procedure of interviews", and also in each data collection method
S14	Data analysis	Process by which inferences, themes, etc., were identified and developed, including the researchers involved in data analysis; usually references a specific paradigm or approach; rationale [b]	P5	P5 on the framework for the study;
S15	Techniques to enhance	Techniques to enhance trustworthiness and credibility of data analysis (e.g.,	P3,5, 6	The credibility of our findings is strengthened from having used

For peer review only

	trustworthiness	member checking, audit trail, triangulation); rationale[b]		interview and observational data from diverse interviewees and events, corroborated by document analysis.
	**Results/findings**			
S16	Synthesis and interpretation	Main findings (e.g., interpretations, inferences, and themes); might include development of a theory or model, or integration with prior research or theory	P10-21	Ten themes emerged from the findings.
S17	Links to empirical data	Evidence (e.g., quotes, field notes, text excerpts, photographs) to substantiate analytic findings	P10-21	
	**Discussion**			
S18	Integration with prior work, implications, transferability, and contribution(s) to the field	Short summary of main findings; explanation of how findings and conclusions connect to, support, elaborate on, or challenge conclusions of earlier scholarship; discussion of scope of application/generalizability; identification of unique contribution(s) to scholarship in a discipline or field	P21-26	
S19	Limitations	Trustworthiness and limitations of findings	P3	Provided in 'Strengths and limitations of this study' as required by BMJ Open.
	**Other**			
S20	Conflicts of interest	Potential sources of influence or perceived influence on study conduct and conclusions; how these were managed	P27	A competing interests statement was provided.
S21	Funding	Sources of funding and other support; role of funders in data collection, interpretation, and reporting	P27	A funding statement was provided.

[a] O'Brien, Bridget C.; Harris, Ilene B.; Beckman, Thomas J.; Reed, Darcy A.; Cook, David A. Academic Medicine89(9):1245-1251, September 2014. doi: 10.1097/ACM.0000000000000388

The authors created the SRQR by searching the literature to identify guidelines, reporting standards, and critical appraisal criteria for qualitative research; reviewing the reference lists of retrieved sources; and contacting experts to gain feedback. The SRQR aims to improve the transparency of all aspects of qualitative research by providing clear standards for reporting qualitative research.

[b]The rationale should briefly discuss the justification for choosing that theory, approach, method, or technique rather than other options available, the assumptions and limitations implicit in those choices, and how those choices influence study conclusions and transferability. As appropriate, the rationale for several items might be discussed together.

For peer review only

BMJ Open

Identifying actions to foster cross-disciplinary global health research: a mixed-methods qualitative case study of the IMPALA programme on lung health and tuberculosis in Africa

Journal:	*BMJ Open*
Manuscript ID	bmjopen-2021-058126.R1
Article Type:	Original research
Date Submitted by the Author:	24-Jan-2022
Complete List of Authors:	Ding, Yan; Liverpool School of Tropical Medicine, Centre for Capacity Research<br>Tomeny, Ewan; Liverpool School of Tropical Medicine, Clinical Sciences<br>Bates, Imelda; Liverpool School of Tropical Medicine, Centre for Capacity Research<br>on behalf of, The IMPALA Consortium; IMPALA
**Primary Subject Heading**:	Global health
Secondary Subject Heading:	Qualitative research
Keywords:	PUBLIC HEALTH, QUALITATIVE RESEARCH, Anthropology < TROPICAL MEDICINE

SCHOLARONE™
Manuscripts

I, the Submitting Author has the right to grant and does grant on behalf of all authors of the Work (as defined in the below author licence), an exclusive licence and/or a non-exclusive licence for contributions from authors who are: i) UK Crown employees; ii) where BMJ has agreed a CC-BY licence shall apply, and/or iii) in accordance with the terms applicable for US Federal Government officers or employees acting as part of their official duties; on a worldwide, perpetual, irrevocable, royalty-free basis to BMJ Publishing Group Ltd ("BMJ") its licensees and where the relevant Journal is co-owned by BMJ to the co-owners of the Journal, to publish the Work in this journal and any other BMJ products and to exploit all rights, as set out in our licence.

The Submitting Author accepts and understands that any supply made under these terms is made by BMJ to the Submitting Author unless you are acting as an employee on behalf of your employer or a postgraduate student of an affiliated institution which is paying any applicable article publishing charge ("APC") for Open Access articles. Where the Submitting Author wishes to make the Work available on an Open Access basis (and intends to pay the relevant APC), the terms of reuse of such Open Access shall be governed by a Creative Commons licence – details of these licences and which Creative Commons licence will apply to this Work are set out in our licence referred to above.

Other than as permitted in any relevant BMJ Author's Self Archiving Policies, I confirm this Work has not been accepted for publication elsewhere, is not being considered for publication elsewhere and does not duplicate material already published. I confirm all authors consent to publication of this Work and authorise the granting of this licence.

For peer review only

Identifying actions to foster cross-disciplinary global health research: a mixed-methods qualitative case study of the IMPALA programme on lung health and tuberculosis in Africa

Yan Ding, Ewan M Tomeny, Imelda Bates, and on behalf of the IMPALA Consortium

Yan Ding[1]*Centre for Capacity Research, Liverpool School of Tropical Medicine, Liverpool, L3 5QA, UK; Yan.Ding@lstmed.ac.uk

Ewan M Tomeny[2]**,** Department of Clinical Sciences, Liverpool School of Tropical Medicine, Liverpool, L3 5QA, UK; Ewan.Tomeny@lstmed.ac.uk

Imelda Bates[1], Centre for Capacity Research, Liverpool School of Tropical Medicine, Liverpool, L3 5QA, UK; Imelda.Bates@lstmed.ac.uk

[1] Centre for Capacity Research, Liverpool School of Tropical Medicine, Pembroke Place, Liverpool, L3 5QA, UK

[2] Department of Clinical Sciences, Liverpool School of Tropical Medicine, Liverpool, L3 5QA, UK

*Corresponding author. Centre for Capacity Research, Liverpool School of Tropical Medicine, Pembroke Place, Liverpool, L3 5QA, UK
E-mail address: Yan.Ding@lstmed.ac.uk (Yan Ding)
Tel: (+44) (0)1517029316

Word count: 5510

For peer review only

For peer review only

For peer review only - http://bmjopen.bmj.com/site/about/guidelines.xhtml

Identifying actions to foster cross-disciplinary global health research: a mixed-methods qualitative case study of the IMPALA programme on lung health and tuberculosis in Africa

Abstract

Objectives: To identify actions for fostering cross-disciplinary research skills and collaborations in global health, and to produce recommendations for improving the design, implementation, and management of cross-disciplinary global health research programmes.

Design: Using a North-South global health research programme as a case study — and following an adapted framework — we conducted qualitative research using document reviews, semi-structured interviews (purposive sampling), and participatory observation. We used baseline-survey findings to identify potential interviewees and tailor interview guides.

Setting: Our case study was a 4.5-year (2017-2021) programme, namely *the International Multidisciplinary Programme to Address Lung Health and Tuberculosis in Africa* (IMPALA). Led by a UK research institute, IMPALA spanned 22 partner organisations from 13 countries (10 in sub-Saharan Africa), and involved five research discipline groups*: clinical science*, *social science, health systems*, *health economics*, and *policy/research capacity*.

Participants: Thirty-one IMPALA members were interviewed (Jul.2018-Nov.2019), with interviewees evenly split by sex (16F; 15M) and by Global North/South institution (15 UK; 16

For peer review only - http://bmjopen.bmj.com/site/about/guidelines.xhtml

Africa). Twenty-five (81%) were researchers, comprising 18 senior researchers (professors, readers, associate professors, senior lecturers) and 7 early career researchers (assistant professors, lecturers, research fellows, post-docs, research assistants, PhD students). Twenty-four programme events were observed (Sept.2018–Apr.2020) and 49 documents were reviewed (Dec.2017–Apr.2020). All 66 IMPALA staff were sent the baseline survey, receiving 51 responses (43/56 researchers; 8/10 non-researchers).

Results: Fourteen themes emerged, which suggested that cross-disciplinary research — while valued by many — is not universally understood, and the time it requires often underestimated. We found that fostering cross-disciplinary research and managing tensions needs planning and continuous discussions and interactions. A shared vision with explicitly agreed goals and roles, and active management of cross-disciplinary activities is essential.

Conclusions: Active planning, implementation, and management of cross-disciplinary activities are essential for the success of cross-disciplinary global health research, and should be separate from the primary research activities.

Strengths and limitations of this study

- We used an adapted published framework and a recent literature review to frame our data collection tools and analysis, and have placed our findings in the context of current global knowledge concerning cross-disciplinary research.

For peer review only

- The credibility of our findings is strengthened from having used interview and observational data from diverse interviewees and events, corroborated by document analysis.
- Our study focused on a single cross-disciplinary global health research programme and its projects.
- We have enhanced the transferability of our findings by describing the complexity of the programme and the context within which the cross-disciplinary research took place.
- Our role as IMPALA members in conducting research on cross-disciplinary working in IMPALA may affect interviewees' responses which we mitigated by ensuring confidentiality.

For peer review only

For peer review only - http://bmjopen.bmj.com/site/about/guidelines.xhtml

Main text

INTRODUCTION

Bringing together researchers from multiple disciplines can lead to innovation and rapid production and dissemination of cross-disciplinary knowledge to solve complex global health problems.[1, 2] Cross-disciplinary research has been growing globally in popularity among researchers and funders because of its importance in addressing global health challenges.[1, 2] 'Cross-disciplinary research' covers three typologies: multi-, inter-, and trans-disciplinary research. In this article we will use the term cross-disciplinary research (CDR) to mean research that combines concepts, methods, and theories drawn from two or more disciplines.[3]

Existing evidence on fostering CDR is fragmented across disciplines,[4, 5] making it difficult to find. There is increasing interest in understanding how to implement effective CDR and in the importance of team dynamics between researchers from disparate disciplines. CDR tends to be more complex than traditional types of research,[6] and presents unique challenges,[1, 3] such as problem definition, positioning in different disciplines[7] and coordination of effort.[8, 9] Our previous literature review found that evidence about how to conduct effective CDR is primarily from high-income countries, and may not apply to CDR in global health, where research is typically conducted through North-South collaborations.[3]

We used the *International Multidisciplinary Programme to Address Lung Health and Tuberculosis in Africa* (IMPALA 2017-21))[10] as a case study to explore and reflect on practical actions for fostering CDR in North-South collaborations. IMPALA aimed to generate knowledge and implementable solutions concerning lung health and tuberculosis. Led by a Global North research institute, IMPALA had 22 international partner organisations from 13 countries, 10 in sub-Saharan Africa.

For peer review only - http://bmjopen.bmj.com/site/about/guidelines.xhtml

IMPALA explicitly used multidisciplinary approaches and spanned biology to policy[11]. It involved five research disciplines: *clinical science*, *social sciences*, *health systems*, *health economics*, and *policy/research capacity*. Unusually, to promote fairness and overcome disciplinary hierarchies, the programme was framed around these discipline groups: each group initially received the same amount of funding and was represented on the management team alongside the three consortium directors. Each group had one PhD student and one postdoctoral researcher (Figure 1), with equal training opportunities offered to all early career researchers (ECRs).

[Insert Figure 1: The IMPALA Organogram]

Our study has drawn on IMPALA as a whole, and its two embedded projects (hereafter '*the two projects'*): one combined *clinical sciences* and *health economics*; the other *health systems* and *social sciences* This qualitative study explores the actions taken to foster CDR in the 'real life' situation of a large programme (IMPALA). Our aim is to recommend actions that can be used to improve the effectiveness of future global health CDR programmes.

METHODS

We adapted a previously published model of CDR collaborations, the 'Four-Phase Model of Transdisciplinary Research' (Figure 2)[9] which describes objectives within each project phase (i.e., *Development*, *Conceptualisation*, *Implementation*, *Translation*). We combined the *Development* and *Conceptualisation* phases into one 'Planning' phase, since global health research activities in these phases are generally integrated.[12] The *Translation* phase was not included because it requires long-term follow-up. Our literature review indicated that leadership and management strongly influence CDR effectiveness,[3] so these were added as a cross-cutting framework component.

[Insert Figure 2: The three-component framework for the cross disciplinary collaborative research process used in this study (adapted from Hall et al., 2012)]

For peer review only - http://bmjopen.bmj.com/site/about/guidelines.xhtml

Data collection

Our primary source of data was semi-structured interviews, supplemented by a baseline survey, a document review, and observations of events.

Baseline survey

All IMPALA members were invited to complete a baseline survey (May-September 2018). This included individuals from the External Scientific Advisory Panel, Leadership and Management Teams, Administrators, and researchers/policy makers involved in the two projects. Participants were emailed an information sheet (Supplementary file 1) prior to beginning the online survey, and agreement to participate confirmed by the signing of an online consent form (Supplementary file 2). The survey (Supplementary file 3) collected participants' personal information, and their experience of, and confidence in, conducting CDR. The survey findings were used to identify potential interviewees and tailor interview guides.

Semi-structured interviews

The interviews collected data on challenges and practical actions/solutions related to the fostering and conducting of CDR in IMPALA.

Sample selection

Guided by our baseline survey data 31 primary interviewees were selected using the IMPALA team directory as a sampling frame. Purposive sampling was used to maximise variation in roles, disciplinary backgrounds, career stages, gender, affiliated organisations, and geographical locations, in order to achieve maximum variation in participants' characteristics.[13] These characteristics were primarily collected from baseline survey findings.

Procedure of interviews

For peer review only

For peer review only - http://bmjopen.bmj.com/site/about/guidelines.xhtml

YD, an experienced social scientist with substantial experience of research interviews, carried out the interviews between July 2018 and November 2019. Interview questions (Supplementary file 4) were based on the adapted framework (Figure 2) with probes informed by our literature review.[3] The interviewer asked neutral and open-ended questions without assumptions. Interviews were audio recorded and conducted in English, either in-person or virtually. Participants were sent a project information sheet (Supplementary file 5) before their interview, with informed consent (Supplementary file 6) obtained in writing before each in-person interview and via email for Skype interviews. For anonymity each interviewee was assigned an ID.

Reflexivity throughout the interview process

We used reflexivity throughout the interview process to improve the rigour of the data collection. We acknowledged that our role as IMPALA members in conducting research on cross-disciplinary working in IMPALA may have affected interviewees' responses. We attempted to mitigate this bias by reassuring participants of strict confidentiality, and that our findings would be unidentifiable when reported. The interviewer transcribed the first four interviews to familiarise herself with the data and to reflect on the interview process for further improvement. The interviewer also had several debriefing meetings with IB — the senior researcher — reflecting on how to further improve interviews and on data analysis.

<u>Document review</u>

Data were extracted from documents concerning the programme's: *vision*, *goals*, *research questions*, *design*, *teams*, *interactions*, and *outputs,* to understand the context of the programme and its projects, inform interview questions, and cross-check findings from other data-collection methods. Documents included the IMPALA website, concept notes, proposals, minutes/agendas from annual meetings, and quarterly research updates.

<u>Observation of events</u>

For peer review only

YD was a participant observer at IMPALA events involving cross-disciplinary issues including two annual meetings, monthly knowledge exchange meetings, training workshops, and a 4-day field visit to Tanzania. After receiving oral consent from event participants, observation notes were entered in real time into a pre-designed form (Supplementary file 7) informed by the literature,[14] comprising sections on: brainstorming the crossing of analytical levels; integration of disciplinary ideas; proposed/actual cross-disciplinary outcomes; information sharing; technical or emotional support; and challenges and setbacks.[14] Observation findings were used for refining interview questions and triangulating interview data.

Data analysis

Interview data were coded, mapped, and analysed using the framework (Figure 2) with narrative summaries created through a combined inductive and deductive approach. This method used thematic synthesis through a ‘constant comparison’ method,[15] wherein themes and sub-themes were identified throughout the coding, which were then adjusted iteratively by constantly comparing among them through reflection and analyses. In this way the themes and sub-themes are refined and integrated to form the basis of a coherent and explanatory descriptive narrative. Information from the document review and the observation forms that related to the narrative themes were summarised and compared with these narrative themes to triangulate the findings.

Patient and public involvement

While the IMPALA programme involved both patients and the public, due to this study’s specific focus on research practice, its design, conduct, and reporting did not involve patients or the public.

RESULTS

Interviewee characteristics

For peer review only

Thirty-six interviews with 31 interviewees were conducted each lasting 68-192 minutes. Five individuals were interviewed again after one year to identify changes in CDR in the two projects. 52% of interviewees (16/31) were female, and 16 were based at African organisations from 7/10 partner African countries. Twenty-five (81%) were researchers, comprising 18 senior researchers (i.e., professors, readers, associate professors, senior lecturers) and 7 ECRs (i.e., assistant professors, lecturers, research fellows, post-docs, research assistants, PhD students) (Table 1).

Table 1: Interviewees' characteristics

	**Items**	**Option**	**N**
1	Role in IMPALA (Options not mutually exclusive)	A member of the External Scientific Advisory Panel	2
		A member of the Leadership Team	4
		A member of the Management Team	8
		A member of the Management & Administration Support Team	3
		Other member working across IMPALA projects	2
		A researcher or policy maker on the two projects;	15
		(of these those based in Africa)	(8)
		An IMPALA member who was based in Africa, but not on the two projects	5
2	Sex	Female	16
		Male	15
3	Location	African Countries	16
		United Kingdom	15
4	Primary Disciplinary Background	Medicine & Clinical Sciences	18
		Humanities & Social Sciences	10
		Others	3
5	Profession	Researchers/research leaders	25

For peer review only

For peer review only - http://bmjopen.bmj.com/site/about/guidelines.xhtml

	Items	Option	N
		Non-research members	6
6	Academic Rank (of the 25 researchers)	Senior researcher	18
		Early career researcher	7

198 ## Survey, document reviews and observations

199 The baseline survey was sent to 66 IMPALA staff, with responses received from 43/56 researchers
200 (77%), and 8/10 non-researchers (80%). Twenty-four events were observed over 20 months (Sept. 18
201 – Apr. 20) and 49 documents were reviewed (Box 1).

For peer review only

For peer review only - http://bmjopen.bmj.com/site/about/guidelines.xhtml

Box 1: Internal IMPALA documents used to provide background information for this study

1. The IMPALA website: https://www.lstmed.ac.uk/impala
2. IMPALA Technical Proposal
3. IMPALA Team Directory
4. The concept notes of all the eight research projects sitting under IMPALA
5. IMPALA Publication Guidelines
6. IMPALA Data sharing, Access and Release Policy
7. IMPALA Data Management Guidelines
8. IMPALA Communications Plan
9. IMPALA kick-off meeting in 2017, annual meetings in 2018 and 2019, including
    1) Meeting schedule
    2) Attendees list and biographies
    3) Meeting slides
10. IMPALA technical reports, including
    1) IMPALA 2017 report (covering the first six months of IMPALA)
    2) IMPALA 2018 annual technical report
    3) IMPALA 2019 annual technical report
11. Research ethics application documents of the two case study projects, including research proposals and data collection tools
12. Quarterly updates by the four post-doctoral researchers each on the two post-doctoral researchers led projects (August 2018 - December 2019, 20 documented updates in total)
13. IMPALA Year 1-3 Joint Outputs list

Research results

Fourteen themes emerged from the findings, 5 for Planning, 3 for Implementation, and 6 for leadership and management (Box 2). Interviewee's anonymised quotes are presented with their main role (researcher/non-researcher) and location (Africa/non-Africa).

Box 2: Summary of the 14 themes which emerged from the findings

Five themes for Planning Phase

1. Shared vision and goals
2. Expectations of programme-level goals and success
3. Shared understanding of research questions and activities at the project level
4. Reasons for using CDR in IMPALA
5. Cross-disciplinary orientation

Three themes for Implementation Phase

1. Shared understanding of roles and responsibilities
2. Reconciling individual expectations while navigating different contexts
3. Team learning

Six themes for Management and Leadership Component

1. Communication planning and implementation
2. Nurturing trust and a group environment of psychological safety
3. Addressing disciplinary hierarchies through the management structure
4. Handling disciplinary differences and managing emotions
5. Developing research networks for possible future collaborations
6. Strengthening capacity

Actions that fostered CDR in the Planning Phase

Shared vision and goals

Interviewees identified that co-development of the IMPALA proposal between members from the Global South and North helped them reach a common vision. While this was time-consuming due to the large number of cross-disciplinary, inter-organisational, and geographically distanced members, several factors helped the process including the existence of previous/ongoing collaborations and involvement in professional associations.

During the face-to-face start-up meeting, IMPALA members and the 22 participating institutions introduced themselves and IMPALA's vision and strategic objectives were discussed. Specific goals for projects —and for IMPALA as a whole— had purposefully been left undefined by the management team so they could be co-developed during this meeting. Interviewees reported finding this meeting useful for grasping the 'bigger picture' of IMPALA, and for learning about one other.

Expectations of programme-level goals and success

Interviewees had different expectations of IMPALA depending on their seniority and disciplinary background. Senior, clinical researchers tended to focus on the need to expand collaborations with partners. Two interviewees suggested that since many senior researchers had clinical sciences backgrounds, IMPALA provided more opportunities for clinical researchers to expand collaborations, compared to other programmes. Interviewees from non-clinical disciplines (e.g., social sciences and health systems) were more focused on their existing projects and research quality. Senior researchers sought to enhance ECR's research skills, and ECRs were focussed on generating outputs and building working relationships. Non-researchers focussed on programme delivery, and capacity strengthening in areas such as financial management, leadership, and policy engagement. All interviewees reported expecting IMPALA to lead to new research questions and new funding. Observation data confirmed all these findings.

For peer review only - http://bmjopen.bmj.com/site/about/guidelines.xhtml

Interviewees recognised the complexity of aligning project and programme goals. Two interviewees acknowledged the difficulty of collective prioritisation, and proposed mapping the connections between programme and project objectives, possibly annually. One participant stated that although seeking clarity around programme goals can facilitate members' engagement, balancing partnership development against addressing a large-scale broad research question with multiple disciplines is difficult.

Shared understanding of research questions and activities at the project level

IMPALA's proposal outlined broad topics for research projects —with named leads and partners for each— while leaving specific research questions and activities to be developed during the start-up meeting. Project leads recognised that this allowed research questions to be based on the interests and experience of partners, and some expressed appreciation that programme leaders had not imposed personal priorities.

Researchers from both Global North and South were comfortable with this process, with Global South partners feeling they had driven the research agenda:

> *"I was looking at ways how I can also contribute rather than just passively engage in national meetings … we were there to conceptualise...what we want to do, … we got the research budget." (ID-21, researcher, Africa)*

Others noted a risk of mismatch between programme and project goals, and had difficulty narrowing research questions down from programme to project level:

> *"When you have …. multiple perspectives that lead to such a broad potential for research questions that narrowing down and getting in some consensus can be quite difficult." (ID-1, researcher, Africa)*

The two project teams addressed this differently:

For peer review only - http://bmjopen.bmj.com/site/about/guidelines.xhtml

One developed research questions based on a baseline assessment conducted during joint field trips with local research and implementation teams, enabling them to develop locally important, high-priority research questions. To address these questions, they drew on methods from their two core disciplines, indicating some complementarity in their disciplinary paradigms such as theories (e.g. pragmatic health systems thinking, community engagement and empowerment), research methods (e.g. quantitative research methods for health systems data, qualitative research methods to understand the quantitative data further, participatory action research approaches), and standards (e.g. pragmatic and efficiency, local ownership, feasibility and acceptability, and sustainability). The benefits of having one project integrating two disciplinary components appeared clear to this team from the outset.

The other project team initially generated their research questions independently within each of their two disciplines and then merged the projects through discussions and negotiation which was "*initially uncomfortable" (ID-13, researcher, non-Africa).* One researcher believed "*practical efficiency in terms of time and data collection*" *(ID-9, researcher, Africa)* of this approach to have been the main advantage of merging the two disciplinary research projects into one.

Reasons for using CDR in IMPALA

IMPALA took a CDR approach as it was felt its broad research question – i.e. *to address lung health and tuberculosis in Africa* – required inputs from multiple disciplines and programme leaders recognised that everyone had a role in ensuring research findings informed policy. Interviewees considered CDR as one of the "*most effective ways to generate the best possible outputs and outcomes" (ID-13, researcher, non-Africa)* since it "*enables appropriate generalisation of research outcomes*" *(ID-15, researcher, non-Africa)*. Several interviewees mentioned that multidisciplinary research was a funder's requirement; however one cautioned *"don't just do* [CDR] *for the sake of it" (ID-14, researcher, non-Africa)*.

For peer review only - http://bmjopen.bmj.com/site/about/guidelines.xhtml

While most senior researchers recognised the importance of CDR, most interviewees (researchers and non-researchers) had not participated in explicit discussions on what actions would be needed to conduct CDR.

> *"A lot of the challenges is people are so busy doing their own things that they forget that that is what needs to happen." (ID-12, researcher, non-Africa)*

The IMPALA programme included a post-doctoral researcher (YD) dedicated to investigating cross-disciplinary working. The definitions of multi-, inter- and cross-disciplinary research were presented to IMPALA members during the second IMPALA annual meeting prompting discussions and clarifications. However, interview findings suggest such clarifications would have been useful earlier, alongside discussions on pre-specified goals/methodologies concerning cross-disciplinary working.

Cross-disciplinary orientation

Observations clearly indicated that IMPALA members valued understanding more about each other and their disciplines especially within a group environment of psychological safety, while highlighting the value of clarifying disciplinary boundaries to prevent conflicts.

Having inputs from colleagues with various disciplinary backgrounds at the planning phase and arranging formal time for candid conversations on research questions and design were viewed by interviewees as critical. A programme leader and a researcher highlighted potential tensions in cross-disciplinary working, and the need for maintaining *'discipline uniqueness'*. The process of defining and clarifying research goals among disciplines was considered to have helped clarify disciplinary boundaries:

> *"After the goals are fixed and then each goal somehow belongs to certain disciplines...relate data to that goal and then deal with the data, publication, all those things followed." (ID-15, researcher, non-Africa)*

For peer review only - http://bmjopen.bmj.com/site/about/guidelines.xhtml

Actions that fostered CDR in the Implementation Phase

Shared understanding of roles and responsibilities

Collaborative working was facilitated by a shared understanding of the roles and contributions of different disciplines and partners, along with an appreciation that successful cross-disciplinary collaborations require complementarity rather than competition. This helped team members to overcome *'fighting for space'* and *'struggling for context leadership'* (ID-22, researcher, Africa). Several interviewees noted the importance of research administrators in helping to understand responsibilities.

> *"Because we [administrators] are that sort of hub in the middle, and we do oversee everything. We can sort of speak on behalf of the project and say that this isn't working and have a bit of input in that way." (ID-3, non-researcher, non-Africa)*

Several interviewees had not had open discussions about roles and responsibilities, with one suggesting that roles were defined by one's job description and another explaining that *"as a member of the team you naturally know your strengths and therefore role" (ID-5, researcher, Africa).* Another interviewee highlighted that assumptions regarding roles and responsibilities had the potential to cause confusion and needed open discussions:

> *"I increasingly think the best way to have good, harmonious, collaborative relationships is to be really upfront about roles and responsibilities. To do that first so that there is no confusion after." (ID-9, researcher, Africa)*

One interviewee suggested that jointly developing a work plan, containing explanations of responsibilities alongside a clear timeline could help to clarify roles.

Reconciling individual expectations while navigating different contexts

For peer review only - http://bmjopen.bmj.com/site/about/guidelines.xhtml

Several interviewees advocated for open discussions on roles, suggesting such discussions were important because people were at different career stages with different experiences, cultures and academic systems, which could cause mismatched expectations of one another's roles. This led to, for example, disagreements on the time spent in the research sites and responsibilities for research coordination. Clarifying roles and having a host country/institution coordinator was thought to be essential in avoiding these issues.

Regular cross-disciplinary project update meetings along with individual conversations to provide performance feedback to ECRs (including those with different disciplinary backgrounds) were said to be useful by both ECRs and senior researchers. Role modelling was also identified as important in encouraging ECRs to continuously explore other disciplines:

> *"Seniors and line managers say, 'You should go to this. Think about this...' So, it does need people, at a senior level, to think broadly and encourage that." (ID-23, researcher, non-Africa)*

Support across disciplines was valued during project implementation, for example when developing questionnaires, collecting and analysing data and several senior researchers called for more thought on how to provide supportive supervision:

> *"Perhaps we didn't think hard enough about how to support the projects and who should be supporting the projects and in what way." (ID-9, researcher, Africa)*

Team learning

The importance of individuals' ability to blend disciplinary edges was raised by an interviewee and many others shared their approaches to understanding other disciplines. Senior researchers also encouraged colleagues to consider broadening the scope of their work and skillset through formal cross-disciplinary training, mutual learning, and joint supervision in other subject areas. One month after the interviews, monthly knowledge exchange meetings were initiated to improve cross-

For peer review only - http://bmjopen.bmj.com/site/about/guidelines.xhtml

disciplinary learning and communication, according to our observation and review of programme documents.

Leadership and Management

Communication planning and implementation

New IMPALA members appreciated their one-to-one induction meetings with key researchers and administrators. Joint site visits by members from the Global North and South were helpful in forming relationships and in promoting cross-fertilisation. Face-to-face meetings were valued for facilitating the design, prioritisation, and development of both research projects and teams, especially concerning developing methods and budgeting. Interviewees said that virtual meetings and email communications worked well and were useful, though several raised issues with internet connections. Effective planning to maximise the availability of team members was highlighted:

> *"What I usually do is to inform them early enough because they have lots of responsibilities…After they have considered then you block the time... With multi-disciplinary, it needs proper planning, especially on timing." (ID-26, researcher, Africa)*

Many senior researchers often had long working relationships with country partners. To help ECRs to build mutual understanding and develop research networks, regional meetings for ECRs across disciplines were suggested.

Several interviewees suggested that having access to other teams' materials and outputs could have improved cross-disciplinary understanding. A common platform for document and information sharing was subsequently established. Interviewees further proposed that cross-disciplinary communications should be expanded. Accordingly, the monthly knowledge exchange meetings were expanded beyond ECRs to include administrative staff, in-country partners, and researchers beyond IMPALA's core team.

For peer review only - http://bmjopen.bmj.com/site/about/guidelines.xhtml

Interviewees wanted more time to develop mutual understanding in CDR and to create a sense of ownership. One interviewee reflected *"we need to have some more recognition of the need for time for some of the processes and the collaborations to work for the future" (ID-11, researcher, non-Africa).* Another recommended taking time to learn about each other's experiences and expectations, ways to successfully collaborate, and for joint preparation of project tools (e.g. databases).

According to several interviewees *"there are inevitable delays in starting" (ID-9, researcher, Africa) for example* in funding release (6 months), international staff recruitment (5-8 months) and ethics approval (7-8 months). Interviewees described how they felt the need to focus on outputs although *"would have loved to have used those six months to think about how we prepare these disciplines to work together" (ID-11, researcher, non-Africa).* One interviewee highlighted the importance of prioritising internal communication even within tight timescales, arguing *"sometimes prioritising a two-hour meeting to make sure everyone's on the same page and understanding things in the same way is equally important as papers and research outputs" (ID-11, researcher, non-Africa).*

Nurturing trust and a group environment of psychological safety

Two senior researchers, three ECRs and three non-researchers noted that IMPALA management had helpfully promoted involvement and empowerment of ECRs and non-researchers and two ECRs appreciated the space and freedom their line managers had given them to lead projects.

There were three other suggestions offered by interviewees for nurturing trust:

i) Treating everyone equally through *"flat management"*:

> *"I very strongly believe in flat management, a structure everybody is equal. If I have a research meeting in my team, they all know we are equal. If they have something to say, they are all happy to say, and confident to say it." (ID-2, researcher, non-Africa)*

ii) Building trust by delivering on commitments (mentioned by two researchers and one non-researcher):

For peer review only

For peer review only - http://bmjopen.bmj.com/site/about/guidelines.xhtml

"To build trust you need to deliver... I think that's important, showing that you want to do your best. Then by reflection they don't want to let me down, so they deliver, and that's how you build trust, I think." (ID-4, non-researcher, non-Africa)

iii) Being transparent and learning from mistakes.

"Transparent, I think building trust… Also within trust and team, you have to allow mistakes… Accepting and also sitting together and see how we can handle it next time." (ID-21, researcher, Africa)

Addressing disciplinary hierarchies through the management structure

According to three interviewees, disciplinary hierarchies emerged when one discipline's work depended on another. For example, when one discipline's research questions and analysis relied on another's data generation, the latter may perceive their research activities should be prioritised over the former. Despite both projects having been allocated equivalent resources at the start of IMPALA, perceived imbalances arose. Five interviewees suggested that since clinical aspects were the primary interest of several IMPALA leaders, this may have inadvertently contributed to disciplinary hierarchies. Furthermore, several interviewees found the equal allocation of resources limiting, potentially hindering the effective answering of some research questions. Two interviewees further noted that since studies were highly interconnected at the operational level, strict drawing of financial boundaries between projects could at times *"lead to tensions" (ID-1, researcher, Africa).*

Following the initial equal allocation of resources, a degree of re-negotiation continued throughout IMPALA's lifetime though some members questioned the success of this process. One remarked that *"an alternative approach may be to develop the budget based on justified activities" (ID-15, researcher, non-Africa).*

Handling disciplinary differences and managing emotions

For peer review only

At times, the different approaches and priorities of disciplines led to some disagreements. Overall, the group which combined *Clinical Sciences* and *Health Economics* was perceived as predominantly outputs-driven whereas the *Humanities and Social Sciences group* appeared primarily focussed on processes, consultation, and discussions. We observed frustration within cross-team project meetings and programme management meetings particularly in the first year of the programme; this observed frustration was confirmed in several interviews. One interviewee from the Management Team reflected *"we probably hadn't paid enough attention to the need for the process [of discussions between the management team members]"* because it "*requires sustained effort to balance the natural priority of an individual's discipline against that of multiple disciplines*" *(ID-11, researcher, non-Africa)*. Two interviewees suggested that time spent discussing managerial and logistics issues could have been more productively spent on research activities and constructive management of disciplinary disagreements.

Several interviewees described encountering emotional challenges, most frequently caused by disciplinary differences and some identified having needed for dedicated meetings to manage emotions in a professional environment. One interviewee commented that their previous working relationships and sense of responsibility had helped to make these conversations possible.

Such conversations resulted in real-time adaptations to the programme to enhance cross-disciplinary relationships. For example, monthly Directorate and Management Team meetings were merged and a rotational system for the management meeting chair was instigated whereby each discipline lead and the consortium directors took turns in chairing. Handovers between meetings were supported by the Programme Management and Administration support staff. Actions to promote more effective cross-disciplinary collaborations were also identified through a one-hour consortium level group exercise during the second annual meeting. This meeting was led by our research group on fostering CDR, and included small-group discussions with consortium members from a mix of disciplines, seniority, organisations, and research teams. These actions were documented through a report with

For peer review only - http://bmjopen.bmj.com/site/about/guidelines.xhtml

feedback from consortium members. Reviewing uptake of these actions became a standing item at management meetings. These changes were viewed as positive by several interviewees.

Developing research networks for possible future collaborations

Interviewees emphasised the programme's many good working relationships between different partners across Global North and South and noted the considerable benefit from strong previous relationships of key leaders. The importance of enabling the development of such research networks was a repeated theme from interviews.

> *"I think it [IMPALA] has really done a great job bringing great collaborators in terms of Africa with Liverpool, countries that are involved. I think it's really an interesting network and it has brought us together, many collaborators. People have never even met." (ID-21, researcher, Africa)*

Two interviewees reflected that project activities had helped build up trust and develop research networks.

> *"I hope my work [...] will let them [current IMPALA members] say that 'he would actually put the neck on the line and physically help you. Get him on board.'" (ID-2, researcher, non-Africa)*

Strengthening capacity

Several approaches to capacity strengthening were identified through interviews and corroborated by internal documents. These included:

- Training workshops for those with different disciplinary backgrounds from the training subjects (e.g., training on social science research methodologies, policy engagement, statistics, and spirometry)
- Coaching through team meetings and one-to-one discussions (e.g., two interviewees emphasised that discussions with a statistician catalysed research)

For peer review only - http://bmjopen.bmj.com/site/about/guidelines.xhtml

- Mentoring ECRs and providing them with platforms at high-level international meetings (e.g., the UN General Assembly)
- Learning through peer support and reflection was mentioned by ECRs, senior researchers, and non-researchers:

> *"I feel like I'm definitely learning a lot… It's nice working so closely with […] and she's able to delegate things to me as and when they come up." (ID-3, non-researcher, non-Africa)*

Capacity strengthening also involved administration and field teams:

> *"My ideal world would be a world where everyone can do it because that's capacity building in-country. And it is not just the research, it's the admins." (ID-4, non-researcher, non-Africa)*

DISCUSSION

We adapted and expanded a published framework to underpin our research. Our findings emphasise that CDR programmes require careful planning, implementing, and managing and we have identified actions to promote CDR including some that have not previously been published.

Actions in programme planning to foster CDR

Clarity in defining 'Cross-disciplinary Research'

Similar to other studies we found a lack of agreement on defining multi-, inter- and trans-disciplinary research.[16] Our findings demonstrate that explicit discussions concerning both these definitions and what CDR means in practice are critical in the Planning Phase.

Managing expectations and harmonising goals

Participants had different expectations about being involved in CDR and highlighted the importance of negotiating a clear shared vision, taking into consideration individuals' expectations.[17] To

For peer review only - http://bmjopen.bmj.com/site/about/guidelines.xhtml

harmonise goals, frequent discussions and interactions such as information sharing can be helpful, [3, 18] and need to be more frequent and intensive than in mono-discipline research.[9] Our findings shed light on tensions that can arise early in CDR, including balancing flexibility and acceptance that not all aspects of the research could be initially 'nailed down', with developing a common understanding of the goals.

As with previous studies, IMPALA's participants recognised the importance of a common conceptual framework for outlining the vision, objectives and organisational structure for showing the contributions of each discipline,[19] and to guide collaborations.[17, 20] Furthermore, evidence suggests that to have explicit knowledge integration goals for CDR is helpful.[20, 21] IMPALA's conceptual framework was strengthened during the programme, for example by taking account of local contexts (achieved through joint field trips and discussions), by co-developing research questions and by drawing methods from relevant disciplines.

Actions in programme implementation to foster CDR

Our findings reflect previous studies which suggest that cross-disciplinary relationships flourish if they are prospectively planned and actively monitored.[3, 9] This is best managed separately from activities that focus on research outputs since fostering cross-disciplinary relationships requires its own planning and activities,[22] specific monitoring indicators and mechanisms for collecting data against the indicators.[23]

Management actions to foster CDR

Development of research collaborations and networks

Our study revealed important findings concerning management strategies for encouraging equitable partnerships, fostering CDR and reconciling individual expectations. These included involving northern

For peer review only - http://bmjopen.bmj.com/site/about/guidelines.xhtml

and southern partners in co-developing a shared vision and goals, designing project-level research questions and activities, and strengthening capacity in line with a baseline capacity assessment.

Allowing time to promote cross-disciplinary activities

Our research also identified that researchers lacked sufficient time to successfully engage in discussions and processes to promote cross-disciplinary activities. Building in adequate time and funds for this throughout the programme is critical and may necessitate a shift in research planning as well as an understanding among research funders that such allocations are essential. Areas which could have benefited the most from additional time-investment included: the development of shared vision and goals; having inputs from colleagues with various disciplinary backgrounds at the planning phase; arranging formal time for candid conversations on research questions and design; development of mutual understanding; and a better understanding the processes of collaboration.

Lack of time for active consideration and management of activities to promote cross-disciplinary working is closely linked to lack of effective communication among programme members to bridge across disciplines.[24, 25] While less of a consideration in mono-disciplinary research, cross-disciplinary researchers must build mutual understanding and discuss acceptable ways forward.[26-29] Differences across disciplines can be vast, and include: philosophical;[25, 30, 31] measurement standards;[26] framing of concepts;[32] attitudes to theory and practice;[26] the use and understanding of terminology;[24, 25, 30] and expectations of communication and etiquette.[24, 26] Interviewees proposed that cross-disciplinary communications should include all team members. This requires an agreed internal communication plan, administrative support and an electronic communication platform. Other studies have also highlighted the importance of an accessible space to document programme work and decision making.[33]

For peer review only - http://bmjopen.bmj.com/site/about/guidelines.xhtml

Programme adaptations to address hierarchies and tensions

Our framework specifically recognised 'nurturing trust and a group environment of psychological safety', 'communication planning and implementation', and 'team learning' in CDR as important because of possible emotional issues associated with ownership, territoriality, academic and discipline hierarchy, and disciplinary differences. Similar to previous studies, our findings identified CDR-related emotional issues (particularly around power and hierarchy) and disagreements in disciplinary approaches.[17, 34, 35] IMPALA took measure to mitigate such frictions, including providing equal funding and training opportunities, and by adjusting the programme's management structure. These included merging monthly Directorate and Management Team meetings, having a rotating Chair for Management Team meetings, initiating monthly knowledge exchange meetings for mutual learning and cross-disciplinary communications, and creating a common platform for document and information sharing. In addition, emerging findings from our study on CDR were presented at Management Team meetings and summarised in quarterly bulletins for all IMPALA members so that they could inform subsequent programmes.

For peer review only

Strengths and limitations of the study

While our research team were not involved in decision-making at the programme and project levels, as members of IMPALA we had ongoing access to programme colleagues and documents, along with frequent opportunities for informal discussions. Nevertheless, we were conscious throughout that the conducting of our real-time investigation into the process of cross-disciplinary research within IMPALA may have influenced interviewees' responses. We therefore ensured that our interviewees understood that their participation was voluntary, that data would be handled confidentially, and that our findings would be reported anonymously. To enhance trustworthiness, we used maximum variation sampling to enhance representation of the study population and saturation was achieved. The credibility of our findings is strengthened by having used multiple research methods, and by gaining multiple perspectives, which included research and administrative staff and non-academic partners across organisations and countries.[36] Although our study focused on a single cross-disciplinary global health research programme and its embedded projects, we have enhanced the generalisability of our findings by describing the complexity of the programme and the context within which the cross-disciplinary research took place.

The three-component framework on CDR

Using our 'real-life' findings we adapted and expanded a published model of cross-disciplinary collaborative research processes[9] to create a framework useful for collecting and analysing multi-source and multi-perspective data on CDR in real-time. A new component of the framework emphasised the importance of leadership and management in CDR processes. We would recommend further adaptations to the framework to include a rationale for the components and to expand the 'shared understanding of who knows what, who does what, and how things get done'[9]. In addition to being useful for future research on CDR, our framework could be used to guide the design of cross-

For peer review only - http://bmjopen.bmj.com/site/about/guidelines.xhtml

disciplinary programmes since it has practical applications across the three programme components of planning, implementation and management (Figure 2).

Recommendations

Based on the findings from our study, our adapted framework and our knowledge of the current literature, we have developed recommendations for planning and implementing future CDR in global health to improve the effectiveness of CDR processes from the outset (Table 2).

Table 2: Recommendations for the planning, implementation and management of cross-disciplinary global health research

Research Phase	Recommendations
Planning Phase	1) Allocate adequate time to develop a shared vision and goals, including<br>a. Co-designing of programme goals;<br>b. Aligning individuals' expectations and projects' aims with the programme-level goals;<br>c. Involving all partners in proposal development, maintaining flexibility, considering individual interests and disciplines;<br>d. Justifying and communicating the cross-disciplinary approaches to be adopted and reflecting cross-disciplinary processes in an action plan;<br>e. Developing and communicating a shared understanding of the roles, responsibilities and potential contribution of disciplines and partners.<br>2) Negotiate disciplinary boundaries when necessary;<br>3) Assess and strengthen in-country teams' capacity in cross-disciplinary research, and maintain clear plans for the involvement of in-country teams in decision making processes.

For peer review only

Implementation Phase	1) Jointly develop and pre-agree on internal approaches of working across disciplines, including communication, data access and management, publication policy and credit allocation;<br>2) Track the implementation of cross-disciplinary processes with pre-agreed indicators and review and respond accordingly.
Leadership and management	1) Rotate chairs for programme management meetings to ensure prominence of all relevant disciplines and with a process for handover and preparation between meetings.<br>2) Define and agree transparent programme-level mechanisms for strategic decision making;<br>3) Develop a programme-level leadership and management plan to deliver the cross-disciplinary outputs and outcomes, including regular review of tracking indicators;<br>4) Agree roles and responsibilities, and accountabilities, and communicate these clearly to all programme members, making it explicit that every role is important in cross-disciplinary research (i.e. not just researchers).<br>5) Support an open culture of raising concerns and putting mechanisms in place for requesting support and responding to requests<br>6) Establish mechanisms for early identification of tensions, and for reflecting on and flexibly resolving differences and conflicts<br>7) Provide opportunities for joint learning and knowledge exchange across disciplinary boundaries especially methods and approaches (e.g., monthly knowledge exchange meetings)<br>8) Identify a platform for joint sharing and updating of documents.

579

For peer review only - http://bmjopen.bmj.com/site/about/guidelines.xhtml

Acknowledgments

We are grateful to all participants who agreed to take part in this case study and provided their valuable information and insight. We are also thankful to the IMPALA central administration team and the Centre for Capacity Research administration team, Annmarie Hand, Elly Wallis, Lorelei Silvester, Zena Parker who made the necessary logistical or administrative arrangements to allow a smooth data collection process. We would like to thank our Research Impact & Knowledge Translation Officer, Susie Crossman for her support in reviewing and editing the manuscript. We are grateful to Martina Savio, Dr Angela Obasi and Professor Stephen Bertel Squire from the IMPALA Management Team for their review of an earlier draft.

Funding

This research was funded by the National Institute for Health Research (NIHR) (project reference 16/136/35) using UK aid from the UK Government to support global health research. The views expressed in this publication are those of the author(s) and not necessarily those of the NIHR or the UK Department of Health and Social Care.

Competing interests

None declared.

Contributors

YD: Conceptualisation, Methodology, Data Curation; Writing: Original draft preparation. EMT: Methodology; Writing: Reviewing and Editing. IB: Conceptualisation, Methodology; Writing: Reviewing and Editing, Supervision. All authors read and approved the final manuscript.

Ethics approval

For peer review only

For peer review only - http://bmjopen.bmj.com/site/about/guidelines.xhtml

Ethical approval was provided by Liverpool School of Tropical Medicine's Research Ethics Committee (Reference: 18-031).

Data availability statement

The present study includes selected quotes to represent the content and themes across interviews. While requests for additional de-identified transcripts can be made to the authors, full interview transcripts cannot be made available as to do so would compromise anonymity and violate the terms of our ethical approval.

SUPPLEMENTARY FILES

Supplementary file 1: Survey information sheet

Supplementary file 2: Survey consent form

Supplementary file 3: Survey

Supplementary file 4: Interview guides

Supplementary file 5: Interview information sheet

Supplementary file 6: Interview consent form

Supplementary file 7: Observation form

References

1. Koplan, J.P., et al., *Towards a common definition of global health.* The Lancet, 2009. **373**(9679): p. 1993-1995.
2. Stipelman, B.A., et al., *Mapping the Impact of Transdisciplinary Research: A Visual Comparison of Investigator-Initiated and Team-Based Tobacco Use Research Publications.* Journal of Translational Medicine & Epidemiology, 2014. **2**(2).
3. Ding, Y., J. Pulford, and I. Bates, *Practical actions for fostering cross-disciplinary global health research: lessons from a narrative literature review.* BMJ Global Health, 2020. **5**(4): p. e002293.

For peer review only

4. Zscheischler, J. and S. Rogga, *Transdisciplinarity in land use science – A review of concepts, empirical findings and current practices.* Futures, 2015. **65**: p. 28-44.
5. Michael O'Rourke, Stephen Crowley, and C. Gonnerman, *On the nature of cross-disciplinary integration: A philosophical framework.* Studies in History and Philosophy of Science Part C: Studies in History and Philosophy of Biological and Biomedical Sciences, 2016. **56**: p. 62 - 70.
6. Sonnenwald, D.H., *Scientific collaboration.* Annual review of information science and technology, 2007. **41**(1): p. 643-681.
7. Shiffman, J., *Four Challenges That Global Health Networks Face.* International journal of health policy and management, 2017. **6**(4): p. 183-189.
8. Al Khaldi, M., et al., *Challenges and prospects in the public health research system in the occupied Palestinian territory: a qualitative study.* The Lancet, 2018. **391**: p. S25.
9. Hall, K.L., et al., *A four-phase model of transdisciplinary team-based research: goals, team processes, and strategies.* 2012.
10. LSTM. *NIHR Global Health Research Unit on Lung Health and Tuberculosis in Africa at LSTM.* 2020 27/09/2021 [cited 2021 23/03/2020]; Available from: https://www.lstmed.ac.uk/impala.
11. Stokols, D., *Toward a science of transdisciplinary action research.* Am J Community Psychol, 2006. **38**(1-2): p. 63-77.
12. Verschuren, P., H. Doorewaard, and M. Mellion, *Designing a research project*. Vol. 2. 2010: Eleven International Publishing The Hague.
13. Ritchie, J., et al., *Designing and selecting samples*, in *Qualitative research practice: A guide for social science students and researchers*, J. Ritchie, et al., Editors. 2013, sage.
14. Fuqua, J., et al., *Transdisciplinary collaboration as a basis for enhancing the science and prevention of substance use and "abuse".* Substance use & misuse, 2004. **39**(10-12): p. 1457-1514.
15. Riessman, C.K., *Narrative methods for the human sciences*. 2008: Sage.
16. Davé, A., et al., *Case Study Review of Interdisciplinary Research in Higher Education Institutions in England*. 2016, Technopolis.
17. Kneipp, S.M., et al., *Nurse scientists overcoming challenges to lead transdisciplinary research teams.* Nursing Outlook, 2014. **62**(5): p. 352-61.
18. Mesmer-Magnus, J.R. and L.A. DeChurch, *Information sharing and team performance: A meta-analysis.* Journal of applied psychology, 2009. **94**(2): p. 535.
19. Stephenson, J., et al., *The Practice of Interdisciplinarity.* International Journal of Interdisciplinary Social Sciences, 2010. **5**(7).
20. Zscheischler, J., S. Rogga, and M. Busse, *The Adoption and Implementation of Transdisciplinary Research in the Field of Land-Use Science—A Comparative Case Study.* Sustainability, 2017. **9**(11): p. 1926.
21. Stokols, D., et al., *Evaluating transdisciplinary science.* Nicotine & Tobacco Research, 2003. **5**(Suppl_1): p. S21-S39.
22. Gavens, L., et al., *Interdisciplinary working in public health research: a proposed good practice checklist.* Journal of Public Health, 2017. **40**(1): p. 175-182.
23. Jeffrey, P., *Smoothing the Waters: Observations on the Process of Cross-Disciplinary Research Collaboration.* Social Studies of Science, 2003. **33**(4): p. 539-562.
24. Karp, G.G., et al., *Facilitating collaborative interdisciplinary research: exploring process and implications for leisure scholars.* Leisure/Loisir, 2016. **40**(2): p. 201-223.
25. Kirk-Lawlor, N. and S. Allred, *Group Development and Integration in a Cross-Disciplinary and Intercultural Research Team.* Environmental Management, 2017. **59**(4): p. 665-683.
26. Sutherland Olsen, D., *Emerging interdisciplinary practice: making nanoreactors.* The Learning Organization, 2009. **16**(5): p. 398-408.

For peer review only - http://bmjopen.bmj.com/site/about/guidelines.xhtml

27. Vogel, A.L., et al., *Pioneering the Transdisciplinary Team Science Approach: Lessons Learned from National Cancer Institute Grantees.* Journal of translational medicine & epidemiology, 2014. **2**(2): p. 1027.
28. Research;, C.o.F.I., et al., *Facilitating Interdisciplinary Research* 2005, Washington, D.C.: The National Academies Press.
29. Bruce, A., et al., *Interdisciplinary integration in Europe: the case of the Fifth Framework programme.* Futures, 2004. **36**(4): p. 457-470.
30. Corner, J. and C. Normand, *Working together: crossing discipline boundaries to evaluate complex interventions.* NT Research, 2001. **6**(5): p. 829-836.
31. Powell, R.A., et al., *Transcending differences to study the transcendent: an exploratory study of researchers' and chaplains' reflections on interdisciplinary spiritual care research collaboration.* BMC Palliative Care, 2015. **14**: p. 12-12.
32. Dewulf, A., et al., *A Framing Approach to Cross-disciplinary Research Collaboration Experiences from a Large-scale Research Project on Adaptive Water Management.* Ecology and Society, 2007. **12**(2).
33. Rolland, B., et al., *Operationalization, Implementation, and Evaluation of Collaboration Planning: A Pilot Interventional Study of Nascent Translational Teams.* Journal of Clinical and Translational Science, 2020: p. 1-20.
34. Spiller, K., et al., *Carnivalesque collaborations: reflections on 'doing' multi-disciplinary research.* Qualitative Research, 2015. **15**(5): p. 551-567.
35. Trussell, D.E., et al., *Negotiating the Complexities and Risks of Interdisciplinary Qualitative Research.* International Journal of Qualitative Methods, 2017. **16**(1): p. 1609406917711351.
36. Podsakoff, P.M., MacKenzie, Scott B.,Lee, Jeong-Yeon,Podsakoff, Nathan P., *Common method biases in behavioral research A critical review of the literature and recommended remedies.* Journal of Applied Psychology, 2003. **88**(5): p. 5.

FIGURE TITLES

Figure 1: The IMPALA Organogram

Figure 2: The three-component framework for the cross disciplinary collaborative research process used in this study (adapted from Hall et al., 2012)

For peer review only

Figure 1: The IMPALA Organogram

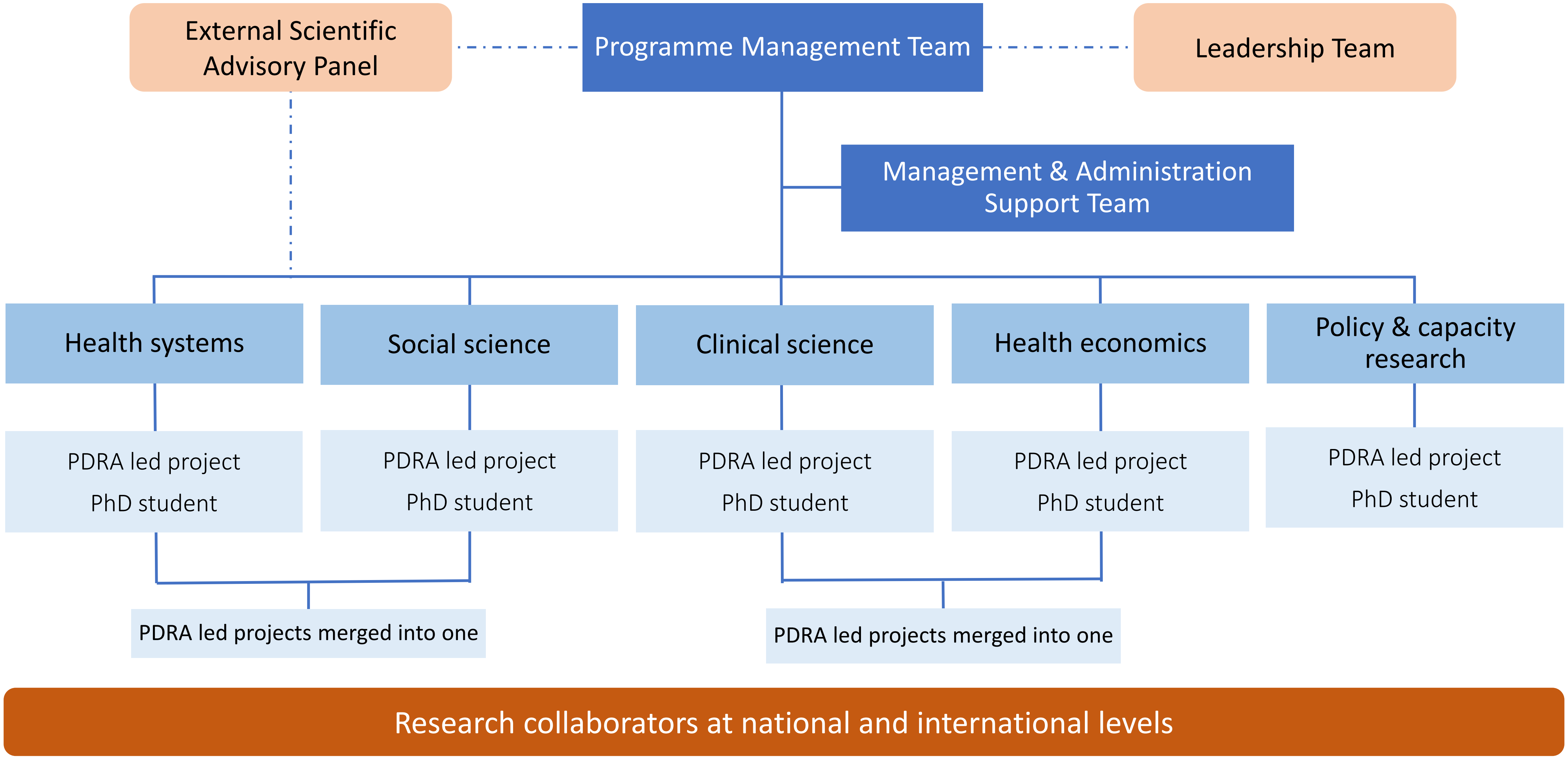

Figure 2: The three-component framework for the cross-disciplinary collaborative research process used in this study (adapted from Hall et al., 2012)

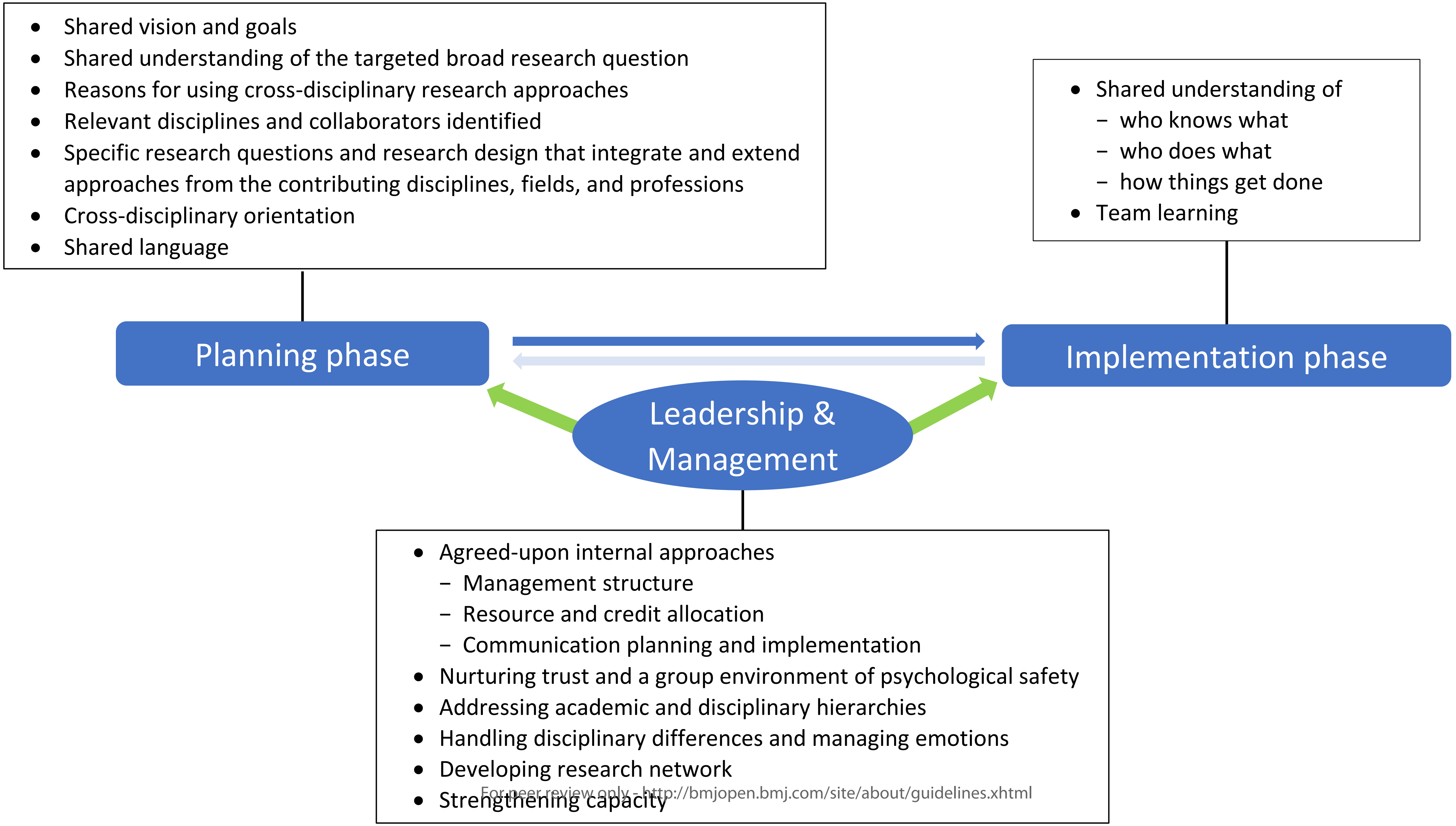

Version 1, 18 March 2018

Multidisciplinary cross-cutting capacity development project (MUDI)

Participant Information Leaflet

-For online survey-

My name is Yan Ding and I work for Capacity Research Unit, Liverpool School of Tropical Medicine. We would like to invite you for an online survey in our research, the MUDI project. Before you decide, we would like you to understand why the research is being done and what it would involve for you Please take time to read the following information carefully.

What is the purpose of the online survey?

The purpose of the survey is to map competencies and experience in conducting multidisciplinary research.

What is the MUDI project?

MUDI is a multidisciplinary capacity development research project under the umbrella of IMPALA which stands for the International Multidisciplinary Programme to Address Lung Health and TB in Africa. IMPALA is a four-year collaborative programme funded by the National Institute for Health Research in the UK.

Why have you been invited?

You have at least one of the following roles in IMPALA:
1) a member of the External Scientific Advisory Panel;
2) a member of the IMPALA leadership team;
3) a member of IMPALA project teams, either from LSTM, or from any collaborating organizations but not for administration and finance;
4) a member of an underpinning collaborations and partnerships of IMPALA

What are the possible benefits of the survey?

This survey will yield valuable information on competencies and experience in conducting multidisciplinary research for MUDI to study the facilitators and barriers of multidisciplinary research at individual level.

Do I have to take part?

It is entirely voluntary. It is up to you to decide whether to take part or not. If you choose to take part, you are free to withdraw from the research study at any time, without giving a reason. If you choose not to participate this will not affect your work or career in any way. You do not have to answer any questions with which you do not feel comfortable.

What will I have to do?

This survey has four components: 1) personal information; 2) education background; 3) previous experience in multidisciplinary research; and 4) self-assessment of capacities in multidisciplinary research. The survey will take about 15 minutes to complete.

Confidentiality

Identifying information such as your name and organization will be asked, as we would like to come back to some of you for semi-structured interviews and ongoing data collection during the implementation of IMPALA. Information on expertise, competencies, gender, age and geographical location provided by this survey will be used to maximize diversity of the interviewees which makes

For peer review only

non-anonymization of the survey necessary. All the answers you provide in this survey will be kept confidential and only shared among the Capacity Research Unit researchers directly involved in the MUDI project. The survey data will be reported in a summary fashion only and will not identify any individual person.

Will I be reimbursed for my time?
You will not be paid for being part of this project.

Will you participate in this study? Yes or No
If yes, please go to the next page and indicate your consent in the tick box provided.

For further details, or if you have any questions or want to file a complaint about the research you may contact:

Organisation responsible for the study:

Dr. Yan Ding

Liverpool School of Tropical Medicine, UK.

E-mail: yan.ding@lstmed.ac.uk.

The LSTM Research Ethics Committee

E-mail: lstmrec@lstmed.ac.uk

For peer review only

Version 1, 18 March 2018

ELECTRONIC CONSENT FORM*
-online survey-

**Study Title: Competencies and experience in multidisciplinary research**
**Principal Investigator: Prof. Imelda Bates, Liverpool School of Tropical Medicine, UK.**

☐ I have read the information sheet concerning this study and I understand what will be required of me if I take part in this study. I understand that at any time, I may withdraw from this study without giving a reason and without affecting my participation in any research activities or consortium I am involved in. I agree to take part in this study.

***To be inserted as the third page of the online survey (following information sheet).**

For peer review only

Confidence and experience in multidisciplinary research

Page 1: Welcome!

IMPALA MUDI beginning-of-project survey:

Experience and Confidence in Multidisciplinary Research

Version 7, 21 May 2018

What is your role in IMPALA? (screening question) ✱ *Required*

- a member of the External Scientific Advisory Panel
- a member of the IMPALA leadership team
- a member of IMPALA project teams or a collaborator but not purely involved in administration and finance
- an administration or finance staff member
- I am not involved in IMPALA
- Other

If you selected Other, please specify:

For peer review only - http://bmjopen.bmj.com/site/about/guidelines.xhtml

Page 2: Participant Information Leaflet

Version 2, 18 May 2018

Multidisciplinary cross-cutting capacity development project (MUDI)

Participant Information Leaflet

-Beginning-of-project survey-

My name is Yan Ding and I work for the Capacity Research Unit, Liverpool School of Tropical Medicine. We would like to invite you to participate in an online survey in our research, the MUDI project. Before you decide, we would like you to understand why the research is being done and what it would involve for you Please take time to read the following information carefully.

What is the purpose of the online survey?

The purpose of the survey is to map experience and confidence in conducting multidisciplinary research.

What is the MUDI project?

MUDI is a multidisciplinary capacity development research project under the umbrella of IMPALA (International Multidisciplinary Programme to Address Lung Health and TB in Africa). IMPALA is a four-year collaborative programme funded by the National Institute for Health Research in the UK.

Why have you been invited?

You have at least one of the following roles in IMPALA:

1) a member of the External Scientific Advisory Panel;

For peer review only - http://bmjopen.bmj.com/site/about/guidelines.xhtml

2) a member of the IMPALA leadership team;

3) a member of IMPALA project teams or a collaborator.

What are the possible benefits of the survey?

This survey will yield valuable information on experience and confidence in conducting multidisciplinary research for MUDI to study the facilitators and barriers of multidisciplinary research at individual level.

Do I have to take part?

It is entirely voluntary. It is up to you to decide whether to take part or not. If you choose to take part, you are free to withdraw from the research study at any time, without giving a reason. If you choose not to participate this will not affect your work or career in any way. You do not have to answer any questions with which you do not feel comfortable.

What will I have to do?

This survey has four components: 1) personal information; 2) education background; 3) previous experience in multidisciplinary research; and 4) confidence in multidisciplinary research. The survey will take about 15 to 20 minutes to complete.

Confidentiality

Identifying information such as your name and organization will be asked, as we would like to come back to some of you for semi-structured interviews and ongoing data collection during the implementation of IMPALA. Information on experience, confidence, gender, age and geographical location provided by this survey will be used to maximize diversity of the interviewees which makes non-anonymization of the survey necessary. All the answers you provide in this survey will be kept confidential and only shared among the Capacity Research Unit researchers directly involved in the MUDI project. The survey data will be reported in a summary fashion only and will not identify any individual person. The anonymised results of the survey will be shared through IMPALA.

For peer review only - http://bmjopen.bmj.com/site/about/guidelines.xhtml

Will I be reimbursed for my time?

You will not be paid for being part of this project.

Will you participate in this study? Yes or No

If yes, please go to the next page and indicate your consent in the tick box provided.

For further details, or if you have any questions or want to file a complaint about the research you may contact:

Organisation responsible for the study:

Dr. Yan Ding

Liverpool School of Tropical Medicine, UK.

E-mail: yan.ding@lstmed.ac.uk.

The LSTM Research Ethics Committee:

E-mail: lstmrec@lstmed.ac.uk

Do you agree to take this online survey? ✱ *Required*

- Yes
- No

For peer review only - http://bmjopen.bmj.com/site/about/guidelines.xhtml

Page 3: Electronic Consent Form

Study Title: Competencies and experience in multidisciplinary research

Principal Investigator: Prof. Imelda Bates, Liverpool School of Tropical Medicine, UK.

Thank you for agreeing to take this survey. Please tick the statement to continue.

- [ ] I have read the information sheet concerning this study and I understand what will be required of me if I take part in this study. I understand that at any time, I may withdraw from this study without giving a reason and without affecting my participation in any research activities or consortium I am involved in. I agree to take part in this study.

For peer review only

For peer review only - http://bmjopen.bmj.com/site/about/guidelines.xhtml

Page 4: Main body of the survey

Section 1: Personal information

First Name, Last Name ✱ *Required*

Your gender: ✱ *Required*

- Female
- Male
- Prefer not to say

Which year were you born? ✱ *Required*

Your current country of residence: ✱ *Required*

Organization where you work? ✱ *Required*

For peer review only - http://bmjopen.bmj.com/site/about/guidelines.xhtml

Job title at your organization ✱ *Required*

Your main responsibility in IMPALA ✱ *Required*

Section 2: Education background

What degrees have you completed? Please choose multiple answers if you have more than one answer ✱ *Required*

- ☐ Bachelor's degree
- ☐ Master's degree
- ☐ Doctorate degree
- ☐ Other

If you selected Other, please specify:

For peer review only - http://bmjopen.bmj.com/site/about/guidelines.xhtml

Please write down the discipline (s) of **all** degrees you have completed and separate them by comma. **Discipline** refers to a particular branch of learning or body of knowledge such as history, sociology, economics. ✱ *Required*

Which of the following option do you think applies to your professional background? ✱ *Required*

- Unidisciplinary
- Multidisciplinary

Section 3: Experience in multidisciplinary research

Multidisciplinary research in this study is research that uses knowledge, study design and methodology from multiple disciplines regardless of the extent of integration of disciplinary-specific theories, concepts, and approaches to address common problems.

Did you have **previous experience** of the following activities before participated in the IMPALA programme? (check the one that best applies)

Creating a network for further collaborations ✱ *Required*

- Yes
- No
- I don't remember

For peer review only - http://bmjopen.bmj.com/site/about/guidelines.xhtml

If yes, please write down one example:

Extending a network for further collaborations ✱ *Required*

- ◌ Yes
- ◌ No
- ◌ I don't remember

If yes, please write down one example:

Reading academic articles **outside** your discipline? ✱ *Required*

- ◌ Yes
- ◌ No
- ◌ I don't remember

For peer review only - http://bmjopen.bmj.com/site/about/guidelines.xhtml

If yes, please specify the disciplines:

Have you drafted funding proposals for multidisciplinary research projects /programs in partnership with scholars from other disciplines? ✱ *Required*

- Yes
- No
- I don't remember

Proactively seeking an exchange of theories, concepts, and approaches with those from other disciplines? ✱ *Required*

- Yes
- No
- I don't remember

If yes, please specify the disciplines:

Have you modified your own perspectives on research questions or research plan as a result of interactions with colleagues from fields other than your own? ✱ *Required*

For peer review only - http://bmjopen.bmj.com/site/about/guidelines.xhtml

- Yes
- No
- I don't remember

If yes, please write down one example:

Have you coordinated fieldwork with team members in a multidisciplinary research to maximize the leverage? ✱ *Required*

- Yes
- No
- I don't remember

If yes, please write down one example:

Have you combined research outcomes of contributing disciplines in a multidisciplinary

For peer review only - http://bmjopen.bmj.com/site/about/guidelines.xhtml

research project into a final technical output/publication? ✱ *Required*

- Yes
- No
- I don't remember

If yes, please write down one example:

Have you disseminated multidisciplinary research results to **policy makers**? ✱ *Required*

- Yes
- No
- I don't remember

If yes, please write down one example:

For peer review only - http://bmjopen.bmj.com/site/about/guidelines.xhtml

Have you advocated for multidisciplinary research? ✱ *Required*

- Yes
- No
- I don't remember

If yes, please write down one example:

Section 4: Confidence in multidisciplinary research

Please choose one response for each question that best describes your confidence.

5 means extremely confident and 1 extremely unconfident

Do you feel confident to do the following activities?

To create a network for further collaborations ✱ *Required*

- 5. Extremely confident
- 4.
- 3.
- 2.
- 1. Extremely unconfident

For peer review only - http://bmjopen.bmj.com/site/about/guidelines.xhtml

To extend a network for further collaborations ✱ Required

- 5. Extremely confident
- 4.
- 3.
- 2.
- 1. Extremely unconfident

To draft funding proposals for multidisciplinary research projects /programs in partnership with scholars from other disciplines ✱ Required

- 5. Extremely confident
- 4.
- 3.
- 2.
- 1. Extremely unconfident

To establish trust among research team members ✱ Required

- 5. Extremely confident
- 4.
- 3.
- 2.
- 1. Extremely unconfident

To articulate your research questions, theories, concepts and approaches orally to those from other disciplines? ✱ Required

For peer review only - http://bmjopen.bmj.com/site/about/guidelines.xhtml

- 5. Extremely confident
- 4.
- 3.
- 2.
- 1. Extremely unconfident

To engage colleagues from other disciplines to gain their perspectives on **research questions and research plan** ✱ *Required*

- 5. Extremely confident
- 4.
- 3.
- 2.
- 1. Extremely unconfident

To modify your own perspectives on **research questions and plan** as a result of interactions with those from other disciplines ✱ *Required*

- 5. Extremely confident
- 4.
- 3.
- 2.
- 1. Extremely unconfident

To learn new knowledge and extend the width of your knowledge beyond your primary discipline ✱ *Required*

For peer review only - http://bmjopen.bmj.com/site/about/guidelines.xhtml

- 5. Extremely confident
- 4.
- 3.
- 2.
- 1. Extremely unconfident

To actively participate in group discussions/meetings in a multidisciplinary research ✱ *Required*

- 5. Extremely confident
- 4.
- 3.
- 2.
- 1. Extremely unconfident

To coordinate fieldwork with team members to maximize the leverage ✱ *Required*

- 5. Extremely confident
- 4.
- 3.
- 2.
- 1. Extremely unconfident

To interact with non-academic stakeholders in general in a multidisciplinary research ✱ *Required*

- 5. Extremely confident

For peer review only - http://bmjopen.bmj.com/site/about/guidelines.xhtml

- 4.
- 3.
- 2.
- 1. Extremely unconfident

To combine research outcomes of contributing disciplines into a final technical output ✱ *Required*

- 5. Extremely confident
- 4.
- 3.
- 2.
- 1. Extremely unconfident

To share research from your discipline in language understandable by those outside your discipline ✱ *Required*

- 5. Extremely confident
- 4.
- 3.
- 2.
- 1. Extremely unconfident

To disseminate multidisciplinary research results to policy makers ✱ *Required*

- 5. Extremely confident
- 4.

For peer review only - http://bmjopen.bmj.com/site/about/guidelines.xhtml

- 3.
- 2.
- 1. Extremely unconfident

To advocate for multidisciplinary research ✱ *Required*

- 5. Extremely confident
- 4.
- 3.
- 2.
- 1. Extremely unconfident

For peer review only - http://bmjopen.bmj.com/site/about/guidelines.xhtml

Page 5: Thank you for completing the survey

We really appreciate your input and time.

If you have any question, please feel free to contact Yan at yan.ding@lstmed.ac.uk.

Wish you all the best.

For peer review only

For peer review only - http://bmjopen.bmj.com/site/about/guidelines.xhtml

Version 4, 20 March 2018

IMPALA

Multidisciplinary cross-cutting capacity development project (MUDI)

Topic guides for semi-structured interviews, baseline survey

IMPALA team structure

Contents

1 Background information to assist the identification of MUDI interviewees 1

2 A topic guide for interviews with a member from the external scientific advisory board 3

3 A topic guide for interviews with the directors of IMPALA 4

4 A topic guide for interviews with researchers of the two applied projects, including from LSTM (except IMPALA Directors), from project country teams in Sudan, Tanzania and Uganda, and from institutions in other African countries 7

1 Background information to assist the identification of MUDI interviewees

MUDI takes the two phase-1 applied research projects within IMPALA as case studies. We would like to interview a member of the external scientific advisory board, the IMPALA directors, and researchers working on the two projects, including these based in LSTM, from project country teams and from institutions in other countries in Africa. Figure 1 presents the team structure of the two phase-1 applied research projects within IMPALA to assist the identification of interviewees.

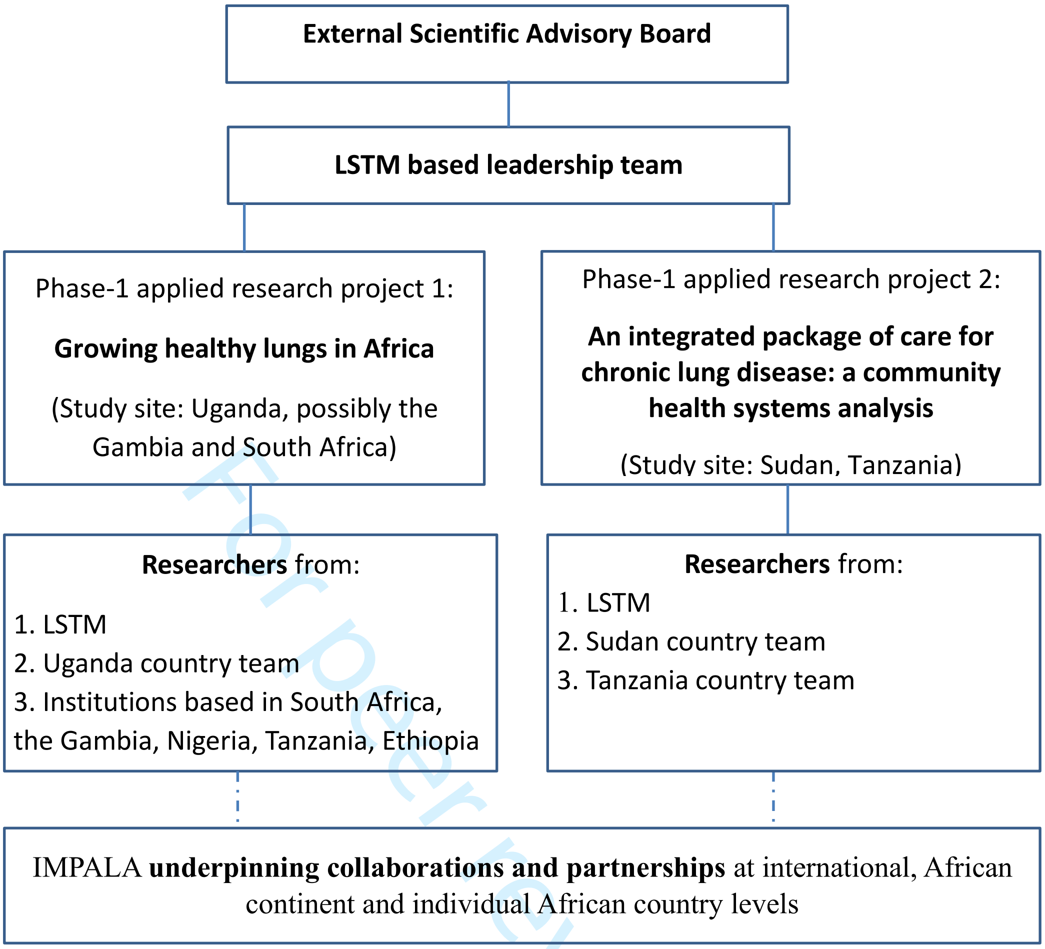

Figure 1: The team structure of the two phase-1 applied research projects within IMPALA

For peer review only

2 A topic guide for interviews with a member from the external scientific advisory board

1) **Introduction**
   - to be explained to interviewees
   - will first go through the information sheet with the interviewees
   - ask for consent to have the interviews & permission for audio-recording

2) **Background**
   - Academic qualification (bachelor degree? masters' degree? doctorate? Other specialist training?)
   - What's your role within your organization?
   - Personal research and work experience in multi-, inter- and trans-disciplinary collaboration
   - Could you please tell me about your role as a member of the external scientific advisory board member? Your main responsibilities and your participation so far.
3) **Examples of research involving multiple disciplines of where it worked well and not well in your experience**.
4) **IMPALA**
   - What's your vision for IMPALA?
   - What are the collaborations among disciplines do you perceive in IMPALA?
   - How do you define multidisciplinary research?
   - In MUDI, we define **multidisciplinary research** in this study is research that uses knowledge, study design and methodology from multiple disciplines, and which is based on a shared conceptual framework drawing together disciplinary-specific theories, concepts, and approaches to address common problems.

     What kind of multidisciplinary research do you think the two phase-1 projects respectively are at their current stages in the abovementioned three kinds of situation? Is there any observed movement of the two projects among the three kinds of situation for multidisciplinary research according to your observation so far?
   - According to your experience and knowledge, what do you think would be the facilitator and barriers of a multi-disciplinary collaboration?
5) **Strategy in influencing practice and policy**
   - Would you put applying knowledge from research to **practice** as a vision, or an aim?
   - Would you put applying knowledge from research to **policy** as a vision, or an aim?
   - Do you think it is possible to demonstrate IMPALA's impact on practice and policy?
   - What would be your suggestions?
6) Any other things you would like to add?

For peer review only

3 A topic guide for interviews with the directors of IMPALA

1) **Introduction**
2) **Personal background**
    - Academic qualification (bachelor degree? masters' degree? doctorate? other specialist training?)
    - Personal research and work experience in multi-, inter- and trans-disciplinary collaboration
3) **IMPALA**
    - What disciplines are involved in IMPALA?
    - How did you identify these disciplines for a collaboration?
    - Why such a multi-disciplinary collaboration?
    - What do you want to achieve through such a collaboration?
4) **Facilitating multidisciplinary research**
    - How do you facilitate multidisciplinary research?
      Probes:
      What strategy?
      What techniques?
5) **Influencing practice and policy**
    - Would you put applying knowledge from research to **practice as a vision, or an aim for IMPALA**?
    - Would you put applying knowledge from research to **policy as a vision, or an aim for IMPALA**?
6) **Your participation in the phase-1 project (s)**
    - <u>Research questions and decide to work together</u>
      -What specific research questions are you addressing to achieve the project aim?
      -Do you need other disciplines to answer the research questions? If so, which ones? Why?

      -Have you modified the research questions for this project as a result of interactions with colleagues from fields other than your own? If yes, could you tell me more?
      -Do you have any pre-experience in modifying your own research questions as a result of interactions with colleagues from fields other than your own? If yes, could you please give me examples?

    - <u>Bring in knowledge, study design and methodology from multiple disciplines</u>

      -Have you contributed to the study design of the project? If so, could you please tell me more about your input?
      -Have you contributed to the methodology of the project? If so, could you please tell me more about your input?
      -Who else also contributed to the study design?
      -Who else also contributed to the methodology?
      -In this project, have you modified your study design as a result of interactions with colleagues from fields other than your own? If so, could you tell me more?

For peer review only

-In this project, have you modified your own research methods as a result of interactions with colleagues from fields other than your own? If so, could you tell me more?
-How do you feel about the knowledge, study design and methodology brought in from other disciplines?

- -How do you define multidisciplinary research?

- Researchers from different disciplines work together in one project to address a common problem. There are different ways of working together.
  For example: researchers with different disciplinary background address different aspects of a problem based on their disciplinary background. They work independently from researchers of other disciplinary background. As each discipline addresses one aspect of the problem, together, the problem has been addressed in a more comprehensive way.
  Another example: researchers are still in charge of studying different aspects of a problem based on their disciplinary background. Besides this, they have interactions and influence each other's study design and project activities.
  What's the way that researchers from this project work together?

7) Involvement and interaction with non-academic stakeholders of the project

- Is there any non-academic stakeholder that involve in the project so far?
- If yes:
  -Who are they?
  -Do you know their disciplinary background?
  -What project activities have they participate in? Since when?
  -Any perspectives from them have been integrated in the project? If so, what?
- Collaboration history
  -Among all researchers or called project team members in this phase-1 applied research project, is there any collaboration between you and any of your project team members before this project? (Probe: from project members from other organizations, project member with different disciplinary background and from the same organization)
  If there are some, could you give me examples, including research topics and how long you worked together.

8) Facilitators and barriers

- From all your previous experience, what things have you found make it easier for you to work with colleagues from other disciplines, and why? (probe: institutional level, program level, project level, individual level)
- Also from all your previous experience, what things have you found make it difficult for you to work with colleagues from other disciplines, and why? (probe: institutional level, program level, project level, individual level)
- Have you encountered any barriers related to collaboration among multiple disciplines when you participated in designing the project in the intervention countries? If yes, what are they? How do you cope with them so far?

For peer review only

9) Expectations

- What's your expectations for IMPALA?
- Expectations for the project (s)?
- What would you expect for yourself to achieve in participating in this multidisciplinary research?
  (probe: experiences? Competencies?)
- Do you plan to disseminate your research findings in this phase-1 project to researchers in other disciplines? Why?
- How do you think collaboration with other disciplines' influence on your research career in terms of academic performance? (at your institution, in your country and at global level)

10) Next steps

- What kind of competencies in multidisciplinary research do you feel like to have further improvement
  Probes: 1) personal knowledge and skills in investigating a research question by integrating theories and methods of other disciplines into your primary discipline; 2) communicating with researchers from other disciplines, for example describing your research perspective to them, disseminating your research among them; and 3) interacting with researchers from other disciplines, such as writing a joint proposal, attending scholarly presentations in other disciplines.
- How will you work together with other researchers in the coming year for the phase-1 project? (divisions of responsibilities and joint efforts? Communication plan? Interaction?)
- with non-academic stakeholders?

11) Any other things you would like to add?

For peer review only

4 A topic guide for interviews with researchers of the two applied projects, including from LSTM (except IMPALA Directors), from project country teams in Sudan, Tanzania and Uganda, and from institutions in other African countries

1) **Introduction**
   - To be explained to interviewees
   - Will first go through the information sheet with the interviewees
   - Ask for consent to have the interviews & permission for audio-recording
2) **Background**
   - Academic qualification (bachelor degree? masters' degree? doctorate? other specialist training?)
   - What's your role within your organization? Within IMPALA? Within the two phase-1 projects
   - Personal research and work experience in multi-, inter- and trans-disciplinary collaboration
3) **IMPALA, phase-1 project (s) (discipline related)**
   - Research questions and decide to work together

     -What specific research questions are you addressing to achieve the project aim?
     -Do you need other disciplines to answer the research questions? If so, which ones? Why?
     -Have you modified the research questions for this project as a result of interactions with colleagues from fields other than your own? If yes, could you tell me more?
     -Do you have any pre-experience in modifying your own research questions as a result of interactions with colleagues from fields other than your own? If yes, could you please give me examples?

   - Bring in knowledge, study design and methodology from multiple disciplines

     -Have you contributed to the study design of the project? If so, could you please tell me more about your input?
     -Have you contributed to the methodology of the project? If so, could you please tell me more about your input?
     -Who else also contributed to the study design?
     -Who else also contributed to the methodology?
     -In this project, have you modified your study design as a result of interactions with colleagues from fields other than your own? If so, could you tell me more?
     -In this project, have you modified your own research methods as a result of interactions with colleagues from fields other than your own? If so, could you tell me more?
     -How do you feel about the knowledge, study design and methodology brought in from other disciplines?

   - How do you define multidisciplinary research?

For peer review only

- Researchers from different disciplines work together in one project to address a common problem. There are different ways of working together.
  For example: researchers with different disciplinary background address different aspects of a problem based on their disciplinary background. They work independently from researchers of other disciplinary background. As each discipline addresses one aspect of the problem, together, the problem has been addressed in a more comprehensive way.
  Another example: researchers are still in charge of studying different aspects of a problem based on their disciplinary background. Besides this, they have interactions and influence each other's study design and project activities.
  What's the way that researchers from this project work together?

  c
  -?

4) Involvement and interaction with non-academic stakeholders of the project

- Is there any non-academic stakeholder that involve in the project so far?
- If yes:
  -Who are they?
  -Do you know their disciplinary background?
  -What project activities have they participate in? Since when?
  -Any perspectives from them have been integrated in the project? If so, what?
- Collaboration history
  -Among all researchers or called project team members in this phase-1 applied research project, is there any collaboration between you and any of your project team members before this project? (Probe: project members from other organizations, project member with different disciplinary background and from the same organization)
  If there are some, could you give me examples, including research topics and how long you worked together.

5) Facilitators and barriers

- From all your previous experience, what things have you found make it easier for you to work with colleagues from other disciplines, and why? (probe: institutional level, program level, project level, individual level)
- Also from all your previous experience, what things have you found make it difficult for you to work with colleagues from other disciplines, and why? (probe: institutional level, program level, project level, individual level)
- Have you encountered any barriers related to collaboration among multiple disciplines when you participated in designing the project in the intervention countries? If yes, what are they? How do you cope with them so far?

6) Expectations

- What's your expectations for IMPALA?
- Expectations for the project (s)?
- What would you expect for yourself in participating in the multidisciplinary research project?
  (probe: experience? competencies?)
- Do you plan to disseminate your research findings in this phase-1 project to researchers in other disciplines? Why?

For peer review only

- How do you think collaboration with other disciplines' influence on your research career in terms of academic performance? (at your institution, in your country and at global level)

7) Next steps

- What kind of competencies in multidisciplinary research do you feel like to have further improvement
  Probes:
  1) **personal knowledge and skills** in investigating a research question by integrating theories and methods of other disciplines into your primary discipline;
  2) **communicating** with researchers from other disciplines, for example describing your research perspective to them, disseminating your research among them; and
  3) **interacting** with researchers from other disciplines, such as writing a joint proposal, attending scholarly presentations in other disciplines.
- How will you work together with other researchers in the coming year for the phase-1 project? (divisions of responsibilities and joint efforts? Communication plan? Interaction?)
- with non-academic stakeholders?

8) Any other things you would like to add?

For peer review only

Version 2, 18 May 2018

Multidisciplinary cross-cutting capacity development project (MUDI)

Participant Information Leaflet

-For interviews-

My name is Yan Ding and I work for Capacity Research Unit, Liverpool School of Tropical Medicine. We would like to invite you for an interview in our research, the MUDI project. Before you decide, we would like you to understand why the research is being done and what it would involve for you. ***I will go through the information sheet with you and answer any questions you have.*** *This should take about 5 minutes. Ask me if there is anything that is not clear.*

What's the purpose of the interviews?

The MUDI project aims to generate robust evidence about what works for fostering research that involves multiple disciplines, and in what contexts, taking lung health and TB as a pathfinder. Also, it aims to develop evidence-informed and transferable recommendations for actions (and possibly a benchmark) that can catalyze effective and sustainable collaborative research of multiple disciplines in low and middle-income countries.

We conduct interviews besides other methods such as a survey to generate data to achieve the MUDI project's aims.

What is the MUDI project?

MUDI is a multidisciplinary capacity development research project under the umbrella of IMPALA which stands for the International Multidisciplinary Programme to Address Lung Health and TB in Africa. IMPALA is a four-year collaborative programme funded by the National Institute for Health Research in the UK.

Why have you been invited?

MUDI takes two multidisciplinary applied research projects in IMPALA as case studies, and they are: 1) Clinical and Socioeconomic determinants of lung function among young infants in Uganda: a birth cohort study; and 2) an integrated health systems approach for improving health services for chronic lung disease in Sudan and Tanzania.

You have at least one of the following roles: 1) a member of the External Scientific Advisory Board of IMPALA; 2) a director or co-director of IMPALA; 3) a researcher of at least one of the two abovementioned multidisciplinary applied research projects in IMPALA; and 4) a member of the project administrative team. Your experience and opinion in fostering research that involve multiple disciplines would be helpful for MUDI to identify barriers and facilitators of research with multiple disciplines, and alto to develop evidence informed recommendations in promoting effective and sustainable multidisciplinary research.

Do you have to take part?

It is up to you to decide whether to join the study. We will describe the study and go through this information sheet. If you agree to take part, we will then ask you to sign a consent form. You are free to withdraw at any time, without giving a reason. This would not affect the standard of care you receive.

What will happen to you if you take part?

Each interview will last between half an hour to one hour, and altogether you may be invited for such an interview with a maximum of 4 times in the following 3 years.

For peer review only - http://bmjopen.bmj.com/site/about/guidelines.xhtml

Questions during the interview are related to your previous experience in multi-/inter-disciplinary research, changes and interactions of collaboration among different disciplines in IMPALA and its two-abovementioned multidisciplinary projects or regarding to barriers and facilitators of multidisciplinary research. You may feel discomfort to answer certain questions and you can choose not to answer at any time.

Compensation

You will not be paid for being part of this project.

What are the possible benefits of taking part?

The interview will yield valuable information on the interaction of multiple disciplines in IMPALA, on facilitators and barriers of the two above mentioned multidisciplinary research in IMPALA. It makes it possible to share emerging lessons across IMPALA so that improvements to the programme can be made thus enhancing the effectiveness and value for money of the IMPALA programme, also beyond IMPALA.

What will happen to any data you give and to the results of the research study?

All information you provide through the interview will be kept confidential and only shared among Capacity Research Unit researchers directly involved in MUDI project for research purpose. The findings will be reported without identifiable individual information.

What will happen if you don't want to carry on with the study?

You can withdraw from the study at any time, and you do not need provide a reason. If you withdraw from the study, nothing will happen to you.

Will you participate in the interview? Yes or No

If yes, I will provide you a consent form and please sign on it.

If you decide to participant in the interview, may I do an audio-recording of the interview? Yes or No

We would like to have the interview audio-recorded to check all points are correctly captured in the interview. we will do so only with your permission.

Contact Details

For further details, or if you have any questions or want to file a complaint about the research you may contact:

Organisation responsible for the study:

Dr. Yan Ding

Liverpool School of Tropical Medicine, UK.

E-mail: yan.ding@lstmed.ac.uk.

The LSTM Research Ethics Committee

E-mail: lstmrec@lstmed.ac.uk

For peer review only - http://bmjopen.bmj.com/site/about/guidelines.xhtml

Consent Form

CONFIDENTIAL

Study Title: Multidisciplinary cross-cutting capacity development project (MUDI)	
**Principal Investigator: Professor Imelda Bates**	**Study Site: Sudan, Tanzania, Uganda. (for IMPALA's phase 1 non-embedded projects)**

	**Please initial box**
1. I confirm I have read and understood the information sheet dated 18 March 2018 (Version 1) for the above study. I have had the opportunity to consider the information, ask questions and have had these answered satisfactorily.	
2. I understand that participation in this study is voluntary and I am free to withdraw consent at any time, without giving a reason, without any penalties.	
3. I understand that data collected during the study, will be kept confidential and only shared among the Centre for Capacity Research researchers directly involved in the MUDI project for research purposes. The findings will be reported without identifiable individual information.	
4. I hereby declare that I have not been subjected to any form of coercion in giving this consent.	
5. I agree to take part in this study.	

Signing this declaration does not affect your right to decline to take part in any future study.

______________________	________	______________________
Name of participant	Date	Signature
______________________	________	______________________
Name of person taking Consent	Date	Signature

When complete: 1 copy for participant; 1 copy (original) for research

IMPALA MUDI Meeting Observation Form

Date	
Location	
Title/topic(s)	
Presenter(s)	
Attendees (PIs and core members)	
Attendees (Dept./School representatives)	
Attendees (from wider collaborative organizations)	
Attendees (Others)	
Total Attendance	
Observer	
Overall Tone	
Overall Energy	
To what extend did synergy occur at this meeting?	A. Not at all (=1) B. 2 C. 3 D. 4 E. Very much (=5) F. Not sure

**Focus**	**Transdisciplinary Components**				**Tone Scale**	**Energy Scale**
1 Administrative<br>2 Indiv./group presentation<br>3 Scientific discussion<br>4 Open discussion<br>5 Post Meeting/Side Bar<br>6 Breaks<br>7 Other	A= Cross-disciplinary synergy<br>Such as:<br>• Brainstorming crossing analytical levels<br>• Integration of disciplinary ideas	B=Proposed or actual cross-disciplinary outcomes<br>Such as:<br>• Progress towards cross-disciplinary model<br>• Discussions regarding new course<br>• Intention stated to meet further	C=Info/Support<br>Such as:<br>• Infor/data presented (not cross-dis)<br>• Material/Technical support (actual or offered)<br>• Socio-emotional support<br>• Humour/Mood enhancement<br>• Movement towards consensus	D=Setbacks<br>Such as:<br>• Critical statements<br>• Interrupting others<br>• Distracting events (equip trouble, unexpected noise)<br>• Movement towards dissensus	+2=Very harmonious<br>+1=Harmonious<br>0=Neutral<br>-1=Conflicted<br>-2=Very conflicted	5=Very high<br>4=High<br>3=Medium<br>2=Low<br>1=Very low

For peer review only

Time (start, end and sum min)	Focus (1-7)	Qualitative Description and Details	Component (A-D)	Tone (-2 to +2)	Energy (1 to 5)

Time (start, end and sum min)	Focus (1-7)	Qualitative Description and Details	Component (A-D)	Tone (-2 to +2)	Energy (1 to 5)

Note: This observation form took reference from: Fuqua, J., et al., Transdisciplinary collaboration as a basis for enhancing the science and prevention of substance use and "abuse". Substance use & misuse, 2004. 39(10-12): p. 1457-1514

For peer review only

For peer review only

For peer review only - http://bmjopen.bmj.com/site/about/guidelines.xhtml

Manuscript title:

Identifying actions to foster cross-disciplinary global health research: a mixed-methods qualitative case study of the IMPALA programme on lung health and tuberculosis in Africa

Research checklist: Standards for Reporting Qualitative Research (SRQR)[a]

No.	Topic	Item	Page no.	Notes
	**Title and abstract**			
S1	Title	Concise description of the nature and topic of the study Identifying the study as qualitative or indicating the approach (e.g., ethnography, grounded theory) or data collection methods (e.g., interview, focus group) is recommended	page (p) 1	We changed the title according to ed's suggestion. Thank you.
S2	Abstract	Summary of key elements of the study using the abstract format of the intended publication; typically includes background, purpose, methods, results, and conclusions	P2	The abstract follows a structure suggested by BMJ Open
	**Introduction**			
S3	Problem formulation	Description and significance of the problem/phenomenon studied; review of relevant theory and empirical work; problem statement	P3,4	Details in Introduction: Paragraph 2
S4	Purpose or research question	Purpose of the study and specific objectives or questions	P4	Details in the last paragraph of the Introduction
	**Methods**			
S5	Qualitative approach and research paradigm	Qualitative approach (e.g., ethnography, grounded theory, case study, phenomenology, narrative research) and guiding theory if appropriate; identifying the research paradigm (e.g., postpositivist, constructivist/ interpretivist) is also recommended; rationale[b]	P5-7	We adapted a framework to frame the study including data collection and analysis
S6	Researcher characteristics and reflexivity	Researchers' characteristics that may influence the research, including personal attributes, qualifications/experience, relationship with participants, assumptions, and/or presuppositions; potential or actual interaction between researchers' characteristics and the research questions, approach, methods, results, and/or transferability	P3	In "Strengths and limitations of this study" bulletin point 5, we reflected on our influence on interviewees.

For peer review only

S7	Context	Setting/site and salient contextual factors; rationale[b]	P4	We introduced the context and our case 6study programme in INTRODUCTION instead of METHODS as the context and case study helps understand our research question.
S8	Sampling strategy	How and why research participants, documents, or events were selected; criteria for deciding when no further sampling was necessary (e.g., sampling saturation); rationale[b]	P5,6	For the baseline survey, sampling was described in page 5 under "Baseline survey"; For Semi-structured interviews, relevant info was also in page 5 under "Sample selection"; For Document review, all relevant documents were included as described in page 6 under "Document review"; For "Observation of events", all relevant events were included as described in page 6 under "Observation of events".
S9	Ethical issues pertaining to human subjects	Documentation of approval by an appropriate ethics review board and participant consent, or explanation for lack thereof; other confidentiality and data security issues	P27	Information on research ethics has been provided separately from the manuscript. We have added documentation on consent forms for the baseline survey, and oral consent for the observation of events.
S10	Data collection methods	Types of data collected; details of data collection procedures including (as appropriate) start and stop dates of data collection and analysis, iterative process, triangulation of sources/methods, and modification of procedures in response to evolving study findings; rationale[b]	P5,6	These have been provided in Data collection and Data analysis sections. We would like to highlight that observation of events covered the main period of IMPALA lifetime, the observation findings were used for refining interview question and triangulating interview data.

For peer review only

S11	Data collection instruments and technologies	Description of instruments (e.g., interview guides, questionnaires) and devices (e.g., audio recorders) used for data collection; if/how the instrument(s) changed over the course of the study	P5,6	Instruments for Baseline survey, semi-structured interviews, document review and observation of events have been described respectively in the Data collection section under each research method.
S12	Units of study	Number and relevant characteristics of participants, documents, or events included in the study; level of participation (could be reported in results)	P7-9	These are reported in results
S13	Data processing	Methods for processing data prior to and during analysis, including transcription, data entry, data management and security, verification of data integrity, data coding, and anonymization/deidentification of excerpts	P5,6	In "procedure of interviews", and also in each data collection method
S14	Data analysis	Process by which inferences, themes, etc., were identified and developed, including the researchers involved in data analysis; usually references a specific paradigm or approach; rationale [b]	P5	P5 on the framework for the study;
S15	Techniques to enhance trustworthin ess	Techniques to enhance trustworthiness and credibility of data analysis (e.g., member checking, audit trail, triangulation); rationale[b]	P3,5, 6	The credibility of our findings is strengthened from having used interview and observational data from diverse interviewees and events, corroborated by document analysis.
	**Results/findings**			
S16	Synthesis and interpretatio n	Main findings (e.g., interpretations, inferences, and themes); might include development of a theory or model, or integration with prior research or theory	P10-21	Ten themes emerged from the findings.
S17	Links to empirical data	Evidence (e.g., quotes, field notes, text excerpts, photographs) to substantiate analytic findings	P10-21	We quoted quotes in Results to substantiate analytic findings.
	**Discussion**			
S18	Integration with prior work, implications, transferabilit	Short summary of main findings; explanation of how findings and conclusions connect to, support, elaborate on, or challenge conclusions of earlier scholarship; discussion of	P21-26	In Discussion section, we first provided a short summary of main findings, followed by explanation of how our

For peer review only

	y, and contribution(s) to the field	scope of application/generalizability; identification of unique contribution(s) to scholarship in a discipline or field		key findings and conclusions connect to, support, elaborate on conclusions of earlier scholarship. We also discussed our contribution in CDR analysis framework and put forward recommendations for planning and implementing future CDR work.
S19	Limitations	Trustworthiness and limitations of findings	P3	Provided in 'Strengths and limitations of this study' as required by BMJ Open.
	**Other**			
S20	Conflicts of interest	Potential sources of influence or perceived influence on study conduct and conclusions; how these were managed	P27	A competing interests statement was provided.
S21	Funding	Sources of funding and other support; role of funders in data collection, interpretation, and reporting	P27	A funding statement was provided.

[a] O'Brien, Bridget C.; Harris, Ilene B.; Beckman, Thomas J.; Reed, Darcy A.; Cook, David A. Academic Medicine89(9):1245-1251, September 2014. doi: 10.1097/ACM.0000000000000388

The authors created the SRQR by searching the literature to identify guidelines, reporting standards, and critical appraisal criteria for qualitative research; reviewing the reference lists of retrieved sources; and contacting experts to gain feedback. The SRQR aims to improve the transparency of all aspects of qualitative research by providing clear standards for reporting qualitative research.

[b]The rationale should briefly discuss the justification for choosing that theory, approach, method, or technique rather than other options available, the assumptions and limitations implicit in those choices, and how those choices influence study conclusions and transferability. As appropriate, the rationale for several items might be discussed together.

For peer review only

Manuscript title:

Identifying actions to foster cross-disciplinary global health research: a mixed-methods qualitative case study of the IMPALA programme on lung health and tuberculosis in Africa

~~Actions for fostering cross-disciplinary global health research: A qualitative research~~

Research checklist: Standards for Reporting Qualitative Research (SRQR)[a]

No.	Topic	Item	Page no.	Notes
	**Title and abstract**			
S1	Title	Concise description of the nature and topic of the study Identifying the study as qualitative or indicating the approach (e.g., ethnography, grounded theory) or data collection methods (e.g., interview, focus group) is recommended	page (p) 1	We changed the title according to ed's suggestion. Thank you.
S2	Abstract	Summary of key elements of the study using the abstract format of the intended publication; typically includes background, purpose, methods, results, and conclusions	P2	The abstract follows a structure suggested by BMJ Open
	**Introduction**			
S3	Problem formulation	Description and significance of the problem/phenomenon studied; review of relevant theory and empirical work; problem statement	P3,4	Details in Introduction: Paragraph 2
S4	Purpose or research question	Purpose of the study and specific objectives or questions	P4	Details in the last paragraph of the Introduction
	**Methods**			
S5	Qualitative approach and research paradigm	Qualitative approach (e.g., ethnography, grounded theory, case study, phenomenology, narrative research) and guiding theory if appropriate; identifying the research paradigm (e.g., postpositivist, constructivist/ interpretivist) is also recommended; rationale[b]	P5-7	We adapted a framework to frame the study including data collection and analysis
S6	Researcher characteristics and reflexivity	Researchers' characteristics that may influence the research, including personal attributes, qualifications/experience, relationship with participants, assumptions, and/or presuppositions; potential or actual interaction between researchers' characteristics and the research	P3	In "Strengths and limitations of this study" bulletin point 5, we reflected on our influence on interviewees.

For peer review only

		questions, approach, methods, results, and/or transferability		
S7	Context	Setting/site and salient contextual factors; rationale[b]	P4	We introduced the context and our case 6study programme in INTRODUCTION instead of METHODS as the context and case study helps understand our research question.
S8	Sampling strategy	How and why research participants, documents, or events were selected; criteria for deciding when no further sampling was necessary (e.g., sampling saturation); rationale[b]	P5,6	For the baseline survey, sampling was described in page 5 under "Baseline survey"; For Semi-structured interviews, relevant info was also in page 5 under "Sample selection"; For Document review, all relevant documents were included as described in page 6 under "Document review"; For "Observation of events", all relevant events were included as described in page 6 under "Observation of events".
S9	Ethical issues pertaining to human subjects	Documentation of approval by an appropriate ethics review board and participant consent, or explanation for lack thereof; other confidentiality and data security issues	P27	Information on research ethics has been provided separately from the manuscript. We have added documentation on consent forms for the baseline survey, and oral consent for the observation of events.
S10	Data collection methods	Types of data collected; details of data collection procedures including (as appropriate) start and stop dates of data collection and analysis, iterative process, triangulation of sources/methods, and modification of procedures in response to evolving study findings; rationale[b]	P5,6	These have been provided in Data collection and Data analysis sections. We would like to highlight that observation of events covered the main period of IMPALA lifetime, the observation findings were used for refining interview question and

For peer review only

				triangulating interview data.
S11	Data collection instruments and technologies	Description of instruments (e.g., interview guides, questionnaires) and devices (e.g., audio recorders) used for data collection; if/how the instrument(s) changed over the course of the study	P5,6	Instruments for Baseline survey, semi-structured interviews, document review and observation of events have been described respectively in the Data collection section under each research method.
S12	Units of study	Number and relevant characteristics of participants, documents, or events included in the study; level of participation (could be reported in results)	P7-9	These are reported in results
S13	Data processing	Methods for processing data prior to and during analysis, including transcription, data entry, data management and security, verification of data integrity, data coding, and anonymization/deidentification of excerpts	P5,6	In "procedure of interviews", and also in each data collection method
S14	Data analysis	Process by which inferences, themes, etc., were identified and developed, including the researchers involved in data analysis; usually references a specific paradigm or approach; rationale [b]	P5	P5 on the framework for the study;
S15	Techniques to enhance trustworthin ess	Techniques to enhance trustworthiness and credibility of data analysis (e.g., member checking, audit trail, triangulation); rationale[b]	P3,5, 6	The credibility of our findings is strengthened from having used interview and observational data from diverse interviewees and events, corroborated by document analysis.
	**Results/findings**			
S16	Synthesis and interpretatio n	Main findings (e.g., interpretations, inferences, and themes); might include development of a theory or model, or integration with prior research or theory	P10-21	Ten themes emerged from the findings.
S17	Links to empirical data	Evidence (e.g., quotes, field notes, text excerpts, photographs) to substantiate analytic findings	P10-21	We quoted quotes in Results to substantiate analytic findings.
	**Discussion**			
S18	Integration with prior work,	Short summary of main findings; explanation of how findings and conclusions connect to, support,	P21-26	In Discussion section, we first provided a short summary of main

For peer review only

	implications, transferability, and contribution(s) to the field	elaborate on, or challenge conclusions of earlier scholarship; discussion of scope of application/generalizability; identification of unique contribution(s) to scholarship in a discipline or field		findings, followed by explanation of how our key findings and conclusions connect to, support, elaborate on conclusions of earlier scholarship. We also discussed our contribution in CDR analysis framework and put forward recommendations for planning and implementing future CDR work.
S19	Limitations	Trustworthiness and limitations of findings	P3	Provided in 'Strengths and limitations of this study' as required by BMJ Open.
	**Other**			
S20	Conflicts of interest	Potential sources of influence or perceived influence on study conduct and conclusions; how these were managed	P27	A competing interests statement was provided.
S21	Funding	Sources of funding and other support; role of funders in data collection, interpretation, and reporting	P27	A funding statement was provided.

[a] O'Brien, Bridget C.; Harris, Ilene B.; Beckman, Thomas J.; Reed, Darcy A.; Cook, David A. Academic Medicine89(9):1245-1251, September 2014. doi: 10.1097/ACM.0000000000000388

The authors created the SRQR by searching the literature to identify guidelines, reporting standards, and critical appraisal criteria for qualitative research; reviewing the reference lists of retrieved sources; and contacting experts to gain feedback. The SRQR aims to improve the transparency of all aspects of qualitative research by providing clear standards for reporting qualitative research.

[b]The rationale should briefly discuss the justification for choosing that theory, approach, method, or technique rather than other options available, the assumptions and limitations implicit in those choices, and how those choices influence study conclusions and transferability. As appropriate, the rationale for several items might be discussed together.

For peer review only
